# P–P Coupling *with* and *without* Terminal Metal–Phosphorus Intermediates

**DOI:** 10.1021/jacs.4c16833

**Published:** 2025-01-28

**Authors:** Richard R. Thompson, Matthew T. Figgins, Duleeka C. Wannipurage, Angel Renteria-Gomez, Achyut Ranjan Gogoi, Joshua Telser, David L. Tierney, Marc C. Neben, Serhiy Demeshko, Osvaldo Gutierrez, David C. Powers

**Affiliations:** † Department of Chemistry, Texas A&M University, College Station, Texas 77843, United States; x Department of Chemistry, University of Idaho, Moscow, Idaho 83844, United States; ∇ Department of Biological, Physical and Chemical Science, Roosevelt University, Chicago, Illinois 60605, United States; £ Department of Chemistry and Biochemistry, Miami University, Oxford, Ohio 45056, United States; € Institut für Anorganische Chemie, Georg-August-Universität, Tammannstrasse 4, Göttingen 37077, Germany

## Abstract

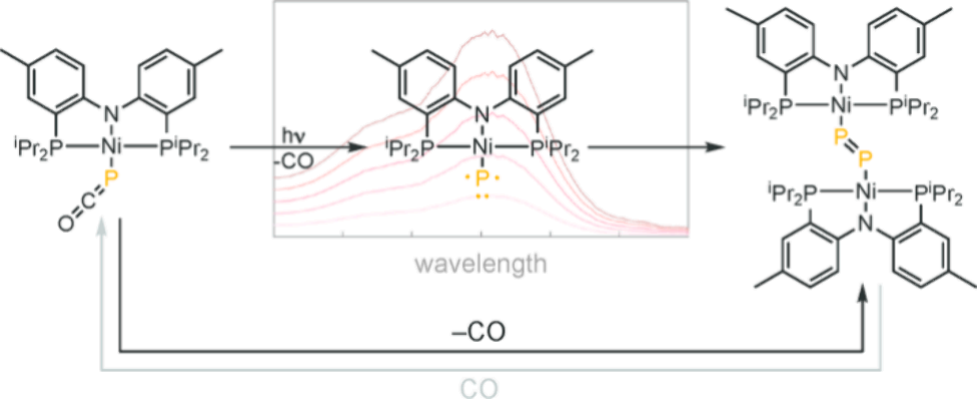

Terminal metal–phosphorus (M–P) complexes are of significant contemporary interest as potential platforms for P-atom transfer (PAT) chemistry. Decarbonylation of metal–phosphaethynolate (M–PCO) complexes has emerged as a general synthetic approach to terminal M–P complexes. M–P complexes that are stabilized by strong M–P multiple bonds are kinetically persistent and isolable. In the absence of strong M–P stabilization, the formation of diphosphorus-bridged complexes (i.e., M–P–P–M species) is often interpreted as evidence for the intermediacy of reactive, unobserved M–P species. Here, we demonstrate that while diphosphorus complexes can arise from reactive M–P species, P–P coupling can also proceed directly from M–PCO species without the intermediacy of M–P complexes. Photochemical decarbonylations of a pincer-supported Ni (II)–PCO complex at 77 K afford a spectroscopically observed terminal Ni–P complex, which is best described as a triplet, Ni(II)-metallophosphinidene with two unpaired electrons localized on the atomic phosphorus ligand. Thermal annealing of this transient Ni–P complex results in rapid dimerization to afford the corresponding P_2_^2–^-bridged dinickel complex. Unexpectedly, the same P_2_^2–^-bridged dinickel complex can also be accessed via a thermally promoted process in the absence of light. The analysis of reaction kinetics, isotope-labeling studies, and computational results indicate that the thermal P–P coupling process proceeds via a noncanonical mechanism that avoids terminal M–P intermediates. Together, these results represent the first observation of P–P coupling from characterized terminal M–P species and demonstrate that terminal M–P intermediates are not required to obtain P–P coupling products. These observations provide critical mechanistic understanding of the activation modes relevant to P-atom transfer.

## Introduction

Reactive metal–ligand (M–L) multiply bonded species, such as metal oxo,^1,2^ nitrido,^2−5^ and nitrene complexes,^6−8^ underpin many modern C–H functionalization strategies. In the case of oxygen- and nitrogen-transfer schemes, the availability of predictable synthetic methods to generate reactive M–L intermediates, via well-established oxygen- and nitrogen-transfer reagents, has enabled rational development of robust catalytic chemistry.^3,8−11^ Despite their potential synthetic impact, atom- and group-transfer processes are much less established for heavier elements.^12−16^ The relative dearth of molecular platforms for C–H activation at heavier M–L complexes stands in contrast to the activity of related heterogeneous catalysts: Metal phosphides, for example Ni_2_P, display activity in hydrogen evolution,^17^ hydrodesulfurization,^18^ and alkane dehydrogenation activity.^19−23^

**Figure 1:**
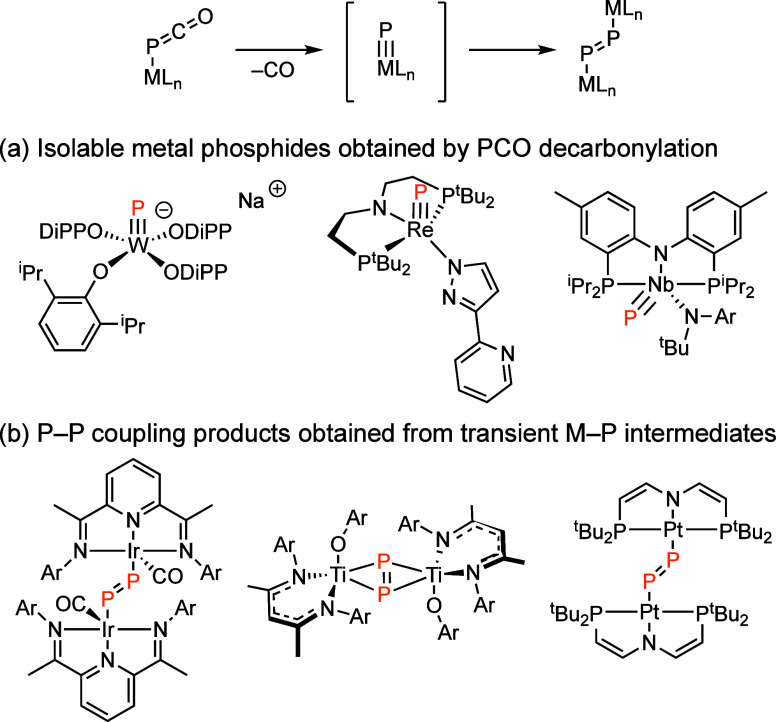
Decarbonylation of M–PCO complexes has emerged as a general synthetic approach to M–P species. (a) CO elimination from M–PCO complexes has enabled synthesis and isolation of metal phosphide complexes of tungsten, rhenium, and niobium. (b) Isolation of diphosphorus complexes has been advanced as evidence of the intermediacy of transient M–P species.

The phosphaethynolate (PCO^–^) anion—first reported in 1992 by Becker^24^ and made widely available in 2014 by Grützmacher^25^—has invigorated synthetic metal phosphide chemistry and P atom transfer (PAT) research.^26−28^ These efforts are guided by the analogy between azide ligands, which undergo facile N_2_ loss to generate metal nitrides,^29−42^ and phosphaethynolate ligands, which undergo CO loss to generate metal phosphides.^43−46^ Two broad families of M–P species have been reported: *Isolable M–P complexes*, such as those of W,^45^ Re,^46^ and Nb,^44^ pictured in Figure 1a, are stabilized by strong M–P triple bonds and are kinetically persistent. *Reactive M–P complexes*, for example those which cannot achieve M–P triple bonds, are typically not observed but are instead inferred based on the isolation of P–P coupling products (Figure 1b).^47−50^ At present, P–P coupling from isolated or observed M–P species has not been experimentally demonstrated.^51^ In cases where diphosphorus products are observed from isolated M–P species, coupling occurs via transient species generated by oxidation.^46,52^ Other mechanisms for P–P coupling have been evaluated computationally but not demonstrated experimentally.^53^ Detailed understanding of potential reaction pathways available to PCO activation is critical to the rational development of P-atom transfer chemistry and catalysis.

**Figure 2:**
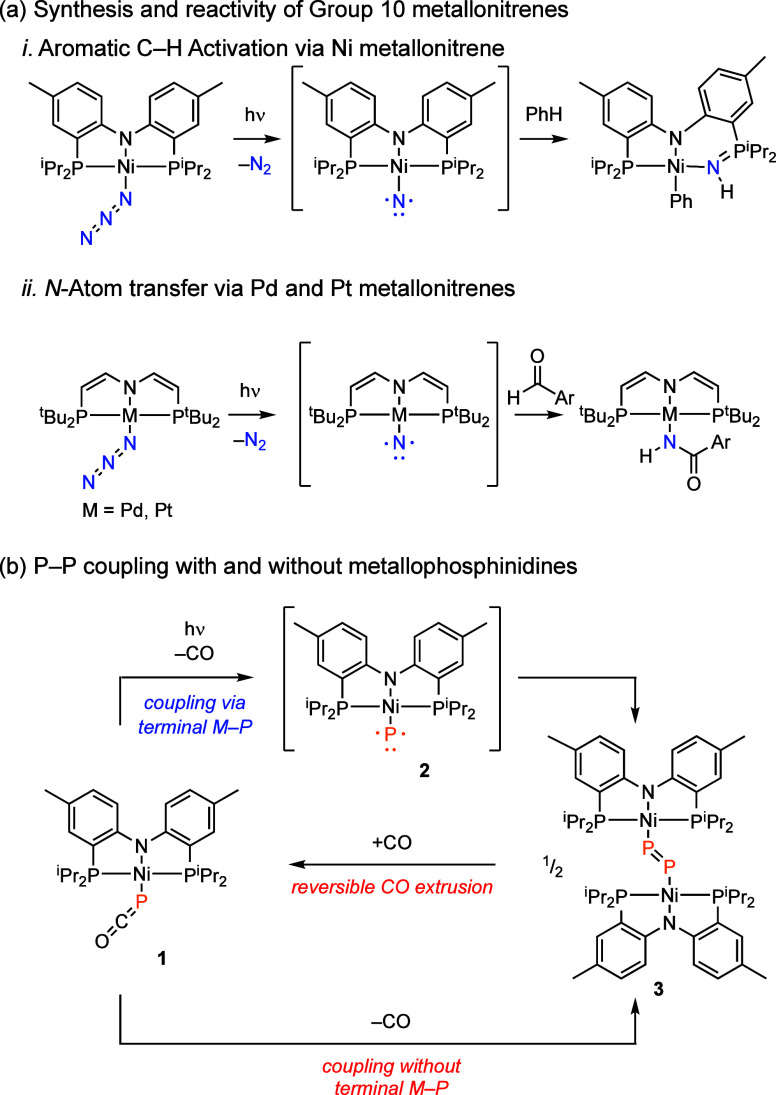
(a) Chemistry of Group 10 metallonitrenes. *i.* Photolysis of (PNP)Ni(N_3_) in PhH results in N_2_ elimination and C–H activation products via a putative Ni metallonitrene. *ii.* Photolysis of (PNP)M(N_3_) complexes of Pd and Pt affords the corresponding triplet metallonitrenes, which have been observed spectroscopically and via *in crystallo* methods, and which effect *N*-atom transfer (NAT) to aldehydic C–H bonds. (b) Here, we report the cryogenic photolysis of Ni–PCO complex **1** generates triplet Ni metallophosphinidene **2**, which undergoes rapid dimerization of the diphosphorus complex **3**. In addition, we demonstrate a facile and reversible thermal process for P–P coupling without M–P intermediates.

Motivated by 1) van der Vlugt’s report of a highly electrophilic (and transient) Ni metallonitrene (i.e., Ni–N) generated by N_2_ extrusion from the corresponding PNP-supported Ni(II) azide precursor (Figure 2a),^29^ and, 2) the demonstrated role of PNP-supported Pd and Pt metallonitrenes in nitrogen-atom transfer (NAT) reactions with aldehydic C–H bonds to generate benzylamides (Figure 2b)^40,41^ and with styrenes to generateimines,^54^ we report the synthesis and characterization of Ni(II)–PCO complex **1**. Cryogenic photolysis of **1** unveils a reactive Ni–P species (**2**), which we formulate as a triplet metallophosphinidene based on a combination of cryogenic spectroscopy and computational results. In the absence of an appropriate substrate, rapid P–P coupling occurs to afford dinickel complex **3**; in the presence of an appropriate substrate, namely a source of weak C–H bonds, H-atom abstraction (HAA) is observed to generate the corresponding Ni–PH_2_ complex. Unexpectedly, we also observed that Ni(II)–PCO complex **1** thermally evolves to P_2_-bridged complex **3** despite the prohibitively high computed CO extrusion barrier (∼40 kcal/mol). This observation led to the discovery and validation of a nonphosphide mechanism for P_2_ synthesis. Analysis of reaction kinetics, isotope labeling studies, and computational investigations indicate thermal P_2_ synthesis via a decarbonylative dimerization pathway that avoids Ni–P intermediate **2**. The thermal pathway is reversible: Treatment of the P_2_-bridged complex **3** with CO regenerates Ni–PCO complex **1**. These observations 1) provide characterization of the first terminal M–P complex of Ni, 2) validate P–P coupling from an observed M–P intermediate, 3) demonstrate that the observation of diphosphorus products does not imply the intermediacy of M–P species, and 4) define the mechanistic landscape available to PCO activation and P-atom transfer catalysis.

## Results and Discussion

### Synthesis and Photochemistry of (PNP)Ni(PCO) (**1**)

**Figure 3:**
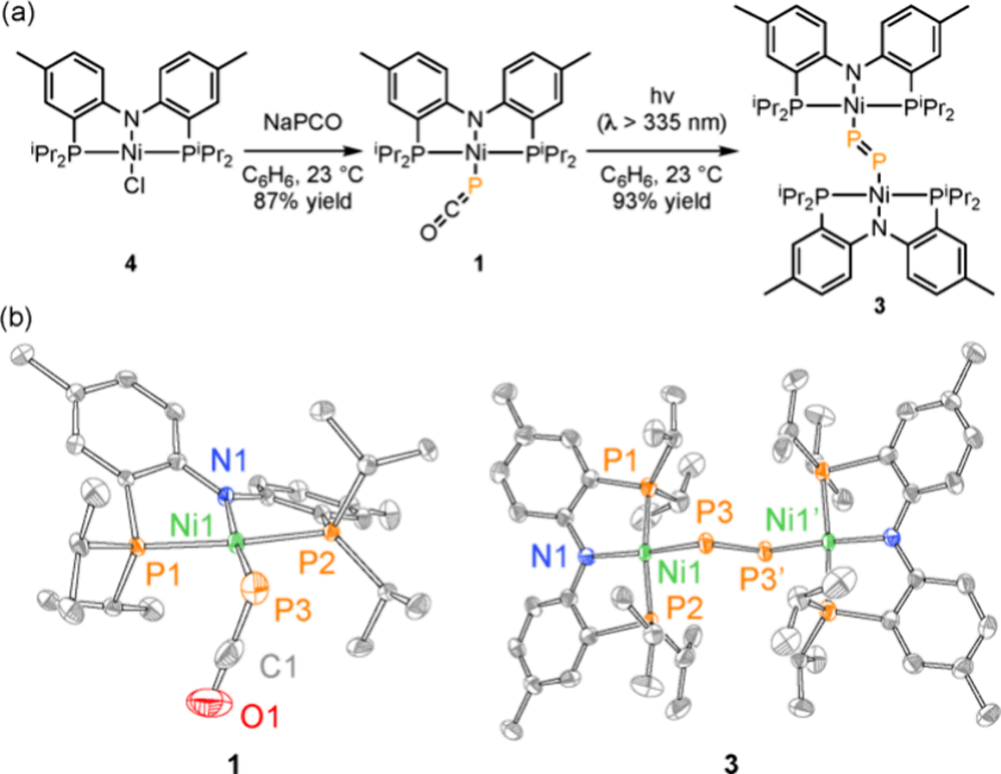
(a) Synthesis of Ni phosphaethynolate **1** by ligand metathesis from Ni chloride **4**. Photochemical decarbonylation affords dinickel diphosphorus complex **3**. (b) Displacement ellipsoid plots of **1** and **3** at the 50% probability level with H atoms, cocrystallized toluene molecule for **1**, and disordered atoms for **3** omitted for clarity. Selected metrics for **1**, Ni1–P3 2.266(1) Å, P3–C1 1.594(6) Å, C1–O1 1.204(7) Å, Ni1–P3–C1 89.2(2)°; for **3,** Ni1–P3 2.197(1) Å, P3–P3′ 2.027(2) Å, Ni1–P3–P3′ 115.24(9)°.

Dark blue-green (PNP)Ni(PCO) (**1**) was prepared in 87% yield by treatment of the corresponding chloride complex (**4**)^55^ with NaPCO(dioxane)_n_^25,56^ in benzene (Figure 3a). Complex **1** is C_2_-symmetric by ^1^H NMR spectroscopy, with single resonances for both the PNP-borne isopropyl methine and tolyl methyl positions, respectively (Figure S1). The ^31^P{^1^H} spectrum features two coupled signals (^2^*J*_PP_ = 20.8 Hz) in a 2:1 ratio centered at 38 ppm and −393 ppm, which we ascribe to the phosphorus nuclei of the PNP and PCO ligands, respectively (Figure S2). The FT-IR spectrum of **1** recorded in a KBr pellet displays a signal at 1869 cm^–1^, which is consistent with the asymmetric stretching mode of a *P*-bound PCO ligand (Figure S3); *O*-bound PCO ligands typically display asymmetric stretching modes between 1650 and 1700 cm^–1^.^27^ The UV–vis spectrum of a toluene solution of **1** displayed strong absorbances centered at 323 and 349 nm (ε = 5.9 × 10^3^ and 6.5 × 10^3^ M^–1^cm^–1^, respectively) and a Laporte-forbidden, metal-based absorbance centered at 577 nm (ε = 1.7 × 10^2^ M^–1^cm^–1^) (Figure S4).

Single-crystal X-ray diffraction (SCXRD) analysis of **1** confirms the molecular structure of **1** to be *P*-bound PCO adduct (Figure 3b), with a Ni–P bond length of 2.266(1) Å and a Ni–P–C bond angle of 89.2(2)°. The measured Ni–P–C angle is significantly more acute than the corresponding angle in (PNP)Ni(N_3_) of 120.6°,^29^ which is consistent with the larger energy gap and attendant decreased hybridization of the 3s and 3p orbitals of P relative to N.^57^

Photolysis (λ > 335 nm) of a blue C_6_D_6_ solution of **1** results in the rapid conversion to a dark purple-brown solution of diphosphorus-bridged dinickel complex **3** (93% yield based on ^31^P{^1^H} NMR). The ^31^P{^1^H} NMR spectrum obtained following photolysis features a sharp singlet at 30 ppm and a broad singlet (Δν_1/2_ = 29 Hz) centered at 762 ppm, which we assign as the PNP-based phosphorus atoms and a bridging, dianionic diphosphorus ligand (i.e., P_2_^2–^),^47,49^ respectively. The obtained Raman spectrum displays a vibrational mode at 577 cm^–1^, which is consistent with the P=P stretching mode reported by Schneider for a related diplatinum P_2_-bridged complex (582 cm^–1^).^49^ SCXRD analysis confirmed the molecular structure of **3** to be a dinickel(II) complex containing an η^1^,η^1^-bridging P_2_^2–^ moiety (Ni1–P3–P3′–Ni1’ = 180°; Ni1–P3–P3′ = 115.24°) (Figure 3b). While a number of P_2_-bridged dinickel complexes have been reported, all previous examples feature η^2^,η^2^-coordination of the P_2_^2–^ fragment.^50,58−62^ The P–P distance in **3** (2.027(2) Å) is the shortest reported of the Ni_2_(P_2_) moieties, indicating a reduced extent of activation.

**Figure 4:**
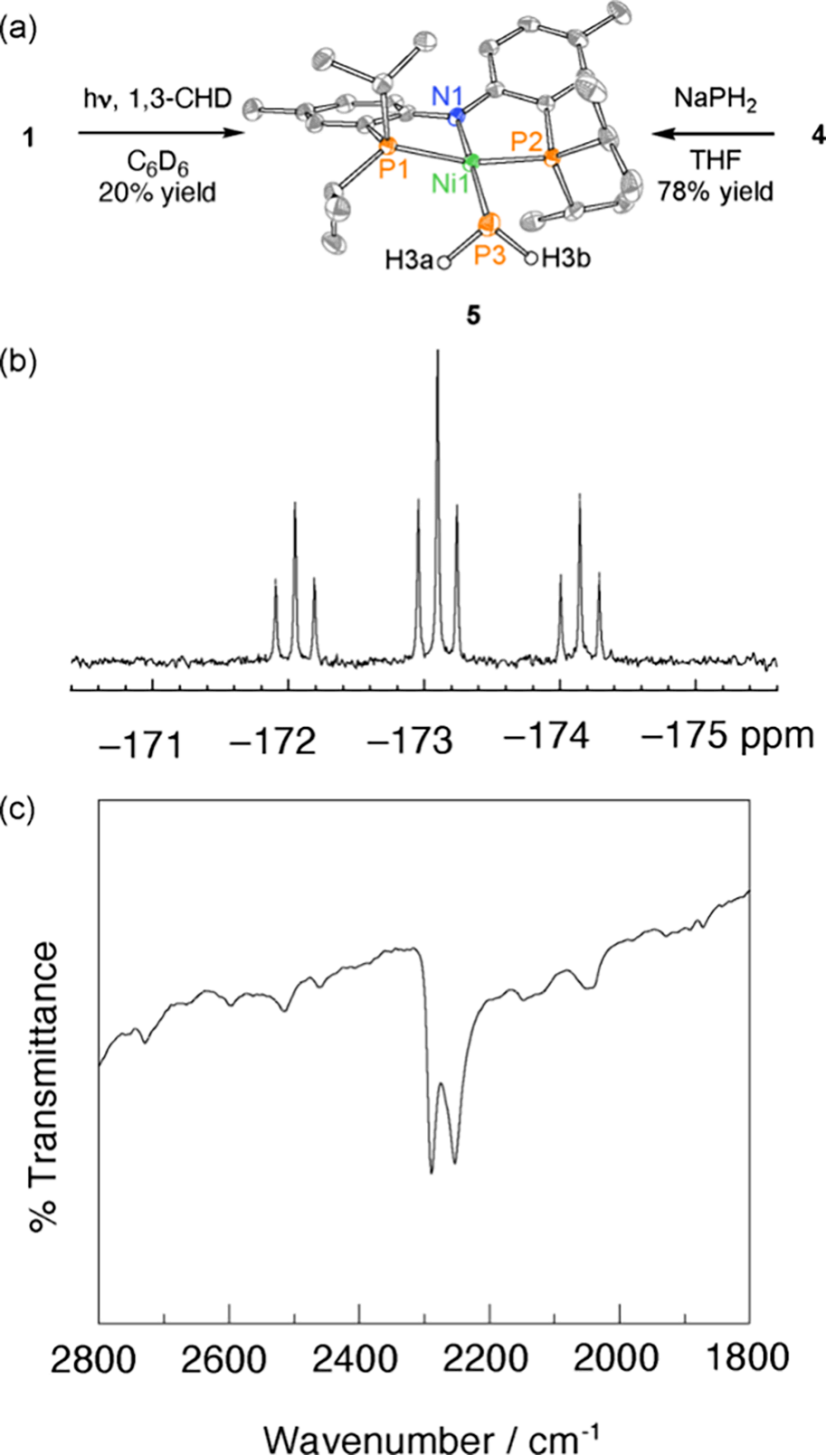
(a) Photolysis of **1** in the presence of 1,3-cyclohexadiene (1,3-CHD) affords phosphinide **5**, which can be independently prepared by treatment of (PNP)Ni(Cl) **4** with NaPH_2_. Displacement ellipsoid plot of **5** drawn at the 50% probability level with H atoms, except for the PH_2_ fragment, and cocrystallized toluene molecule omitted for clarity. Selected metrical parameters: Ni1–P3 2.2082(9) Å, N1–Ni1–P3 175.60(9)°, P1–Ni1–P2 165.51(2)°. (b) The ^31^P NMR spectrum of **5** displays a resonance at −173 ppm (^1^J_PH_ = 171 Hz, ^3^J_PP_ = 23 Hz), which we attribute to the phosphinide P atom. (c) The FT-IR spectrum of **5** displays P–H stretching frequencies at 2253 and 2289 cm^–1^.

Addition of 1,3-cyclohexadiene (100 equiv) to the photolysis of **1** suppresses the formation of **3** (from 93% to 22% yield) and results in the formation of a number of new products as evidenced by ^31^P{^1^H} NMR (Figure 4a, Figure S5). Of particular interest is the formation of Ni–PH_2_ complex **5** (20% yield), which is characterized by a coupled doublet (42 ppm) and triplet (−173 ppm) in 2:1 ratio (^2^J_PP_ = 23 Hz). The proton-coupled ^31^P NMR spectrum revealed the signal at −173 ppm to be a triplet of triplets (^1^J_PH_ = 171 Hz; Figure 4b). The FT-IR spectrum of **5** displays absorbances at 2253 and 2289 cm^–1^ which we assign as the P–H stretches (Figure 4c). These spectral features are similar to those previously reported for related Zr–PH_2_^63^ and Ni–PH_2_ complexes.^64^ SCXRD analysis confirms the formulation of **5** as a Ni(II) phosphinide, which displays a distorted square planar geometry (P1–Ni1–P2 165.51(2)°, N1–Ni1–P3 175.60(9)°) with a Ni–P3 distance of 2.2082(9) Å (Figure 4a). The assignment of compound **5** was further confirmed by independent synthesis from (PNP)Ni(Cl) **4** and NaPH_2_ (78% yield).^65^

While photolysis of **1** in the presence of 1,3-cyclohexadiene (C–H bond dissociation energy (BDE) = 74.3 kcal/mol) afforded phosphinide complex **5**, photolysis in the presence of triphenylmethane (81.0 kcal/mol), 1,4-cyclohexadiene (76.0 kcal/mol), or 9,10-dihydroanthracene (75.3 kcal/mol) did not.^66^ These experiments indicate a relatively weak P–H bond in **5**, which is consistent with recent reports from Agapie et al. regarding a P–H BDE of 59 kcal/mol in the context of a dimolybdenum phosphinide.^67^ Our own computational efforts (at the UB3LYP-D3/6-311+g(d,p)-CPCM(benzene)//UB3LYP-D3/def2svp-CPCM(benzene) level of theory) found the BDE of the first P–H bond in **5** (i.e., Ni–PH_2_ (**5**) to Ni–PH) to be 65.2 kcal/mol and the second P–H BDE (i.e., Ni–PH to Ni–P (**2**))to be 65.3 kcal/mol (see Supporting Information Section G). Of note, complex **5** represents a rare example of a crystallographically characterized metal phosphinide and the first example of a M–PH_2_ complex obtained by H-atom abstraction chemistry. M–PH_2_ complexes are more typically obtained by deprotonation of the corresponding M–PH_3_ adducts,^68−71^ by oxidative addition of PH_3_,^64,72−74^ or by reaction of metal halide complexes with metal phosphinide salts (M–PH_2_; M = Na, K).^63,75,76^

### Characterization of Transient Ni Phosphinidene **2**

Based on the observation of P–P coupling product **3** (Figure 3) and HAA product **5** (Figure 4) following photoactivation of Ni–PCO complex **1**, we speculated that photolysis of **1** resulted in the formation of a transient Ni–P species, i.e., **2**. Initial support for this hypothesis (and evidence for facile decarbonylation of **1**) was obtained by atmospheric-pressure chemical ionization mass spectrometry (APCI-MS) measurements of **1**(*m*/*z* = 518.18), which corresponds to the mass expected for the [(PNP)NiP(H)]^+^ fragment (i.e., **2**(H)^**+**^).^77^

**Figure 5:**
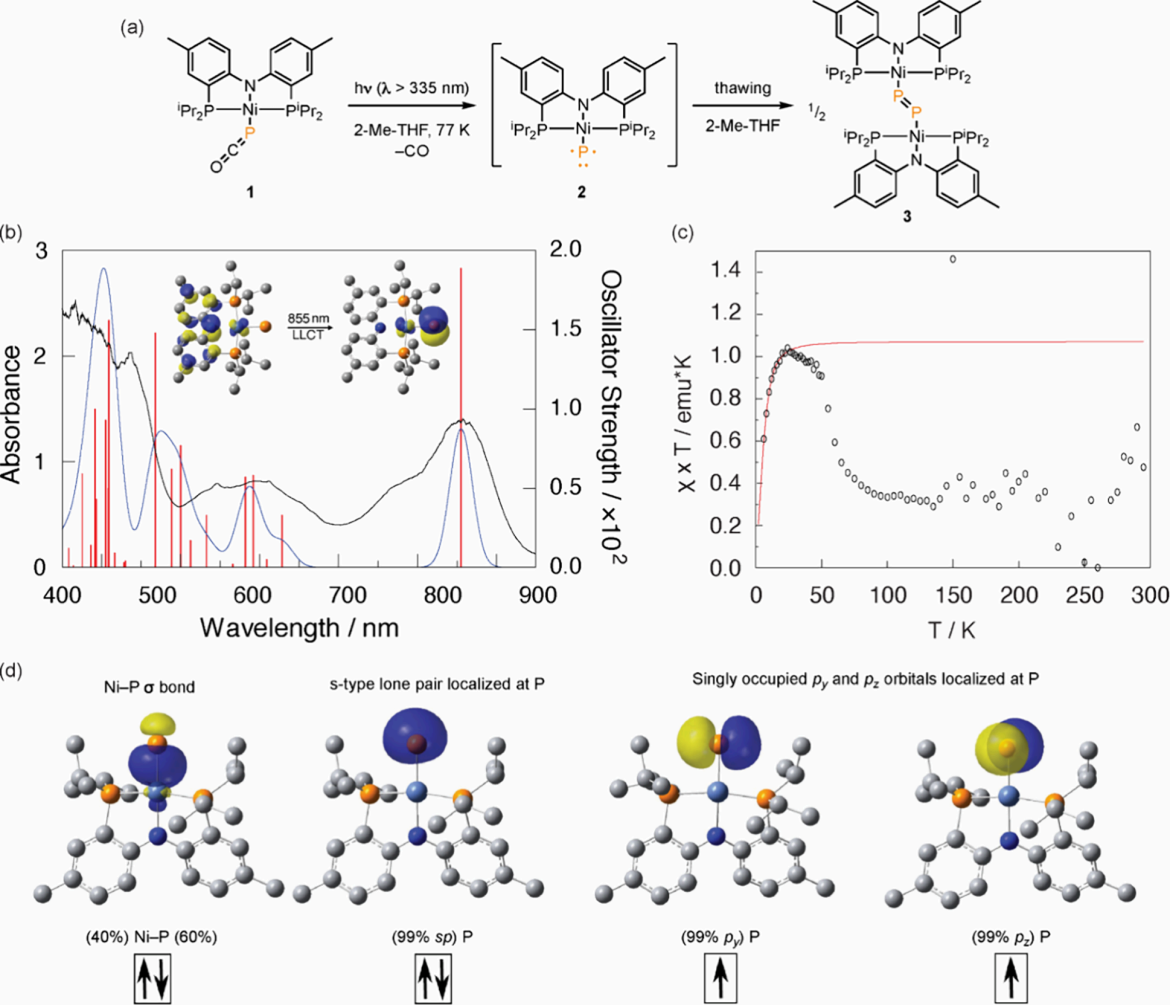
(a) Photolysis of a 2-Me-THF glass of **1** at 77 K affords metallophosphinidene **2**, which dimerizes to afford diphosphorus compound **3** upon thawing. (b) UV–vis absorption spectrum of **2** (77 K, black line). Simulated absorption spectrum obtained from TD–DFT calculations of ^3^[**2**] (blue line) with vertical excitations indicated with red bars. Inset: Most significant contributors to the computed transition at 855 nm is LLCT from the amide to the phosphinidene. TD–DFT computed at the UB3LYP-D3BJ/def2-TZVP-SMD(Benzene)//UB3LYP-D3BJ/Def2-TZVP level of theory. (c) Plot of χT vs T obtained following photolysis (350 nm at 10 K) of **1**. (d) Orbitals obtained by NBO analysis of ^3^[**2**] indicate a covalent Ni–P σ bond as well as an s-type lone pair and two singly occupied p-type orbitals on the atomic phosphorus ligand.

The formation of a transient photointermediate, which we assign as Ni metallophosphinidene **2**, was confirmed by cryogenic UV–vis spectroscopy (Figure 5a). Photolysis of a 2-Me-THF glass of **1** at 77 K resulted in the disappearance of absorbances characteristic of **1** and the growth of new spectral features centered at 435 nm (ε = 4.4 × 10^2^ M^–1^cm^–1^), 610 nm (ε = 1.0 × 10^2^ M^–1^cm^–1^), 773 nm (ε = 1.7 × 10^2^ M^–1^cm^–1^), and 858 nm (ε = 3.8 × 10^2^ M^–1^cm^–1^) (Figure 5b).^78^ Upon thawing, the features ascribed to complex **2** disappear and are replaced by those of diphosphorus complex **3** (Figure S6). The formation of **3** upon thermal annealing of the low-temperature photolysis reaction was confirmed by ^31^P{^1^H} NMR spectroscopy (Figure S7).

The cryogenic UV–vis spectrum pictured in Figure 5b is well-matched with time-dependent density functional theory (TD-DFT) calculations of a triplet metallophosphindene (i.e., ^3^[**2**]; for comparison of the cryogenic UV–vis data with TD-DFT results for ^1^[**2**], see Figure S8). DFT optimized geometries of **2** in either singlet or triplet configurations displayed square-planar coordination at Ni. Domain-based local pair natural orbital coupled cluster method with single and double and perturbative triple excitations (DLPNO–CCSD(T)) calculations revealed that the triplet state is lower in energy than singlet by 13.9 kcal/mol. The triplet ground state was confirmed by magnetic measurements: Photolysis (λ = 350 nm, T = 10 K) of a solid sample of **1** within the cavity of a SQUID magnetometer was monitored by measuring the DC moment of the sample as a function of time (Figure S9). The resulting sample was subjected to a temperature-dependent measurement (2 to 295 K) at a magnetic field of 5000 Oe (Figure 5c). The data obtained for T ≤ 20 K could be satisfactorily fit with *g* = 2.07 (fixed for S = 1) and axial zero-field splitting (zfs) parameter |D| = 14.3 cm^–1^. Low-temperature decomposition, similar to that observed here, have been noted in other solid-state magnetic measurements of photogenerated solid-state samples, and may reflect the sample matrix (i.e., microcrystalline powder) used for this experiment.^49^ Consistent with a triplet ground state with zfs of 14.3 cm^–1^, attempts to observe **2** by X-band EPR spectroscopy up to 1.45 T were unsuccessful.^79,80^ Attempts to observe **2** by single-crystal photolysis, which we^40,81−85^ and others^41,86,87^^88^ have applied to the synthesis of M–N, M–NR, and M–CR complexes, were also unsuccessful.

Natural Bond Orbital (NBO) analysis indicates ^3^[**2**] displays a covalent Ni–P σ bond that is polarized toward P as well as an s-type lone pair and two singly occupied p-type NBOs on the atomic phosphorus ligand (Figure 5d), which is analogous to the electronic structures of the triplet Pt and Pd metallonitrenes introduced by Holthausen and Schneider.^40,41,39^ Analysis of the orbital parentage of the observed low-energy absorption in the UV–vis spectrum is consistent with a ligand-to-ligand charge transfer (LLCT) between the amide nitrogen of the PNP ligand and the atomic phosphorus ligand (Figure 5b inset).

### Thermal Activation of PCO Complex **1**

**Figure 6:**
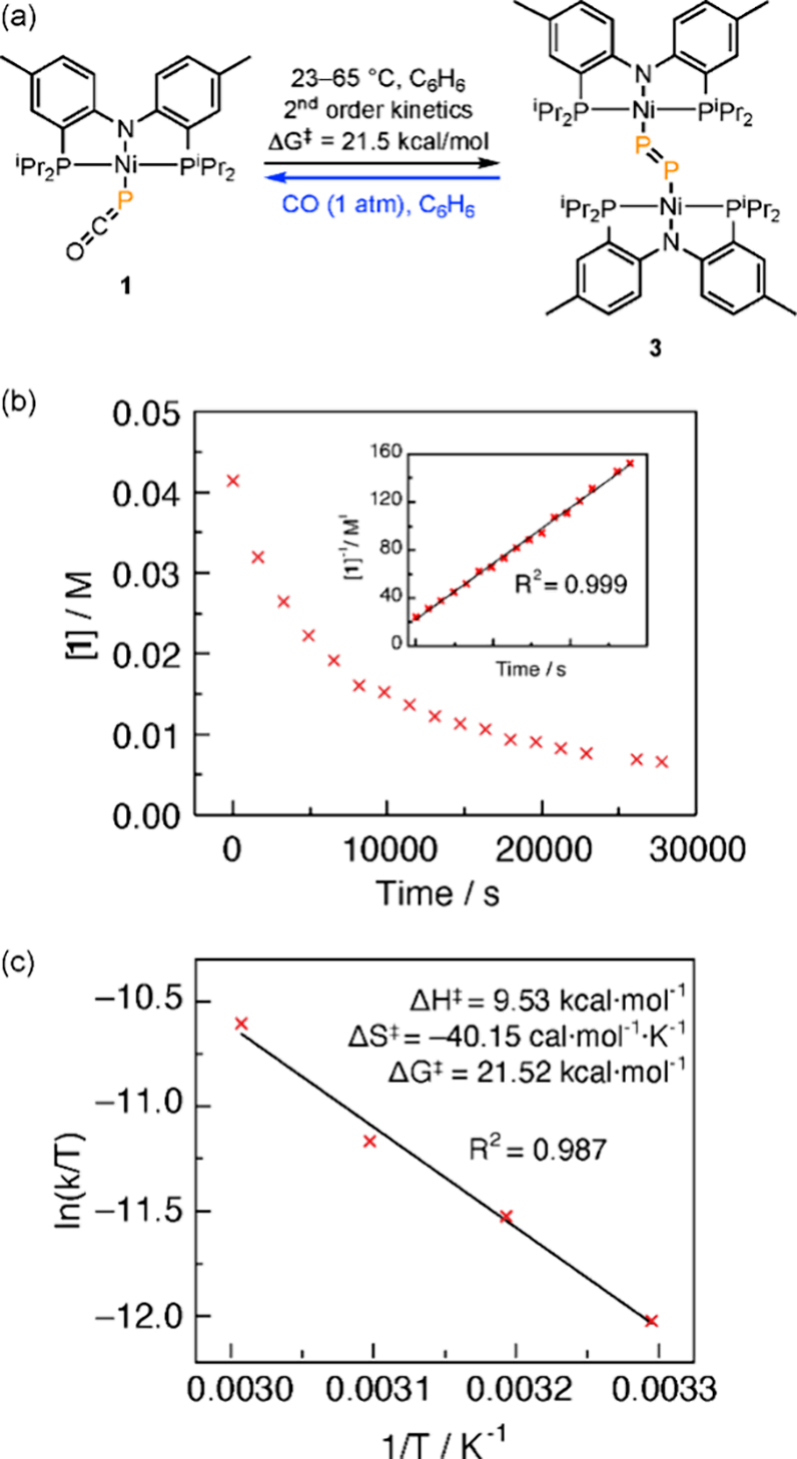
(a) Thermolysis of **1** in the exclusion of light affords **3.** Decarbonylation is reversible as treatment of **3** with CO regenerates complex **1**. (b) Plot of [**1**] vs time at 55 °C. Inset: Plot of [**1**]^−1^ vs time at 55 °C is linear, indicating a second-order rate law for the consumption of **1**. (c) Eyring plot formulated from temperature-dependent kinetics experiments for the conversion of **1** to **3** enabled determination of activation parameters for the thermal decarbonylation of **1**.

Thermal decarbonylation of phosphaethynolate complex **1** to generate metallophosphinidene **2** confronts prohibitive barriers on either the singlet or triplet surfaces (Δ*G*^‡^ ≥ 39.9 kcal/mol, *vide infra*). Thus, we were surprised that solutions of **1** gradually afforded diphosphorus complex **3** at ambient temperature over the course of 7 days in the rigorous exclusion of light; heating solutions of **1** to 60 °C resulted in conversion of **3** over the course of 6 h (65% yield based on ^31^P{^1^H} NMR; 37% crystallized yield; Figure 6a). The incongruity between the computed barriers for thermal decarbonylation of **1** and the facility of thermal formation of **3** led us to speculate that a nonphosphide mechanism might also be available for the synthesis of **3**.

We monitored the thermal conversion of complex **1** to **3** as a function of time by ^31^P{^1^H} NMR spectroscopy at 55 °C under an N_2_ atmosphere (Figure 6b). A plot of 1/[**1**] vs time is linear (Figure 6b, inset), which indicates a second-order rate law for the conversion of **1** to **3**; see Figure S10 for similar plots of zeroth- and first-order rate laws. An Eyring plot was constructed from temperature-dependent kinetics measurements (35–65 °C) and provided activation parameters of Δ*H*^‡^ = 9.5 kcal/mol, Δ*S*^‡^ = −40.2 cal/(mol·K) and Δ*G*^‡^_298.15K_ = 21.5 kcal/mol. The highly negative entropy of activation is consistent with dimerization of two equivalents of **1** in the rate-limiting step for the thermal generation of **3**.^36,89^ These results indicate that a facile bimolecular mechanism is available for thermally promoted conversion of **1** to **3**. See Supporting Information Section E for discussion of the impact of CO pressure on P–P coupling.

Computational evaluation of the dimerization of **1** to generate **3** was carried out by dispersion-corrected density functional theory at UB3LYP-D3/6-311+g(d,p)-CPCM(benzene)//UB3LYP-D3/def2svp-CPCM(benzene) level of theory (see Supporting Information for computational details). Consistent with the experimentally determined second-order rate law for this reaction, the rate-determining transition state was identified as **TS**_**1,6**_, in which the P atom of one equivalent of **1** is transferred to the PCO unit of the other while the corresponding P–CO bond is elongating. The difference in the computed barrier height (31.0 kcal/mol) and the experimentally determined barrier (21.5 kcal/mol) is likely due to improper computational treatment of CO egress from the reaction solvent.^90,91^ By intrinsic reaction coordinate (IRC) analysis, transition state **TS**_**1,6**_ is on-path to dinickel complex **6**, in which the two Ni ions are bridged by a three-membered P_2_C(O) ring. Subsequent decarbonylation of **6** proceeds via transition state **TS**_**6,3**_ to afford complex **3**, the product of P–P coupling, without the intermediacy of a terminal Ni–P species (i.e., **2**). Consistent with the relative energies of **TS**_**1,6**_ and **TS**_**6,3**_, we have not observed dinickel intermediate **6** experimentally.

### Reversibility of Thermal CO Extrusion from **1**

Consistent with the facility of thermal P–P coupling that connects complexes **1** and **3**, decarbonylation of **1** is thermally reversible: Exposure of a C_6_D_6_ solution of complex **3** to 1 atm of CO results in the formation of **1** (32% yield based on ^31^P{^1^H} NMR analysis).^92^ To further investigate the reversible decarbonylation of **1**, treatment of **3** with ^13^CO led to the formation of ^13^C-**1** (Figure 8a). ^31^P{^1^H} NMR analysis revealed the signals expected for **1** with additional ^13^C–^31^P coupling for both the PNP (^3^J_CP_ = 5 Hz) and PCO (^1^J_CP_ = 99 Hz, Figure 8b). HR-MS(+) further confirmed the formation of the heavier isotopomer (Figure 8c; predicted mass = 547.1783 amu; observed mass = 547.1775 amu). The ATIR spectrum of the crude material gave further evidence for ^13^C-incorporation with a stretching frequency of 1823 cm^–1^, in good agreement with the reduced mass, harmonic oscillator model (computed frequency = 1825 cm^–1^). The addition of CO to **3** to regenerate **1** represents the first example of diphosphorus carbonylation to afford a metal phosphaethynolate complex.

Examination of the orbital interactions within transition state TS_6,3_ reveals the origin of the facile carbonylation of the P_2_^2–^ ligand in complex **3** (i.e., the microscopic reverse of the decarbonylation of **6**). The approach of the CO toward the P_2_ is asymmetric with P–C distances of 1.716 and 2.773 Å, respectively, and a C–P–P bond angle of 89.41°. The high asymmetry in approach suggests a nonlinear, cheletropic reaction is operative, with P–C bond construction arising as a result of donation from the orthogonal, filled π*/lone pairs^93^ of the P_2_ into the unfilled π* orbitals of the CO (Figure 9).

## Conclusions

Over the last ∼10 years, the decarbonylation of M–PCO complexes to generate M–P species has been intensely developed as a platform to access terminal M–P complexes. While heavily stabilized M≡P complexes can be isolated and characterized, reactive M–P species, which represent potential intermediates in an as-yet-unknown PAT catalysis, are typically inferred based on the observation of P–P coupling products. This assumption is motivated, at least in large part, by the lack of alternate mechanistic proposals for PCO activation and P–P coupling. These mechanistic details, however, are critical for rational development of potential catalytic platforms: If, for example, the P–P coupling process proceeds without the intermediacy of M–P species, the intermediates that are assumed to give rise to this chemistry may not be accessible and thus relevant to PAT schemes.

With interest in studying the reactivity of transient M–P complexes, we targeted synthesis of Ni metallophosphinidene **2** by decarbonylation of Ni–PCO precursor **1**. These studies were guided by seminal contributions by van der Vlugt^29^ and Holthausen and Schneider^39−41,54^ regarding the reactivity of Group 10 metallonitrenes, which exhibit *N*-atom electrophilicity by virtue of ligand subvalence. Photolysis of **1** results in P–P coupling to diphosphorus compound **3** (Figure 3). In contrast, photolysis in the presence of a substrate with weak C–H bonds (i.e., 1,3-cyclohexadiene), phosphinide **5** is obtained. Complex **5** arises via an HAA reaction that is without precedent in transition metal phosphorus chemistry (Figure 4).

The observed photopromoted reactivity proceeds at highly reactive metallophosphinidene **2**, which can be experimentally characterized under cryogenic conditions (Figure 5). Magnetic measurements and computational data indicate that transient intermediate **2** is a ground state triplet that we formulate as a metallophosphinidene with two unpaired electrons on an atomic phosphorus ligand. The significant spin density localized on the atomic phosphorus ligand may be responsible for the facility of direct P–P coupling chemistry and H-atom abstraction activity.

**Figure 7:**
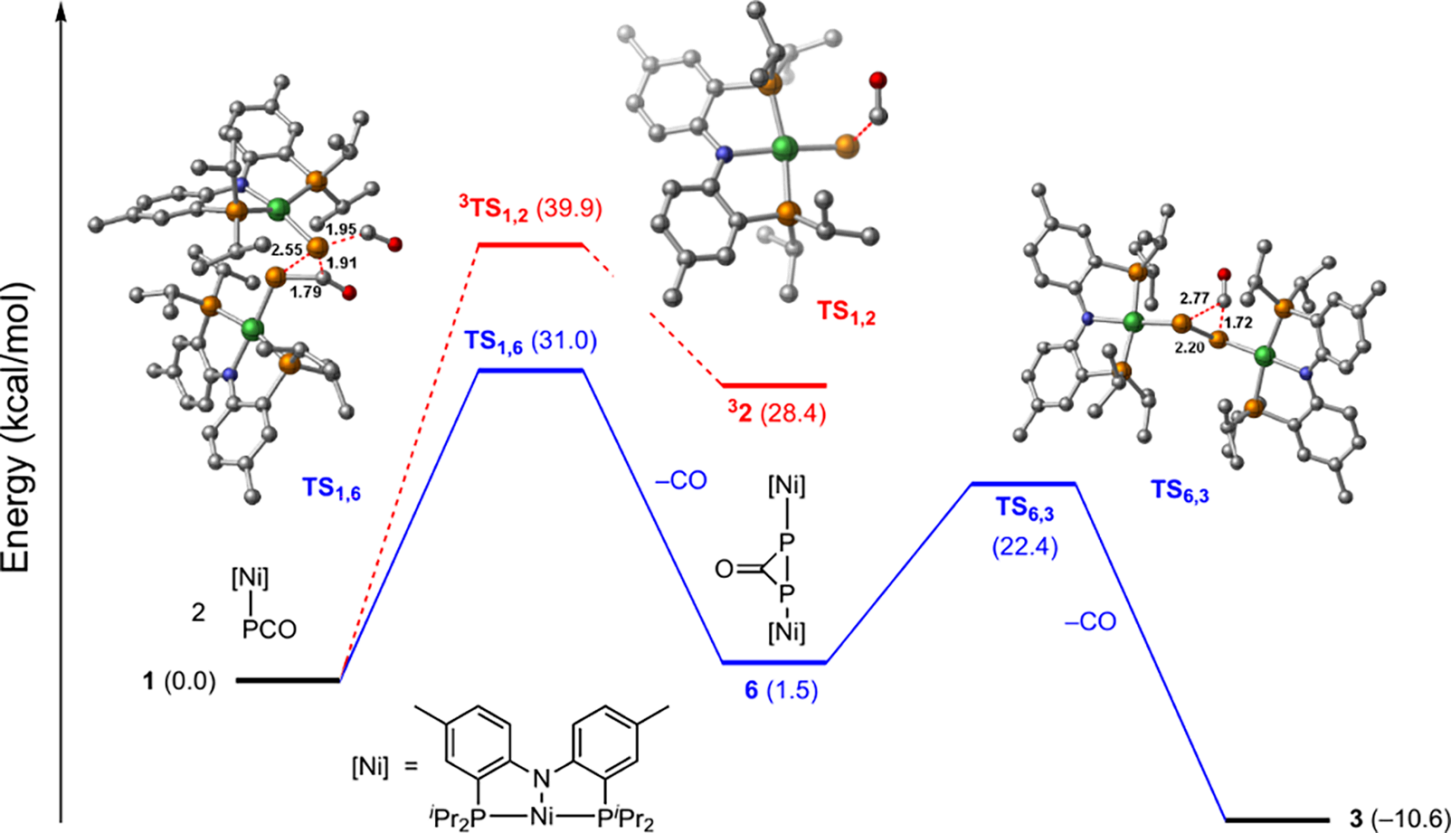
Computed potential energy surfaces for available decarbonylation and dimerization pathways from **1** to **3**. Calculations performed at the UB3LYP-D3/6-311+g(d,p)-CPCM(benzene)//UB3LYP-D3/def2svp-CPCM(benzene) level of theory. All structures illustrated are singlets unless otherwise specified; energy values are Δ*G* (kcal/mol) at 298 K.

**Figure 8:**
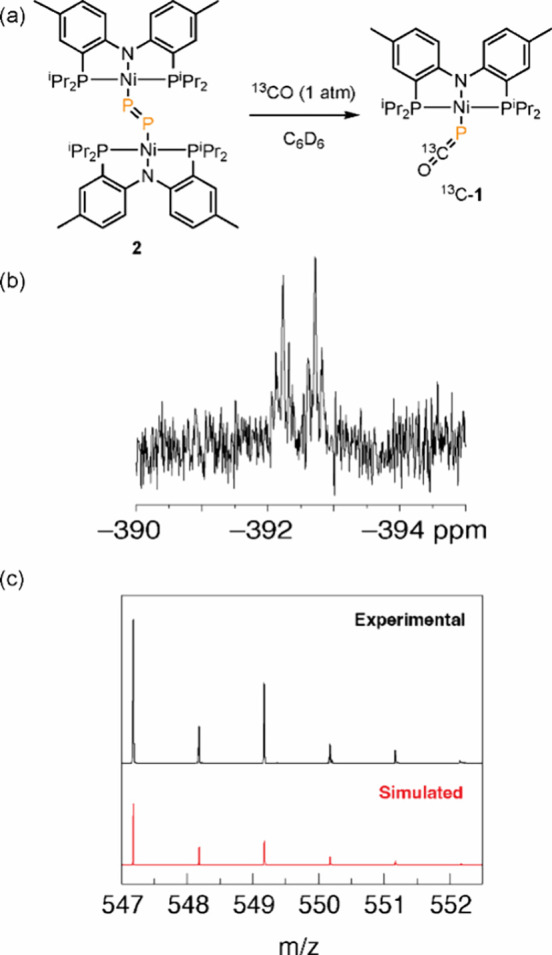
(a) Treatment of **3** with ^13^C–CO afforded ^13^C-**1**. (b) ^31^P{^1^H} of ^13^C**-1** highlighting the ^13^C–^31^P coupling (^1^J_CP_ = 99 Hz). (c) HR-MS of ^**13**^**C-1**.

**Figure 9:**
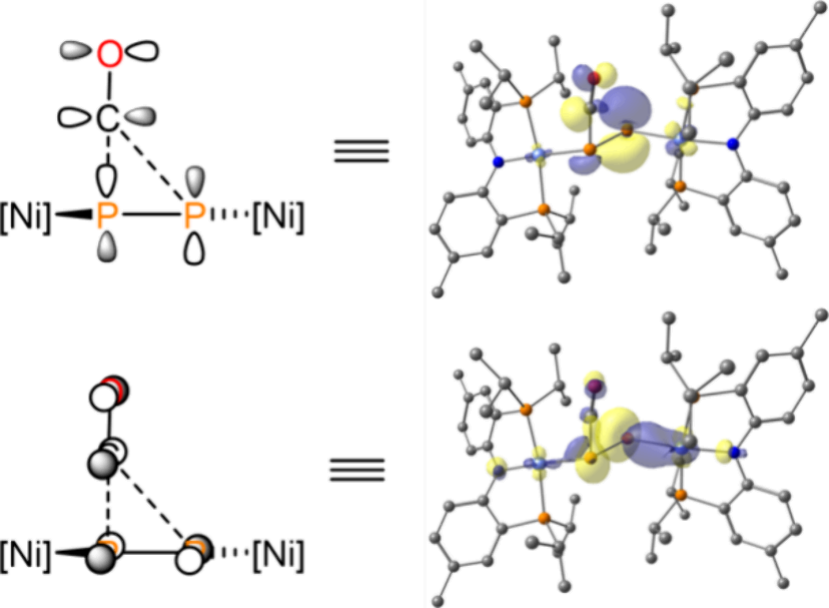
Orbital interactions between CO and the P_2_^2–^ ligand in TS_6,3_.

Despite confronting a prohibitive barrier for thermal CO extrusion from **1** (>40 kcal/mol), facile P–P coupling is also observed in the absence of light. Analysis of the reaction kinetics for thermal P–P coupling revealed a second-order reaction and activation parameters consistent with dimerization of **1** (Figure 6). A [2 + 1] transition state (i.e., TS_1,6_) is on-path to P–P coupling via the intermediacy of dinickel complex **6**, in which the Ni ions are bridged by a three-membered P_2_C ring (Figure 7). Consistent with facile thermal P–P coupling, P_2_-bridged complex **3** reacts with CO (or ^13^CO) to regenerate **1** (or ^13^C-**1**, Figure 8). Examination of the transition state for CO addition to the P_2_ fragment (i.e., TS_6,3_) shows that a side-on approach of CO to the P_2_ unit gives rise to a three-membered ring intermediate through concerted formation of two P–C bonds (Figure 9). Given that such (nearly) coplanar, zigzag motifs are common in diphosphorus compounds derived from phosphaethynolates,^47−49^ investigation into the ubiquity of reversible CO elimination from such species should be considered.

The results presented here elucidate a more complex potential energy surface for M–PCO activation than has previously been appreciated. We demonstrate that canonical M–PCO decarbonylation can provide entry to a reactive terminal Ni–P complex and we validate the proposal that these (typically unobserved) species engage in P–P coupling reactions. In addition, we disclose a novel, thermal process for P–P coupling from M–PCO precursors that avoids M–P intermediates. This facile thermal process provides a reversible mechanism for decarbonylation and the reverse carbonylation of diphosphorus fragments. These results highlight that care should be taken when considering the formation of products that are ostensibly the result of highly reactive metal–ligand bonded species. Further, these studies provide new insights into the reaction intermediates and processes that confront application of M–P intermediates in prospective PAT chemistry and catalytic applications. Investigations of the reaction chemistry of complexes **1** and **2** toward substrate functionalization chemistry and catalysis are currently being pursued.

## Supporting Information Available

Experimental procedures, spectral data, Cartesian coordinates for the calculated structures (PDF)
