# Supplementary material for: P–P Coupling with and without Terminal Metal–Phosphorus Intermediates
Source: J Am Chem Soc. 2025 Jan 28;147(6):5350–9. doi: 10.1021/jacs.4c16833 (PMC11826899; doi:10.1021/jacs.4c16833)
Supplement: Supplementary file 1 — ja4c16833_si_001.pdf [file ja4c16833_si_001.pdf]

Supporting Information

P−P Coupling *with* and *without* Terminal Metal–Phosphorus Intermediates

Richard R. Thompson[1,2,*], Matthew T. Figgins[1], Duleeka C. Wannipurage[1], Angel Renteria-Gomez[1], Achyut Ranjan Gogoi[1], Joshua Telser[3], David L. Tierney[4], Marc C. Neben[5], Serhiy Demeshko[5], Osvaldo Gutierrez[1,*], and David C. Powers[1,*]

[1] *Department of Chemistry, Texas A&M University, College Station, Texas 77843, United States*

[2] *Department of Chemistry, University of Idaho, Moscow, Idaho 83844, United States*

[3] *Department of Biological, Physical and Chemical Science, Roosevelt University, Chicago, Illinois 60605, United States*

[4] *Department of Chemistry and Biochemistry, Miami University, Oxford, Ohio 45056, United States*

[5] *Institut für Anorganische Chemie, Georg-August-Universität, Tammannstrasse 4, 37077 Göttingen, Germany.*

Email: rthompson@uidaho.edu, og.labs@tamu.edu, powers@chem.tamu.edu

Table of Contents

A. General Considerations S3

B. Synthesis and Characterization S7

C. Reactivity Studies S12

D. Supporting Data S15

E. Reaction Kinetics Data S44

F. Crystallographic Data S48

G. Computational Data S53

H. References S96

A. General Considerations

SAFETY NOTE $NaPH_2$ and its hydrolysis product, PH_3, are highly pyrophoric and toxic. Appropriate caution, personal protective equipment, and engineering controls should be employed.

Materials and Methods All reactions were carried out under an inert atmosphere of N_2 using standard Schlenk-line or glovebox techniques unless otherwise stated. All the chemicals and solvents (ACS reagent grade) were used as received unless otherwise noted. 1,3-Cyclohexadiene and molecular sieves (4 Å, MS) were purchased from Sigma Aldrich. 2-MeTHF was purchased from Oakwood Chemical. N_2 and CO were purchased from Airgas. 1,3-Cyclohexadiene was dried according to literature methods[1] and subsequently degassed by three free-pump-thaw cycles. Anhydrous pentane and toluene were obtained from a drying column.[2] Anhydrous diethyl ether, tetrahydrofuran (THF), 2-methyltetrahydrofuran (2-MeTHF) and dimethoxyethane were distilled from a sodium ketyl stills. Hexamethyldisiloxane was distilled from sodium metal. All solvents were stored over 4 Å molecular sieves. NMR solvents were obtained from Cambridge Isotope Laboratories were degassed by three free-pump-thaw cycles and were stored over molecular sieves (3 Å) for 24 h prior to use. (PNP)Ni(Cl) (**4**),[3] $NaPCO{\cdot}(dioxane)_{2.5}$,[4] and $NaPH_2$[5] were synthesized according to literature methods (PNP = bis(2-(diisopropylphosphaneyl)-4-methyl-phenyl)amide).

Characterization Details NMR spectra were recorded on Bruker Avance NEO 400 and Avance NEO 500 NMR instruments operating at 400.1 and 500.2 MHz, respectively, for ^{1}H. The NMR spectra were referenced against residual proteo solvent signal: C_6D_3 (7.16 ppm, ^{1}H; 128.06 ^{13}C) and $CDCl_3$ (7.26 ppm, ^{1}H).[6] ^{1}H NMR data are reported as follows: chemical shift (δ, ppm), integration, multiplicity (s (singlet), d (doublet), t (triplet), m (multiplet), br (broad), coupling constant(s), proton identity. ^{31}P{^{1}H} and ^{31}P NMR spectra were referenced against an external PPh_3 standard at –6.00 ppm. Solution-phase UV-vis spectra were recorded on a Shimadzu UV-2501PC spectrophotometer. Solution-phase spectra were blanked against the appropriate solvent. IR spectra were recorded on a Shimadzu FTIR/IRAffinity-1 spectrometer, were blanked against air, and were determined as the average of 64 scans. IR data are reported in wavenumber (cm^{-1}). Raman measurements were performed at room temperature using a Jobin Yvon Horiba LabRAM HR instrument coupled to an Olympus BX41 microscope using a 785 nm laser excitation. An 1800 lines/mm grating (450-850 nm) spectrometer equipped with a Peltier-cooled charge-coupled device (CCD) detector (Andor) was used. Hole and slit diameters were both 100 μm. Electrospray mass spectrometry data was recorded on either Orbitrap Fusion™

Tribrid™ Mass Spectrometer or Q Exactive™ Focus Hybrid Quadrupole – Orbitrap™ Mass Spectrometer from Thermo Fisher Scientific. Atmospheric pressure chemical ionization mass spectrometry (APCI-MS) experiment was performed using a Thermo Scientific Q Exactive Focus. Samples were injected into a 10 μL loop and methanol was used as a mobile phase at a flow rate of 500 μL/min. The Q Exactive Focus APCI source was operated in full MS in positive mode. The mass resolution was tuned to 70000 FWHM at m/z = 200. The discharge current was set at 5 μA, the sheath gas and auxiliary gas flow rates were set to 25 and 5 arbitrary units, respectively, and the auxiliary gas temperature was set to 300 °C. The transfer capillary temperature was held at 250 °C and the S-Lens RF level was set at 50 V. Exactive Series 2.11/Xcalibur 4.02.47 software was used for data acquisition and processing. EPR experiments were performed on a Bruker EMX spectrometer, equipped with an ER-4116 dual mode resonator. The perpendicular mode spectra were measured on freshly prepared samples that were photolyzed a few feet from the spectrometer and immediately transferred to the EPR cryostat. Spectra were acquired at 9.64 GHz (20 μW to 20 mW), using 10 G field modulation (100 kHz) and time constant/conversion time = 82 ms; a minimum of two scans were averaged for any setting.

Photochemistry Details *Steady-State Photolysis.* For standard photochemical reactions, a J-young/Schlenk tube was charged with compound **1** (1 equiv.), the appropriate substrate (if used, 100 equiv.), and C_6D_6. The solution was photolyzed by a Nikon Hg 100 W lamp equipped with a glass filter (λ > 335 nm) for 6 h at 23 °C. The reaction mixture was subsequently filtered through Celite and the products were characterized by $^{31}P\{^1H\}$ NMR spectroscopy.

Cryogenic Photolysis. An EPR tube fitted with a J-young cap was charged with compound **1**, 2-MeTHF and submerged in a liquid-N_2 cooled (77K) quartz cold finger dewar. The solution was then photolyzed by a Nikon Hg 100 W lamp equipped with a glass filter (λ > 335 nm) for approximately 4 h. The photolysis was monitored by *in situ* UV-vis spectrophotometry using an Ocean Optics USB2000+XR1-ES spectrometer and DH-2000-BAL UV-vis-NIR light source.

EPR studies. A sample of photolyzed **1** was prepared as described above. The entire magnetic field range (0.01 – 1.45 T) of the spectrometer, corresponding to g-values as low as 0.47, was investigated. Very weak signals (< 5 % of the total Ni present) were observed near 0.338 T (g ~2) that are attributed to radical impurities and/or decomposition products. No signals were observed above 0.4 T. That no signals from **2** were observed below 1.45 T indicates that the magnitude of zero-field splitting must be $|D| > 5.6\ cm^{-1}$. EPR simulations used the program SPIN by A. Ozarowski (National High Magnetic Field Laboratory, Florida State University), which employs a standard spin Hamiltonian for S = 1, allowing for g anisotropy (although $g_{iso} = 2.0$ was used for the simulations) and axial and rhombic zero-field splitting.[7, 8]

Photomagnetism Experiments. Magnetic measurements on **2** were carried out using a *Quantum Design* MPMS3 SQUID magnetometer. The photoproduct **2** was formed *in situ* via irradiation of **1** with a TLS120Xe xenon light source, using the fiber optical sample holder (FOSH) to allow optical access to the sample. For this, **1** was placed inside the FOSH and inserted into the magnetometer. The sample was centered in the magnetometer coils and cooled down to 2 K. To obtain a background measurement as a raw SQUID voltage response function of the set-up before the reaction, the magnetic moment of **1** in the FOSH was measured from 2 K to 295 K at a magnetic field of 5000 Oe. Afterwards, the sample was cooled back down to 10 K and reaction from **1** to **2** was carried out by photolysis at 350 nm over 140 min. The reaction was monitored by measuring the DC moment of the sample over the whole period of irradiation at a magnetic field of 5000 Oe (Figure S9). This reaction was followed by a temperature-dependent measurement of the reaction product **2** from 2 K to 295 K at a magnetic field of 5000 Oe. The signal that can be attributed to **2** was extracted from the data by subtracting the raw response function of the measured background from the raw response function of the reaction product[9] using the *mpView.1.4.1* program.[10] From the resulting magnetic moment, the molar susceptibility of **3** was calculated. As the photolysis yield in **3** is not known, the amount of **3** was varied until the measured $\chi_M T$ product reached the theoretical value for an $S = 1$ system with $g = 2.07$, namely $\chi_M T = 1\ \mathrm{cm^3 mol^{-1} K}$. From this result, formation of 0.005 mg of **3** was calculated. The data was fitted with a spin Hamiltonian approach, using the *julX* program according to $\hat{H} = g\mu_B \vec{B}\vec{S} + D\left[\hat{S}_z^2 - \frac{1}{3}S(S+1)\right]$.[11] Only the lower temperature data were fitted ($T \leq 20$ K) because photoproduct **3** is unstable at higher temperatures.

X-ray Diffraction Details Experimental details of crystallization are included in the synthetic procedures for the relevant compounds and crystallographic data are summarized in Tables S1 and S2. Suitable crystals for X-ray analysis of compounds **1**, **3**, **5**, **7**, and **8** were placed on a MiTeGen pin, coated in oil, and placed under a cold N_2 stream (Oxford). The X-ray intensity data collection was carried out on a Bruker PHOTON III CCD area detector using graphite-monochromated Mo-Kα radiation (λ = 0.71072 Å) at 90(5) K. Preliminary indexing was performed from a series of thirty-six 0.5° rotation frames with exposures of 10 s. Rotation frames were integrated using SAINT[2] producing a listing of non-averaged F^2 and $\sigma(F^2)$ values. The intensity data were corrected for Lorentz and polarization effects and for absorption using SADABS.[3] The initial structure was determined by the direct method on SHELXS.[4] The further structure determination was performed by Fourier transform method and refined by least squares method on SHELXL.[5,6] All reflections were used during refinement. Non-hydrogen atoms were refined anisotropically and hydrogen atoms were refined with riding models, unless otherwise mentioned. Disordered isopropyl groups and solvents were successfully modeled.

Computational Details All geometry optimizations of intermediates and transition states were achieved using spin-unrestricted UB3LYP[12]-D3[13]/def2-SVP[14]method, in dichloroethane solvent using the CPCM solvent model[15] with “opt=noeigen” and “guess=mix” keywords as implemented in Gaussian16.[16] Frequency calculations were also conducted at the same level of theory to obtain vibrational frequencies to determine the identity of stationary points as intermediates (no imaginary frequencies) or transition states (only one imaginary frequency), as well as obtaining the thermochemistry: enthalpy (ΔH) and free energy (ΔG) at the temperature of 298 K. Single point calculations are carried out in different higher level methods to ensure consistency independent of methods used. The methods used for the purpose are UB3LYP-D3/def2TZVPP-CPCM(benzene)//UB3LYP-D3/def2svp-CPCM(benzene) and UB3LYP-D3/6-311+g(d,p)-CPCM(benzene)//UB3LYP-D3/def2svp-CPCM(benzene). UV–Vis absorption spectra were simulated using TD-DFT[17] single points at the B3LYP-D3BJ/Def2-TZVP(SMD-Benzene) level of theory on the B3LYP-D3BJ/def2-TZVP optimized geometries. The first 30 vertical excitations were solved iteratively. The simulated spectra were generated using an in-house coded Fortran program.[18] The nature of the transition was shown analyzing the Natural Transition Orbitals, and the NTO images were generated using GaussView6.[19] All structural figures were generated with CYLview.[20] Also, to investigate the bonding in **2**, Natural Bonding Orbitals (NBO, version 3.1)[21] approaches were employed. Also was employed domain based local pair natural orbital coupled cluster method with single-, double-, and perturbative triple excitations and def2-SVP basis set along with def2-SVP/C as auxiliary basis set [noted as DLPNO-CCSD(T)/def2-SVP-CPCM(Benzene)//(U)B3LYP-D3/def2-SVP-CPCM(Benzene)] with ORCA (version 4.1.1).[22] Distances in structural figures are shown in Å and energies are in kcal/mol.

B. Synthesis and Characterization

Synthesis of (PNP)Ni(PCO) (1)

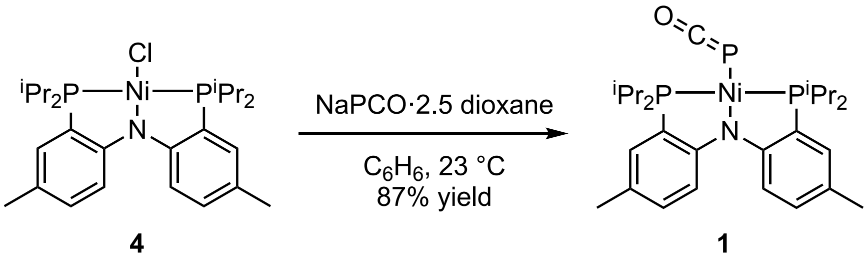

A 20-mL scintillation vial was charged with **4** (272 mg, 0.520 mmol, 1.00 equiv) and benzene (5 mL). Separately, a 20-mL scintillation vial was charged with NaPCO·(dioxane)$_{2.5}$ (165 mg, 0.572 mmol, 1.10 equiv) and benzene (5 mL). With the exclusion of ambient light, the NaPCO suspension was added to the (PNP)NiCl solution. The reaction mixture was stirred for 3 min, after which time a dark blue-green solution was observed. The reaction was filtered through a Celite plug to remove undissolved solids and solvent was removed under reduced pressure. The product was dissolved into minimal toluene. Storage overnight at –35 °C provided dark green plates of the title compound suitable for X-ray diffraction. Yield = 274 mg (87% yield). ^{1}H NMR (400 MHz, C_6D_6, 25 °C): 1.12 (12H, dd, $^{3}J_{HH}$ = 7.5 Hz, $^{3}J_{HH}$ = 8.0 Hz CH(C*H*$_3$)$_2$), 1.48 (12H, dd, $^{3}J_{PH}$ = 7.5 Hz, $^{3}J_{HH}$ = 8.0 Hz CH(C*H*$_3$)$_2$), 2.13 (6H, s, *p*-C*H*$_3$), 2.35 (4H, m, $^{3}J_{HH}$ = 3.4 Hz, C*H*(CH$_3$)$_2$), 6.76 (2H, d, $^{3}J_{HH}$ = 8.5 Hz, Ar–*H*), 6.86 (2H, dd, $^{3}J_{HH}$ = 8.5 Hz, $^{4}J_{HH}$ = 1.8 Hz, Ar–*H*), 7.58 (2H, dd, $^{3}J_{HH}$ = 8.5 Hz, $^{4}J_{HH}$ = 1.8 Hz, Ar–*H*). ^{13}C{^{1}H} NMR (125 MHz, *C_6D_6*, 25 °C): 162.3 (dt, $^{1}J_{PC}$ = 99 Hz, $^{3}J_{PC}$ = 5 Hz, P*C*O), 161.5 (t, $^{3}J_{PC}$ = 12.5 Hz, *Ar*), 132.7 (*Ar*), 131.9 (*Ar*), 125.4 (*Ar*), 120.8 (*Ar*), 115.8 (*Ar*), 24.7 (t, $^{2}J_{PC}$ = 12.7 Hz, *C*H(CH$_3$)$_2$), 20.5 (*p*-*C*H$_3$), 18.8 (CH(*C*H$_3$)$_2$), 17.7 (CH(*C*H$_3$)$_2$).^{31}P{^{1}H} NMR (162 MHz, C_6D_6, 25 °C): –393 (t, $^{2}J_{PP}$ = 20 Hz, *P*CO), 38 (d, $^{2}J_{PP}$ = 20 Hz, *P*^{i}Pr$_2$). FT-IR (KBr, cm^{-1}): 1869 (PCO). UV-vis (toluene), λ_{max} (nm, ε (M^{-1}cm^{-1})): 323 (5.9 × 10^{3}), 349 (6.5 × 10^{3}), 577 (1.7 × 10^{2}). HR ESI-MS (m/z): calcd. for $C_{27}H_{41}NNiP_3O^+$, [M + H]$^+$, 546.1749, found, 546.1735.

Synthesis of $[(PNP)Ni]_2(P_2)$ (3)

Thermal conditions

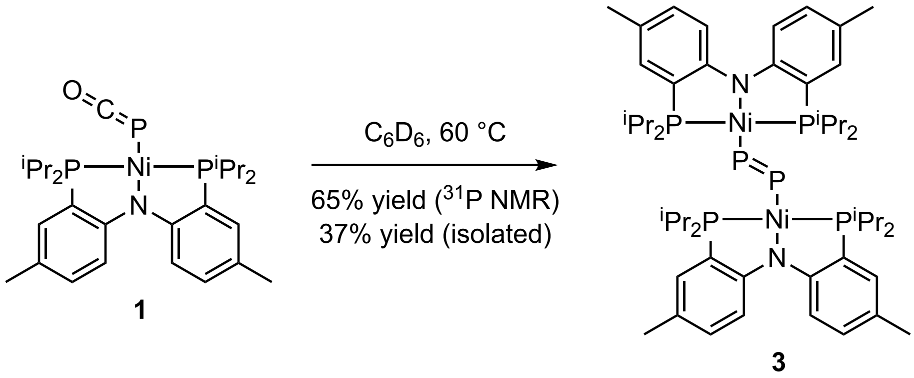

A 20-mL scintillation vial was charged with compound **1** (22 mg, 0.042 mmol, 1.0 equiv) and C_6D_6 (0.5 mL). The solution was transferred to a J. Young NMR tube, and freeze-pump-thawed four times. The sample was then heated to 60 °C and kept at that temperature for 6 h. During this time, the dark blue-green solution became dark brown-purple. Conversion was assayed by integration of the $^{31}P\{^1H\}$ NMR spectrum against an internal standard of PPh_3 (65% spectroscopic yield.). Solvent was removed under reduced pressure. The product extracted into toluene (0.2 mL) and the resulting solution was stored at 23 °C. Large, dark brown blocks suitable for X-ray diffraction crystallized out of solution over the course of 1 week. Yield = 8 mg (37% isolated yield). ^1H NMR (400 MHz, C_6D_6, 25 °C): 1.33–1.41 (24H, m, $CH(CH_3)_2$), 2.22 (6H, s, *p*-CH_3), 2.31 (4H, m, $^3J_{HH}$ = 3.2 Hz, $CH(CH_3)_2$), 6.88 (2H, d, $^3J_{HH}$ = 8.5 Hz, Ar–*H*), 7.11 (2H, s, Ar–*H*), 7.78 (2H, d, $^3J_{HH}$ = 8.5 Hz, Ar–*H*). $^{13}C\{^1H\}$ NMR (125 MHz, *C_6D_6*, 25 °C): 161.3 (*Ar*), 132.6 (*Ar*), 132.2 (*Ar*), 123.9 (*Ar*), 120.7 (*Ar*), 115.7 (*Ar*), 24.2 ($CH(CH_3)_2$), 20.7 (*p*-CH_3), 19.1 ($CH(CH_3)_2$), 18.5 ($CH(CH_3)_2$). $^{31}P\{^1H\}$ NMR (162 MHz, C_6D_6, 25 °C): 30 (s, P^iPr_2), 762 (br s, $\Delta\nu_{1/2}$ = 29 Hz, *P_2*). Raman (cm^{-1}): 577 (P_2). UV-vis (C_6H_6), λ_{max} (nm, ε ($M^{-1}cm^{-1}$)): 354 (8.1×10^3), 523 (8.3×10^2).

Photochemical conditions

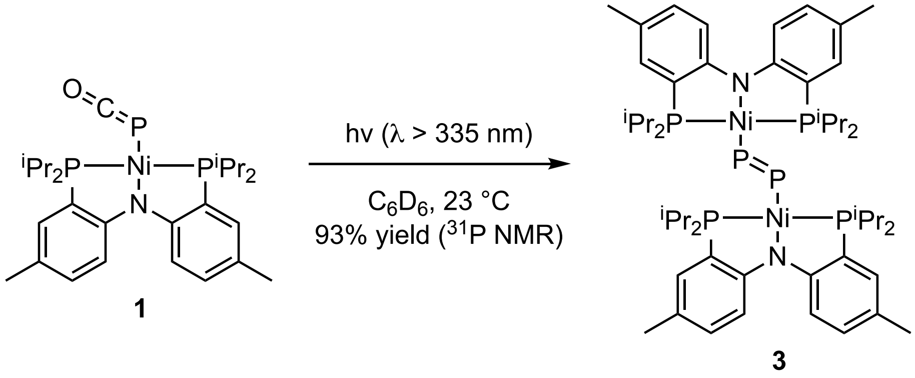

A J. Young NMR tube was charged with 0.5 ml of C_6D_6 solution of compound **1** (11 mg, 0.019 mmol). The sample was freeze-pump-thawed four times to degas and evacuate the headspace of the tube and then photolyzed for 6 h at 23 °C. The dark blue solution became dark purple-brown. Conversion was assayed by integration of the resulting $^{31}P\{^{1}H\}$ NMR spectrum against an internal standard of PPh_3 (93% spectroscopic yield). Spectral features of compound **3** produced photochemically were indifferentiable from those reported above for thermally generated compound **3**.

Synthesis of (PNP)Ni(PH_2) (5)

Salt metathesis conditions

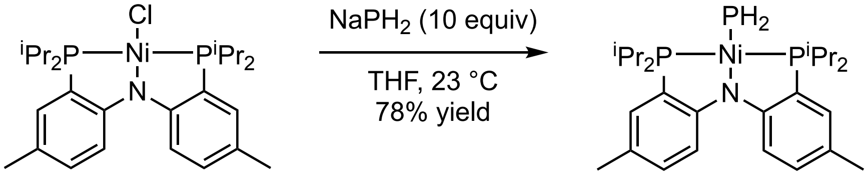

4 **5**

A 100-mL round-bottom flask was charged with **5** (65 mg, 0.12 mmol, 1.0 equiv) and THF (15 mL). Separately, a 20-mL scintillation vial was charged with $NaPH_2$ (70 mg, 1.2 mmol, 10 equiv) and THF (10 mL). With stirring, the $NaPH_2$ solution was added to the (PNP)NiCl solution. The reaction was stirred at 23 °C for 3 h at which time a dark red solution was observed. Solvent was removed under reduced pressure. The residue was suspended in pentane (~10 mL) and solids were removed by filtration. Solvent was removed under reduced pressure and the product dissolved into minimal toluene. Storage overnight at –35 °C gave dark red plates of product suitable for X-ray diffraction. Bulk material could be isolated by recrystallization from minimal pentane at –35 °C overnight. Yield = 50 mg (78% yield). ^{1}H NMR (400 MHz, C_6D_6, 25 °C): 1.14 (12H, d, $^3J_{HH}$ = 6.8 Hz, CH*(CH*$_3$)$_2$), 1.31 (12H, d, $^3J_{HH}$ = 7.6 Hz CH*(CH*$_3$)$_2$), 1.43 (2H, dt, $^1J_{PH}$ = 170 Hz, $^3J_{PH}$ = 14.8 Hz, P*H$_2$*) 2.18 (6H, s, *p*-C*H*$_3$), 2.22 (4H, m, $^3J_{HH}$ = 3.2 Hz, C*H(*CH$_3$)$_2$), 6.83 (2H, d, $^3J_{HH}$ = 8.8 Hz, Ar–*H*), 6.96 (2H, t, $^3J_{HH}$ = 4.4 Hz, Ar–*H*), 7.72 (2H, d, $^3J_{HH}$ = 8.4 Hz, Ar–*H*). ^{13}C{^{1}H} NMR (100 MHz, *C_6D_6*, 25 °C): 161.4 (t, J = 12.3 Hz, *Ar*), 131.9 (t, J = 5.7 Hz, *Ar*), 124.3 (t, J = 3.1 Hz, *Ar*), 121.3 (d, J = 3.2 Hz, *Ar*), 25.0 (t, J = 12.7 Hz, *C*(CH$_3$)$_2$), 20.6 (*p*-*C*H$_3$), 19.0 (m, J = 2.1 Hz, C(*C*H$_3$)$_2$, 18.1 C(*C*H$_3$)$_2$. ^{31}P{^{1}H} NMR (162 MHz, C_6D_6, 25 °C): –173.2 (t, $^2J_{PP}$ = 23 Hz, *P*H$_2$), 41.7 (d, $^2J_{PP}$ = 23 Hz, *P*^{i}Pr$_2$). ^{31}P NMR (162 MHz, C_6D_6, 25 °C): –173 (tt, $^1J_{PH}$ = 171 Hz, $^2J_{PP}$ = 23 Hz, *P*H$_2$), 42 (d, $^2J_{PP}$ = 23 Hz, *P*^{i}Pr$_2$). FT-IR (KBr, cm^{-1}): 2289 (PH_2), 2253 (PH_2). UV-vis (toluene), λ_{max} (nm, ε ($M^{-1}cm^{-1}$)): 314 (2.4×10^4), 349 (3.2×10^4), 521 (1.3×10^2).

Photochemical conditions

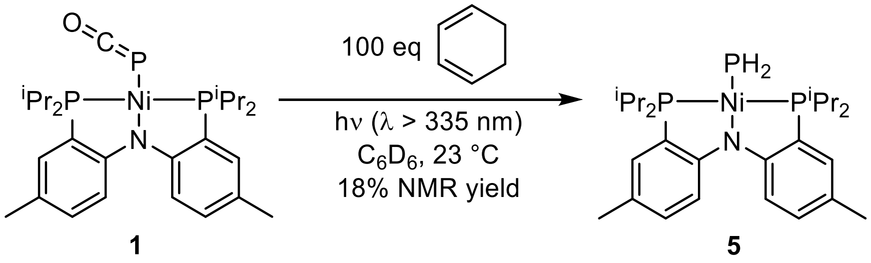

A 20-mL scintillation vial was charged with compound **1** (10 mg, 0.019 mmol, 1.0 equiv), 1,3-cyclohexadiene (0.18 ml, 0.19 mmol, 10 equiv.), and C_6D_6 (1.0 mL). The solution was transferred to a J. Young NMR tube and then photolyzed for 6 h at 23 °C. The dark blue solution became dark red-brown. The $^{31}P\{^1H\}$ spectrum of resulting solution evidenced a mixture of compounds **3**, **5**, and **8** as well as unidentified products. Spectral features of compound **5** produced photochemically were indifferentiable from those reported above for compound **5** generated via salt metathesis.

C. Reactivity Studies

Photoactivation of 3 and Reaction with *in situ* Generated CO

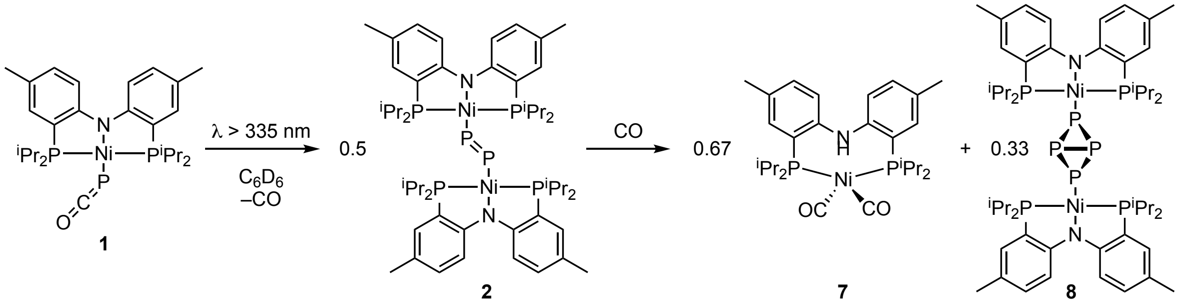

This experiment was carried out to investigate the photoreaction of **3** in the presence of the CO generated by photoactivation of **1**. A 20-mL scintillation vial was charged with compound **1** (60 mg, 0.11 mmol, 1.0 equiv) and C_6D_6 (0.6 mL). The solution was transferred to a J. Young NMR tube and then photolyzed for 6 h at 23 °C. The dark blue-green solution became dark brown. The $^{31}P\{^1H\}$ spectrum of resulting solution evidenced a mixture of compounds **3**, **7**, and **8** as the predominate products in an approximate 2:1:1 ratio. Solvent was removed under reduced pressure.

Compound **7**: $^{31}P\{^1H\}$ NMR (162 MHz, C_6D_6, 25 °C): 19 (s, P^iPr_2). FT-IR (KBr, cm^{-1}): 1992 (CO), 1932 (CO). Single crystals were grown from a concentrated pentane solution at –35 °C overnight.

Compound **8**: $^{31}P\{^1H\}$ NMR (162 MHz, C_6D_6, 25 °C): –311 (t, $^1J_{PP}$ = 112 Hz, P_4), –41 (t, $^1J_{PP}$ = 112 Hz, P_4), 36 (s, P^iPr_2). Single crystals were grown from a concentrated hexamethyldisiloxane solution at 23 °C over the course of 2 weeks.

Reaction of Compound 2 with 1 Atmosphere of $CO_{(g)}$

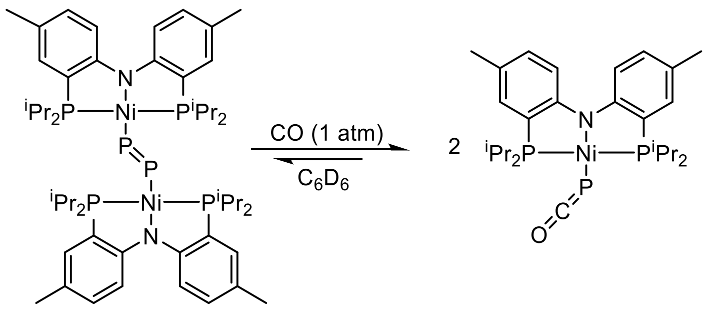

3 **1**

In a nitrogen-filled glovebox, a J-young NMR tube was charged with 0.5 mL of a 10 mM C_6D_6 solution of compound **3** (0.0048 mmol, 1.0 equiv). The headspace was evacuated via two freeze-pump-thaw cycles and the headspace was back-filled with 1 atm $CO_{(g)}$. The sample was warmed to 23 ºC at which temperature it was maintained for 16 h. The $^{31}P\{^1H\}$ NMR spectrum of the reaction mixture evidenced nearly full consumption of compound **3** as well as growth of signals associated with compounds **1**, **7**, and P_4 (–521 ppm).[23]

Reaction of Compound 2 with 1 Atmosphere of $^{13}CO_{(g)}$

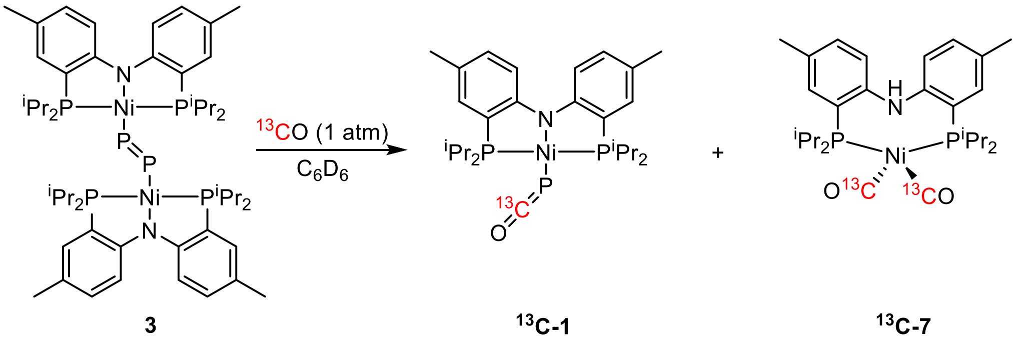

In a nitrogen-filled glovebox, a J-young NMR tube was charged with 0.5 ml of a 10 mM C_6D_6 solution of compound **3** (0.0048 mmol, 1.0 equiv). The headspace was evacuated via two freeze-pump-thaw cycles and the headspace was back-filled with 1 atm $^{13}CO_{(g)}$. The sample was warmed to 23 ºC at which temperature it was maintained for 16 h. The $^{31}P\{^1H\}$ NMR spectrum of the reaction mixture evidenced nearly full consumption of compound **3** as well as growth of signals associated with compounds ^{13}C-**1** and ^{13}C-**7**, as well as P_4 (–521 ppm).[23]

Compound ^{13}C-**1**: $^{31}P\{^1H\}$ NMR (202 MHz, C_6D_6, 25 °C): –392 (dt, $^1J_{CP}$ = 99 Hz, $^2J_{PP}$ = 20 Hz, *P*CO), 38 (dd, $^2J_{PP}$ = 20 Hz, $^3J_{CP}$ = 5 Hz, P^iPr_2). ATIR (cm^{-1}): 1823 (P^{13}CO). HR ESI-MS (m/z): calcd. for $C_{26}{}^{13}CH_{41}NNiP_3O^+$, $[M + H]^+$, 547.1783, found, 547.1775.

Compound ^{13}C-**7**: $^{31}P\{^1H\}$ NMR (202 MHz, C_6D_6, 25 °C): 19 (s, P^iPr_2). ATIR (cm^{-1}): 1866 cm^{-1} (^{13}CO), 1944 cm^{-1} (^{13}CO)

D. Supporting Data

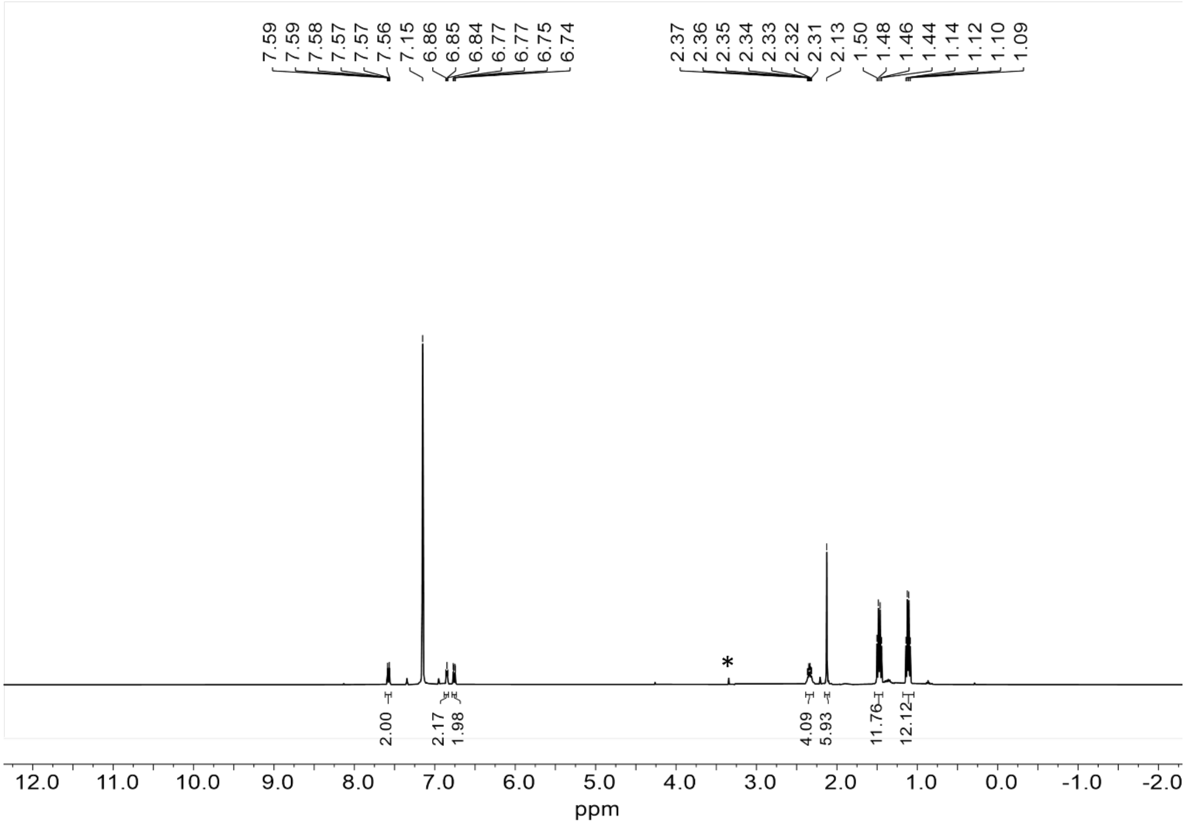

Figure S1. ^{1}H NMR (400 MHz, C_6D_6, 25 °C) spectrum of compound **1**. *Denotes residual 1,4-dioxane.

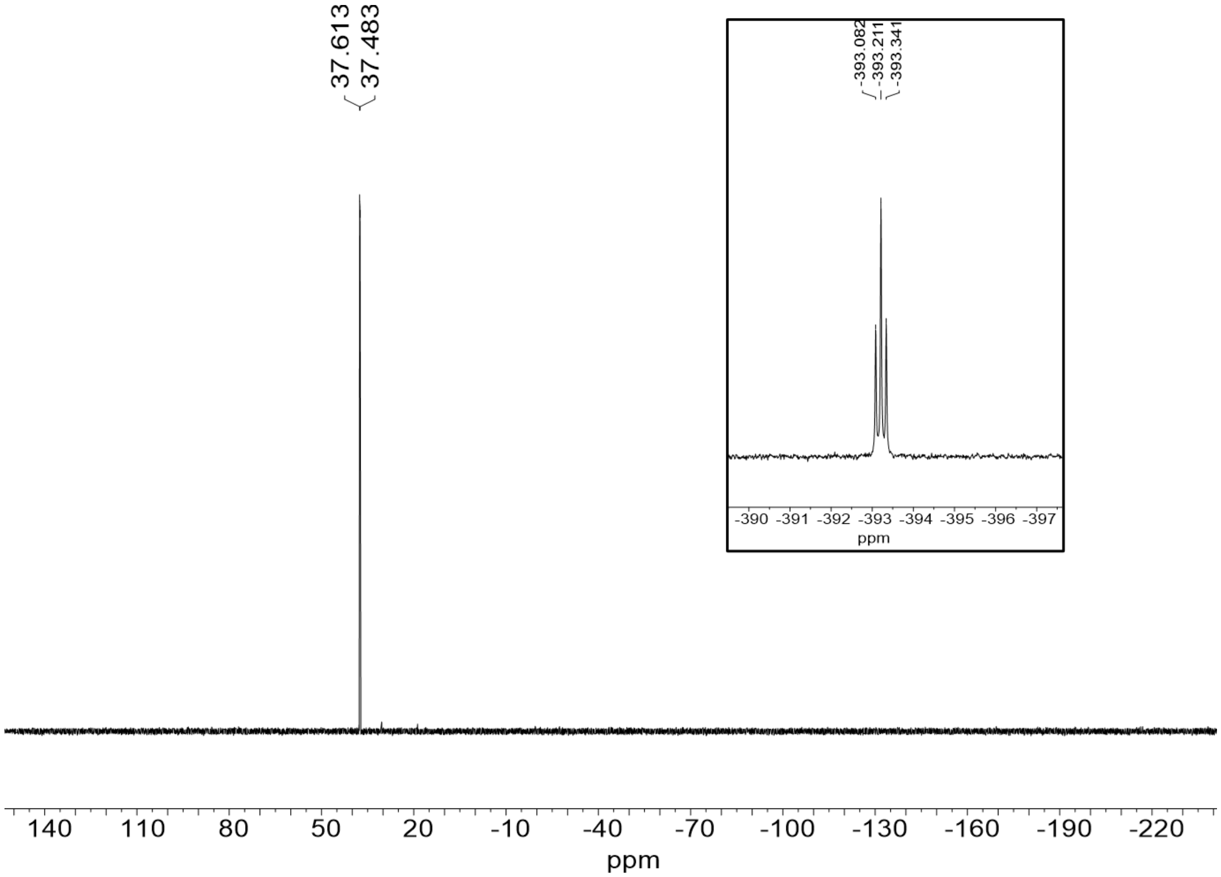

Figure S2. $^{31}P\{^1H\}$ NMR (162 MHz, C_6D_6, 25 °C) spectrum of compound **1**. Inset highlights the *P*CO signal at –393 ppm.

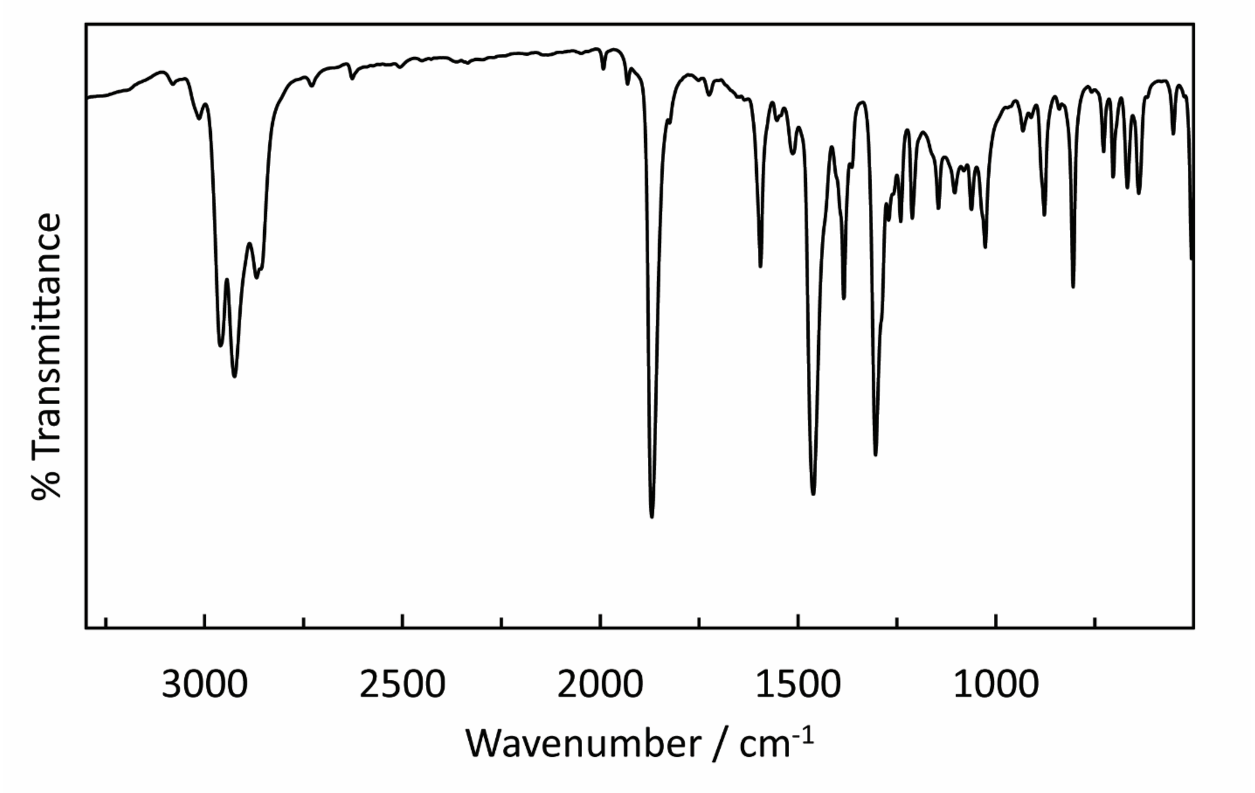

Figure S3. FT-IR spectrum (KBr) of compound **1**.

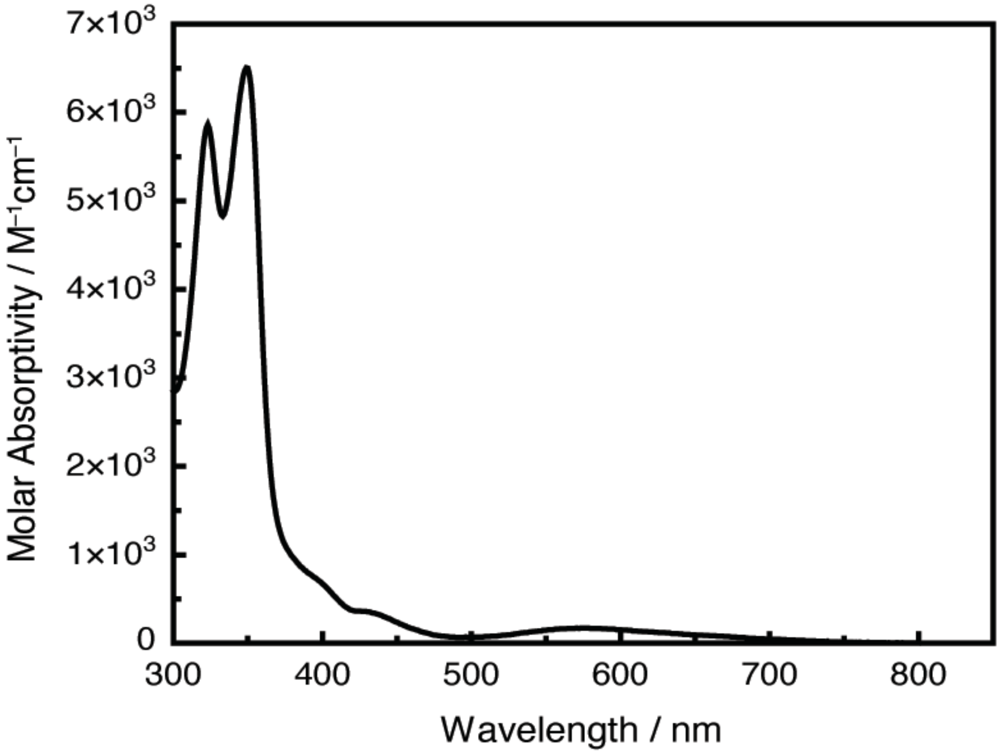

Figure S4. UV-vis absorptivity spectrum (C_6H_6, 25 °C) of compound **1**.

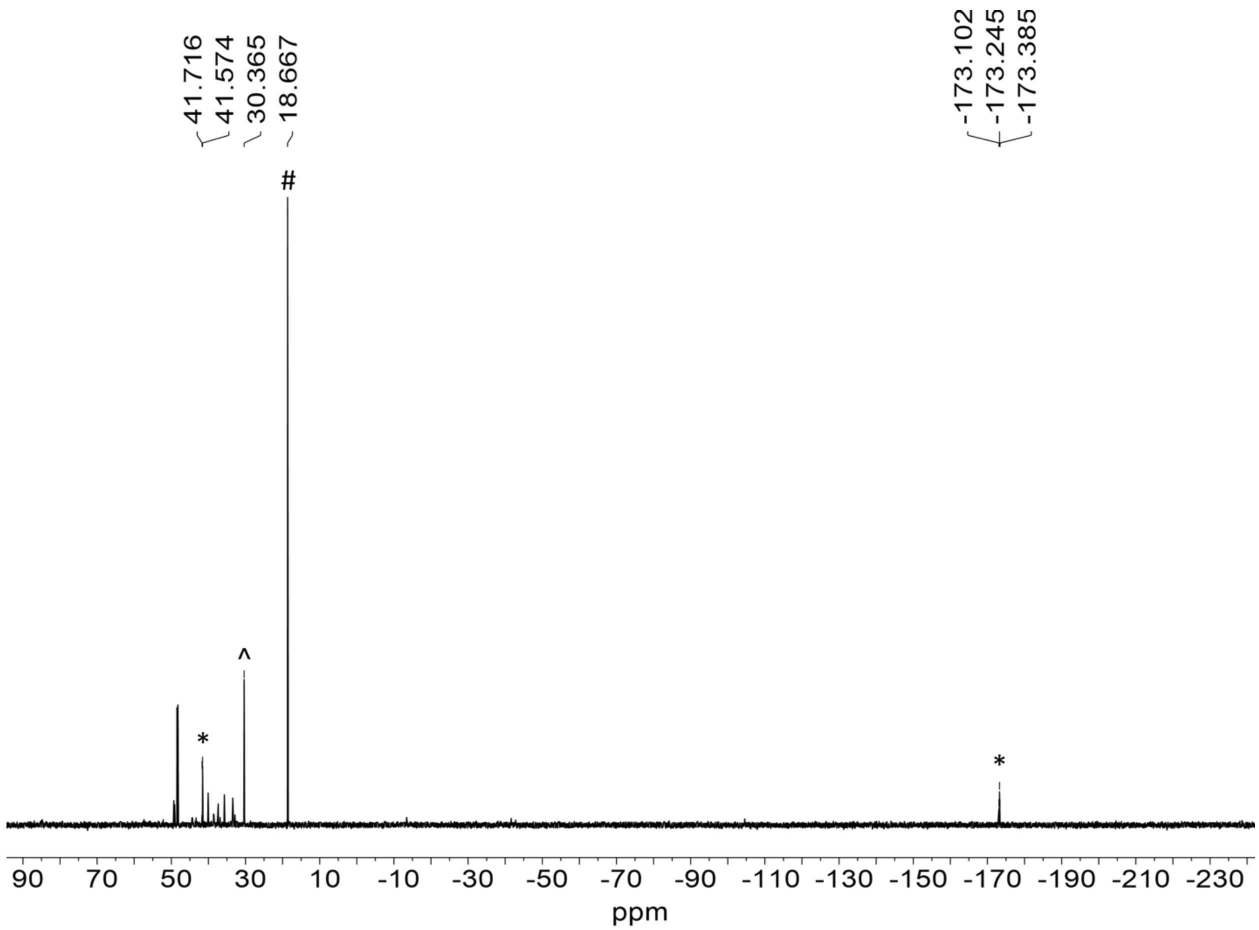

Figure S5. $^{31}P\{^1H\}$ NMR (162 MHz, C_6D_6, 25 °C) spectrum of the crude photolysis reaction mixture of compound **1** in the presence of 100 eq of 1,3-cyclohexadiene to form compounds **5** (*), **3** (^), and **7** (#).

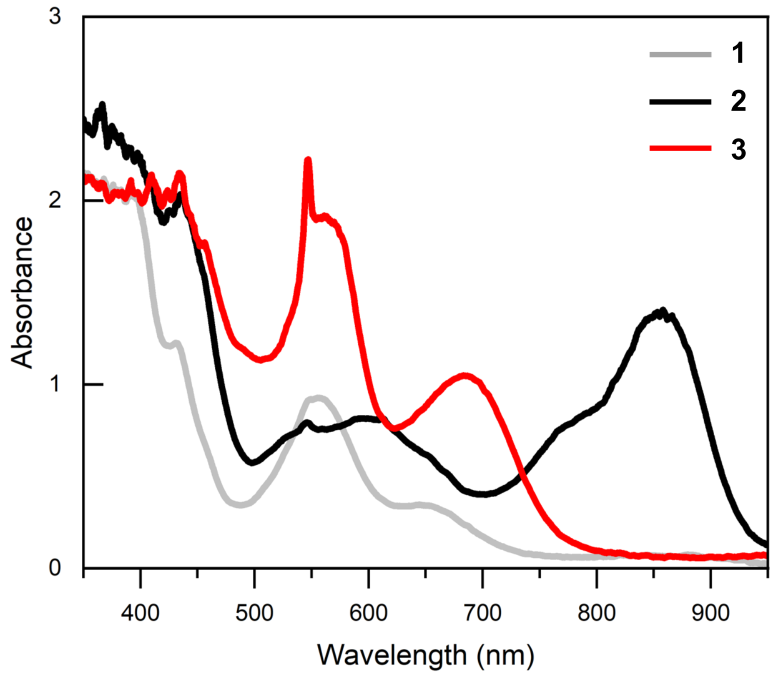

Figure S6. UV-vis spectra of 2-MeTHF solutions of compound **1** (grey, 77 K), **2** (black, 77 K), and **3** (red, 298 K).

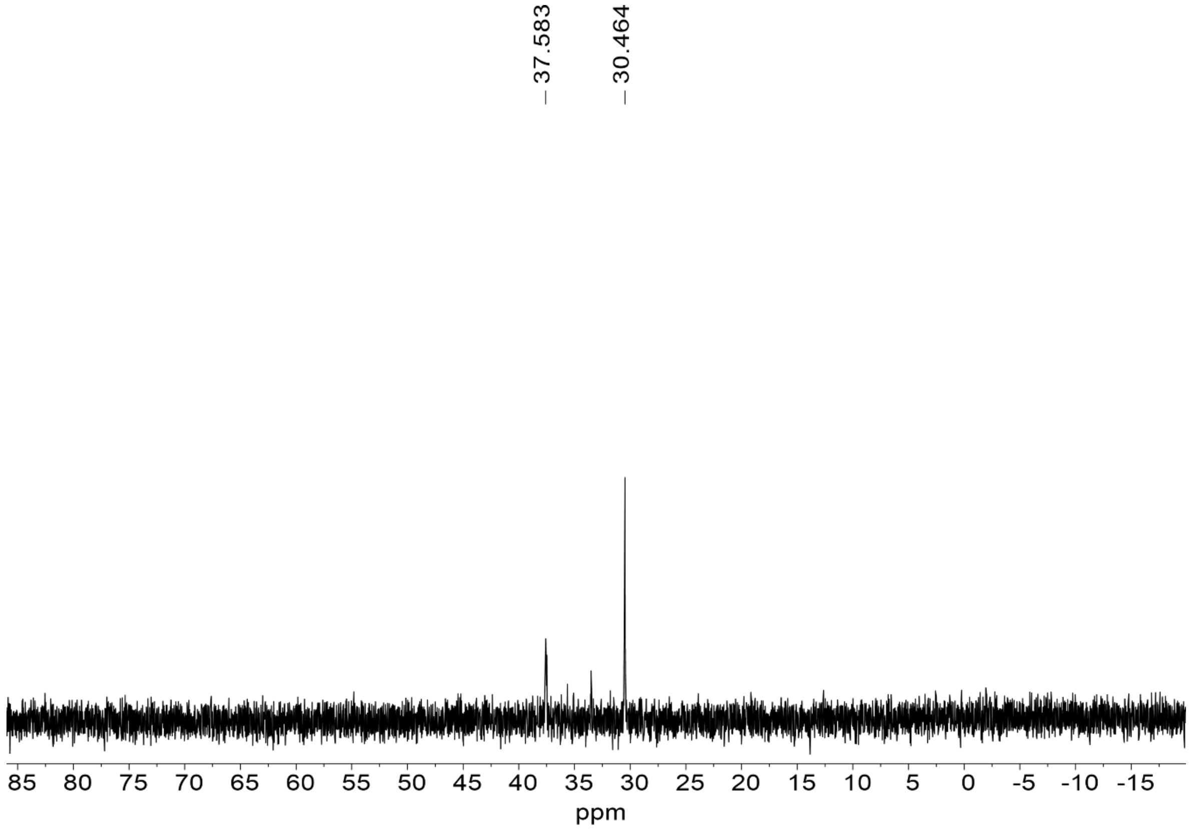

Figure S7. $^{31}P\{^{1}H\}$ NMR spectrum of **3**, generated by thawing a photolysis (@ 77 K) reaction mixture of **1**.

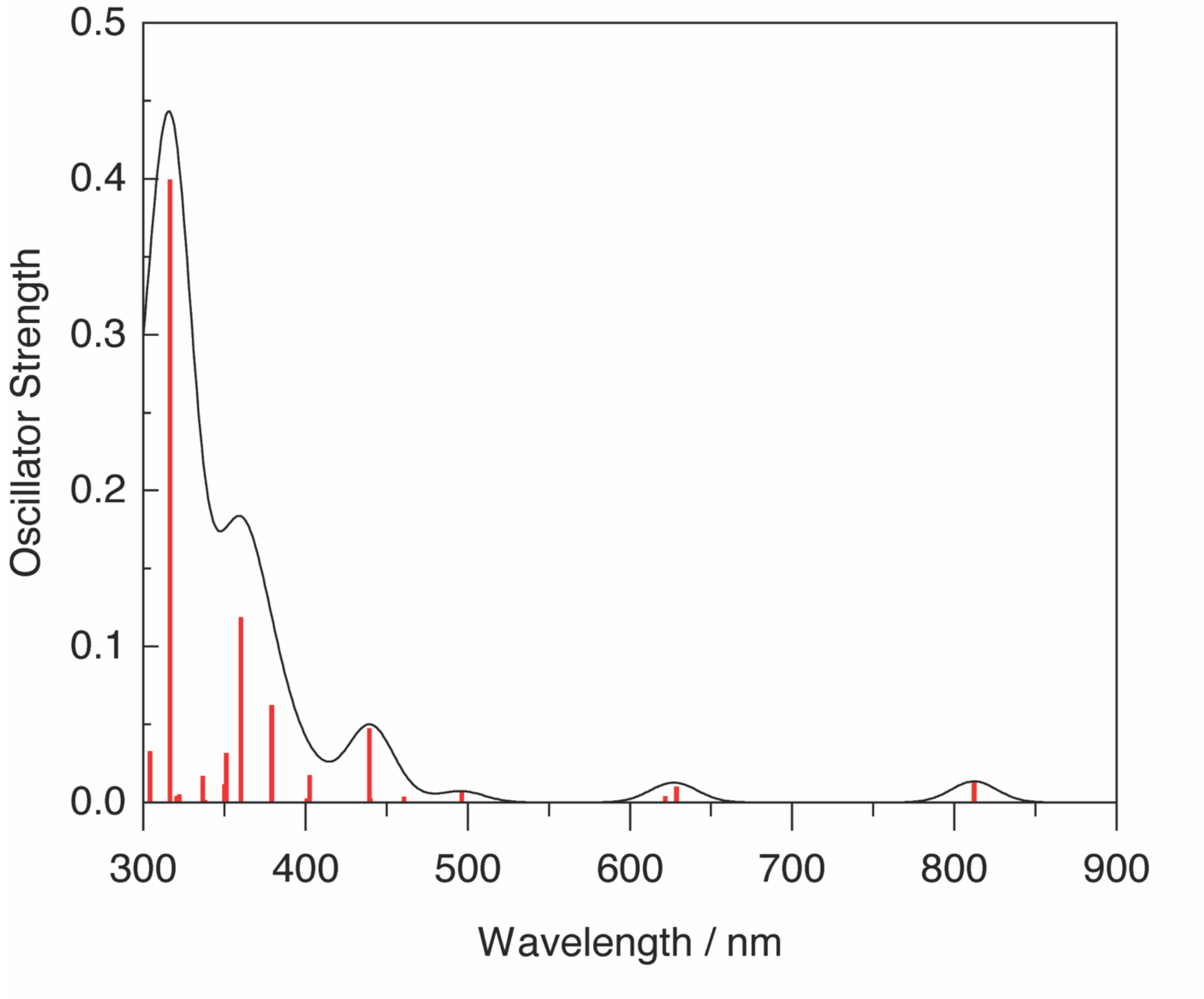

Figure S8. TD-DFT of singlet **2** (^{1}**2**).

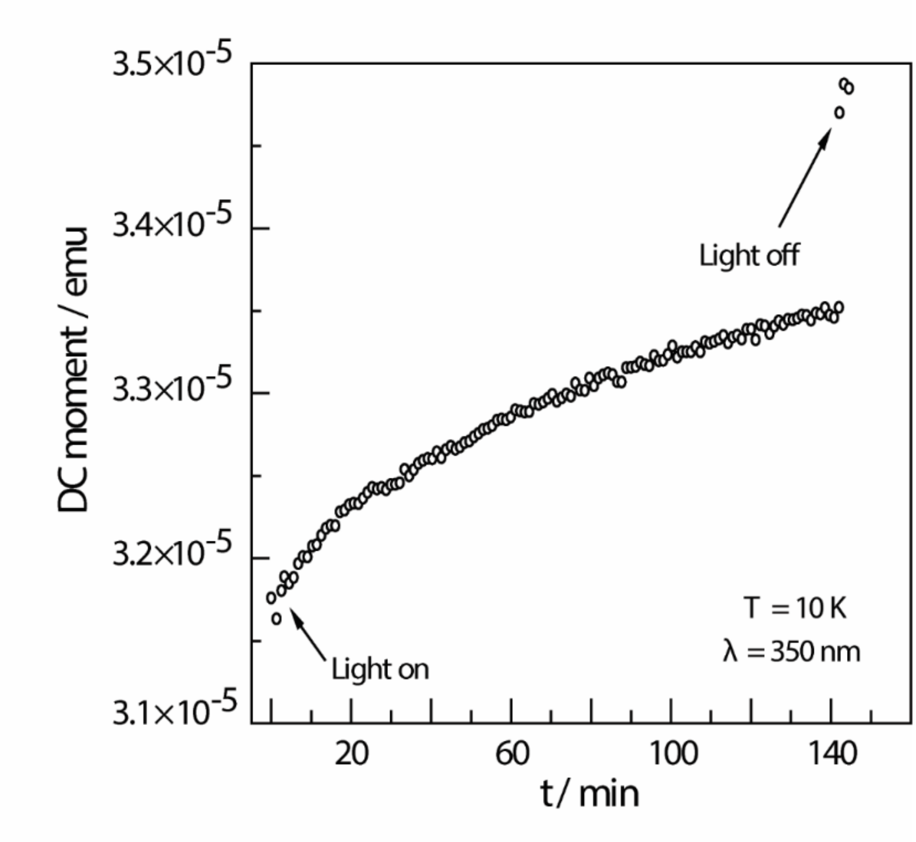

Figure S9. Increase of the DC moment after irradiation of **1** at 350 nm and 10 K under applied magnetic field of 5000 Oe.

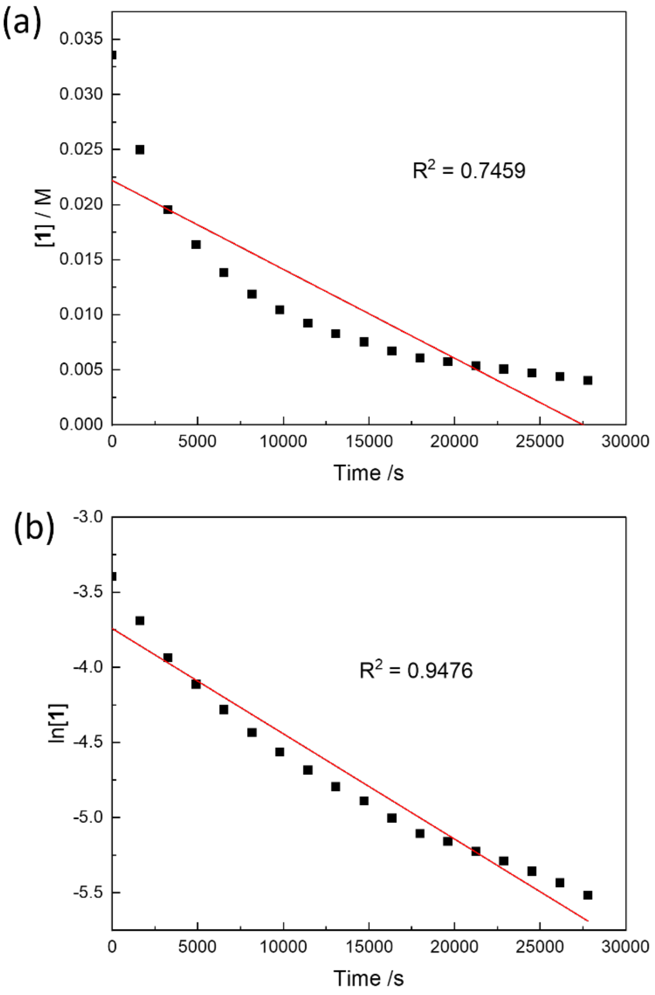

Figure S10. (a) Plot of [**1**] *vs.* time. (b) Plot of ln[**1**] *vs.* time. All reactions were performed with **[1]$_0$** = 0.052 M and T = 60 °C.

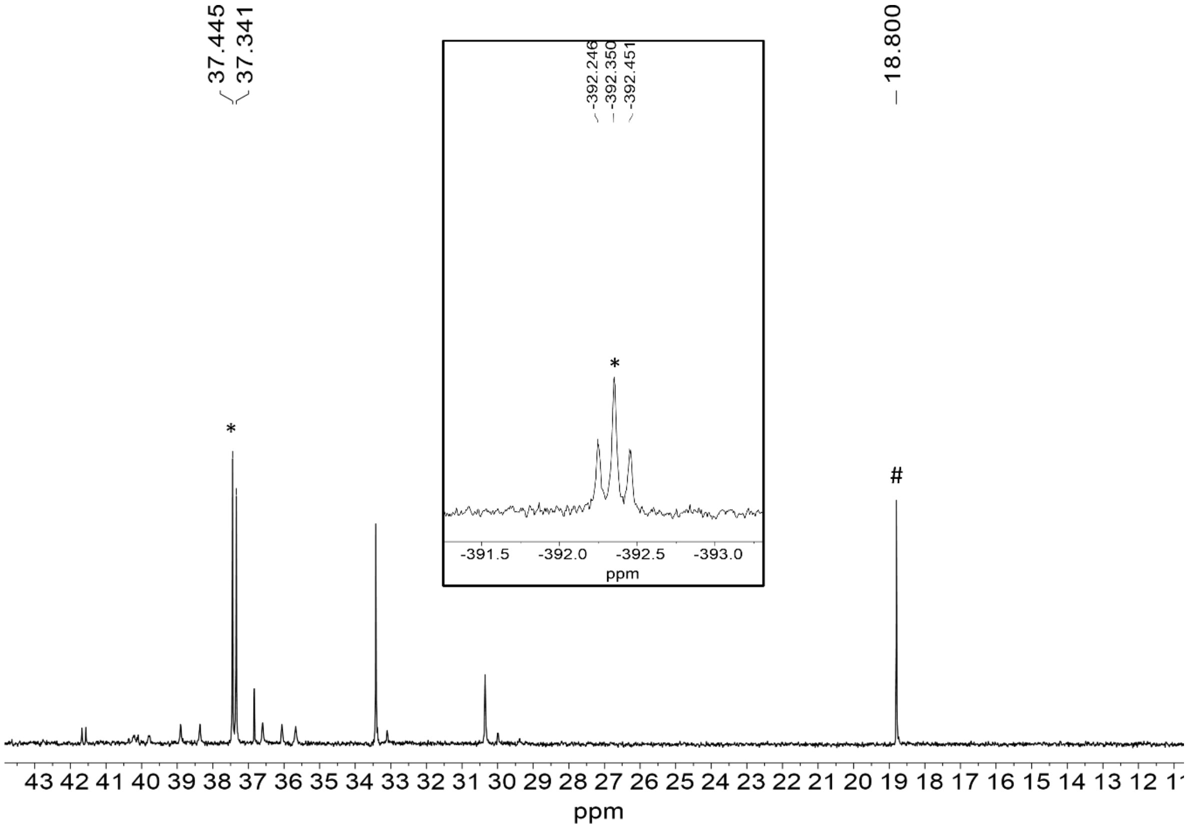

Figure S11. $^{31}P\{^{1}H\}$ NMR (202 MHz, C_6D_6, 25 °C) spectrum of compound **3** contacted with 1 atm CO at 25 ºC which shows production of compounds **1** (*) and **7** (#).

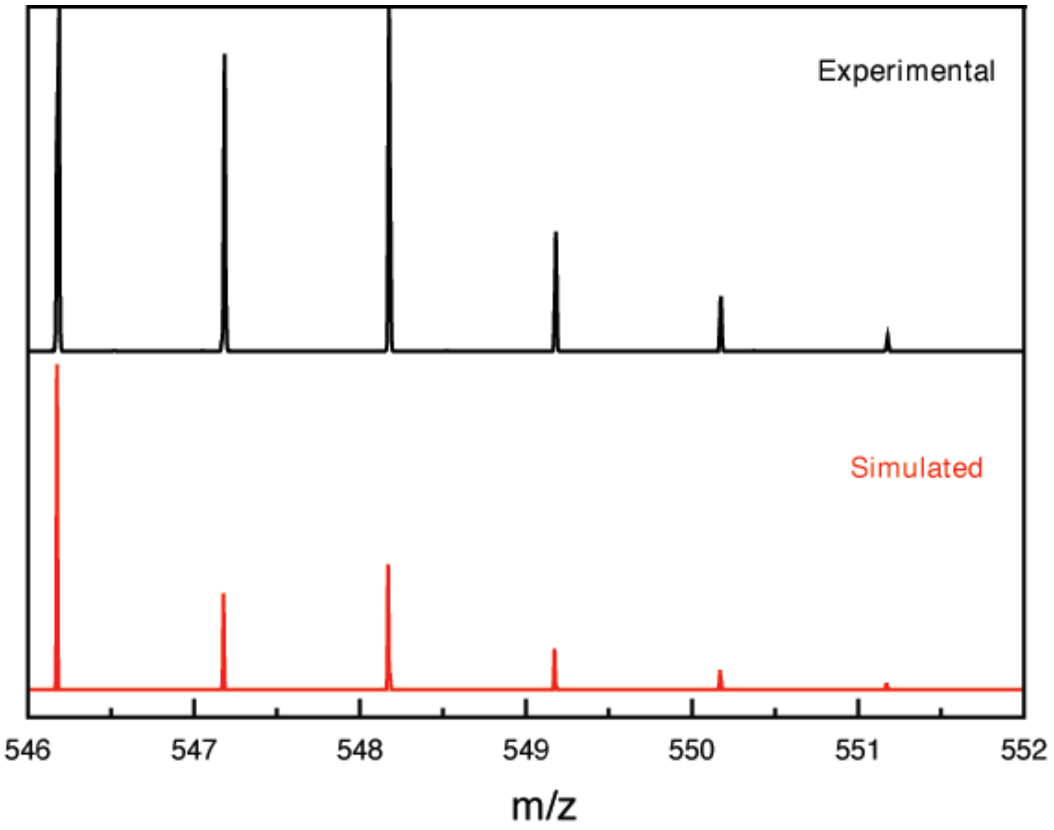

Figure S12. API-MS(+) of $[(PNP)NiPCO(H)]^+$ ($[\mathbf{1}\cdot\mathbf{H}]^+$); black: experimental; red: simulation. The parent *m/z* peak is a 546.17.

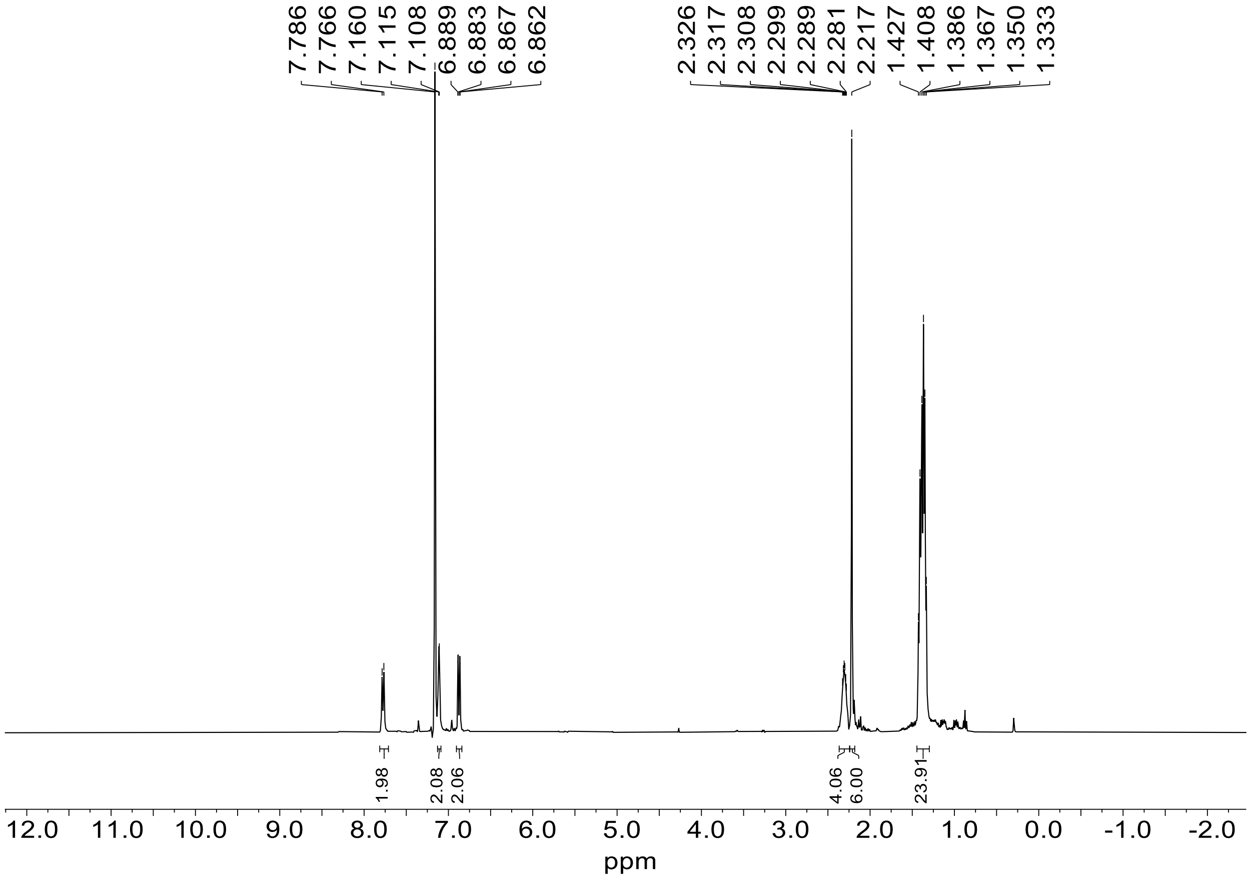

Figure S13. ^{1}H NMR (400 MHz, C_6D_6, 25 °C) spectrum of compound **3**.

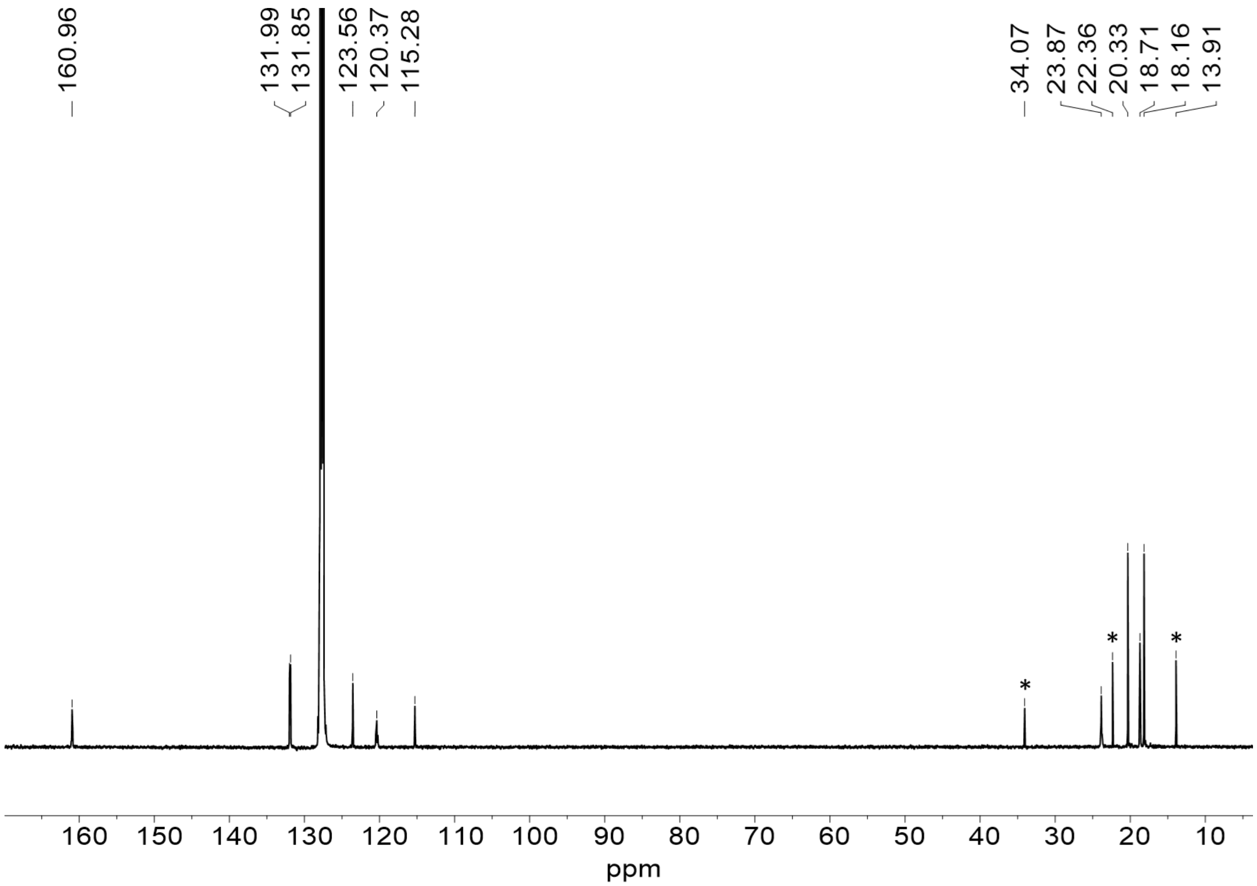

Figure S14. ^{13}C NMR (125 MHz, C_6D_6, 25 °C) spectrum of compound **3**, * denotes pentane.

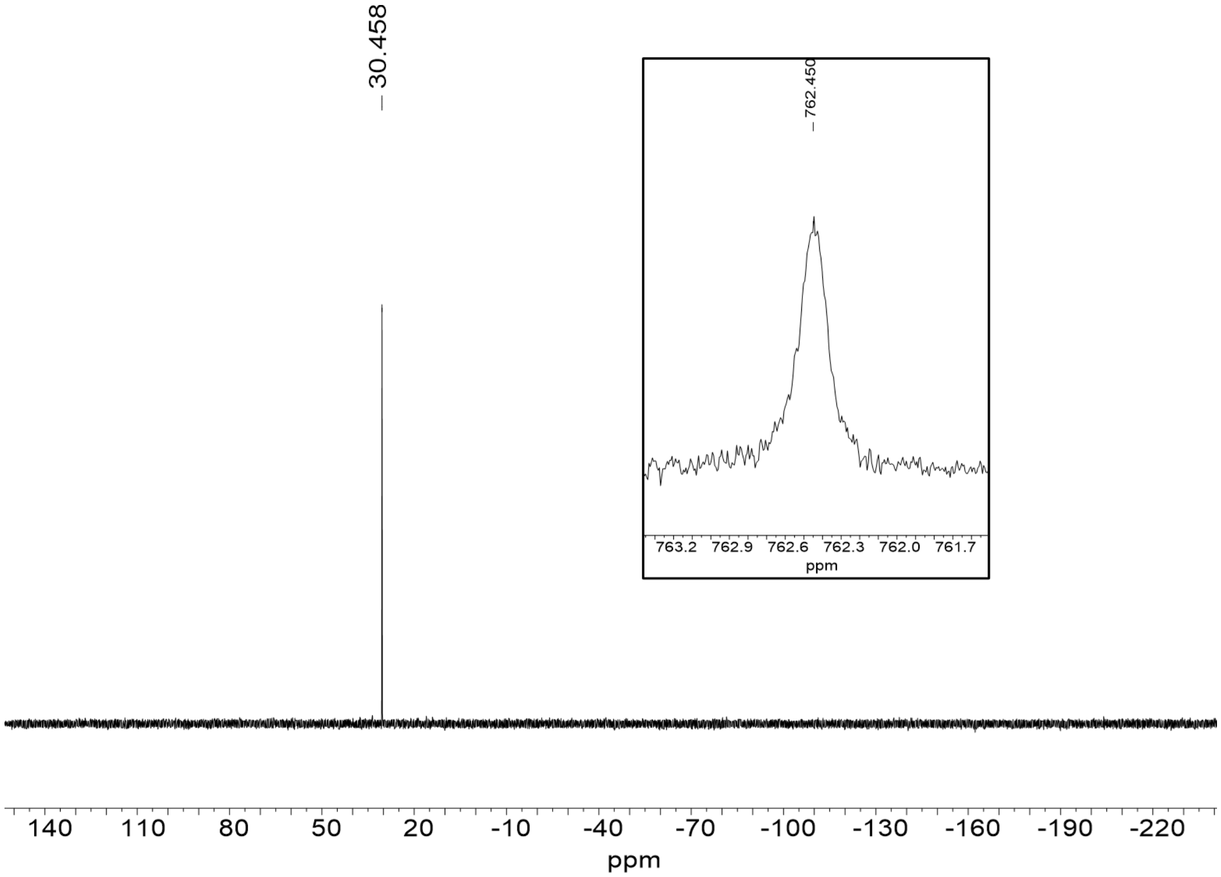

Figure S15. $^{31}P\{^{1}H\}$ NMR (162 MHz, C_6D_6, 25 °C) spectrum of compound **3**. Inset highlights the P_2 signal at ~762 ppm.

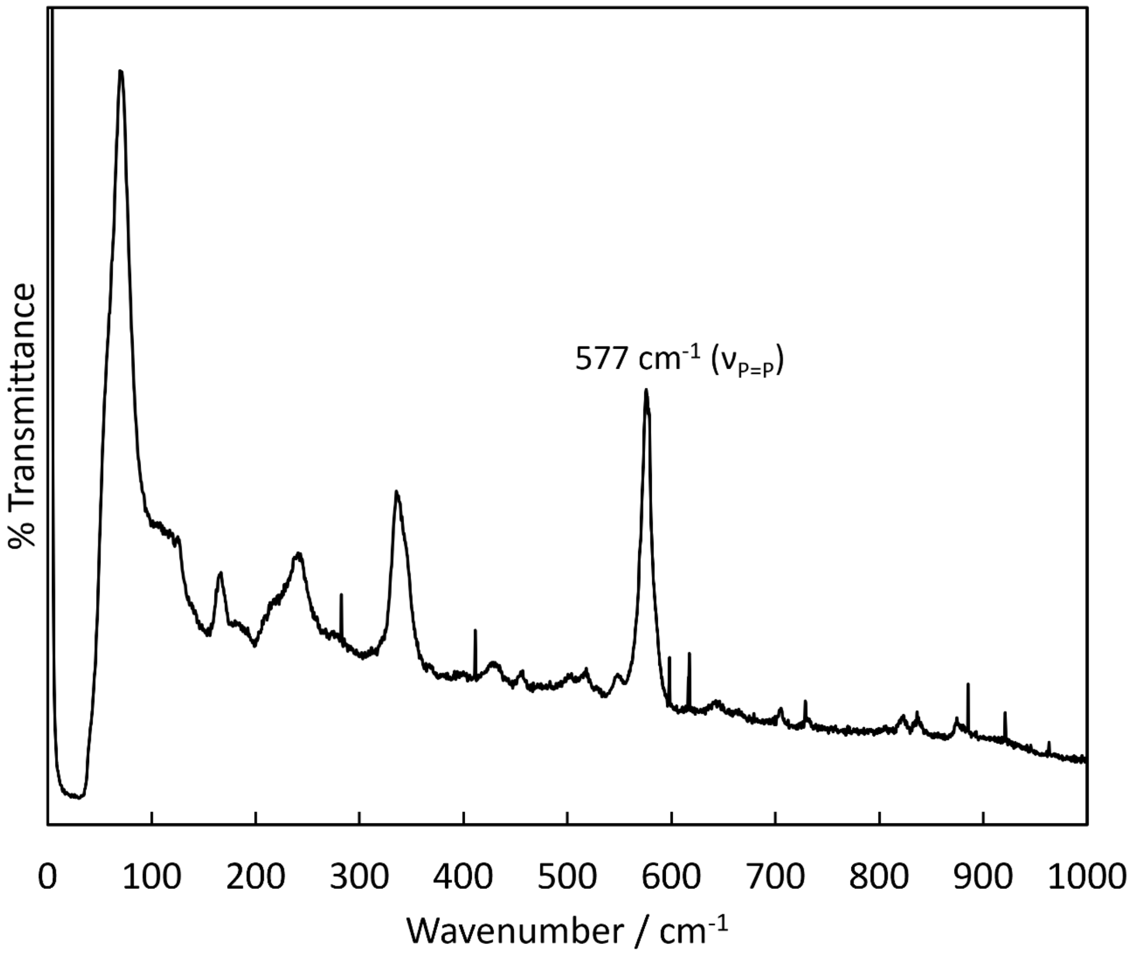

Figure S16. Raman spectrum of compound **3**.

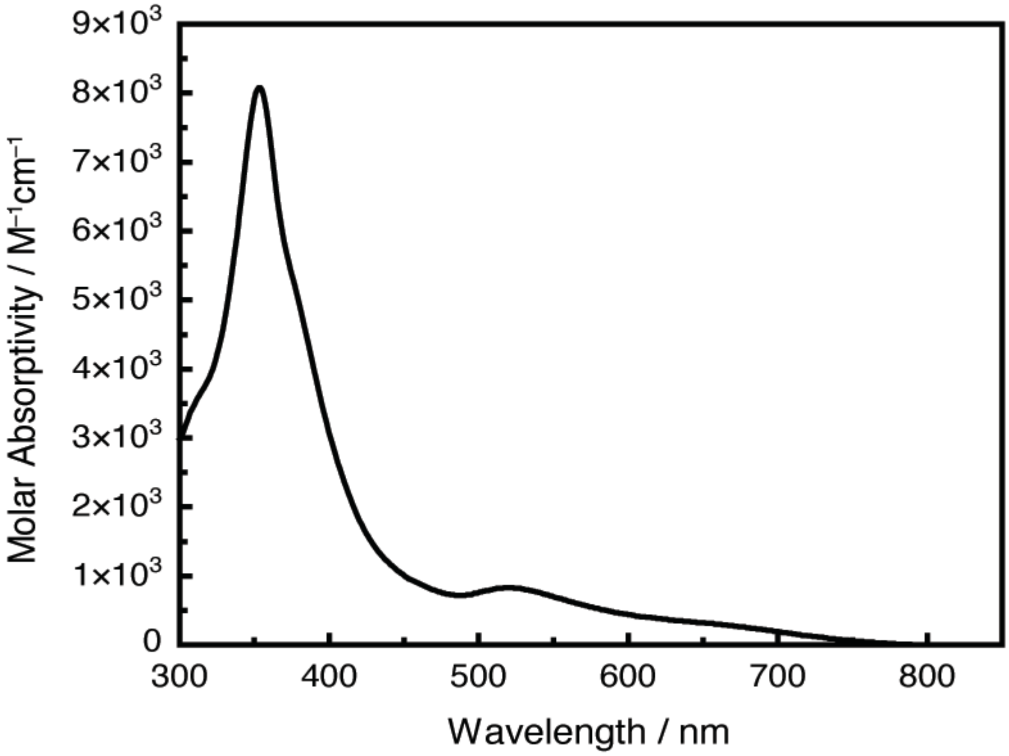

Figure S17. UV-vis absorptivity spectrum (C_6H_6, 25 °C) of compound **3**.

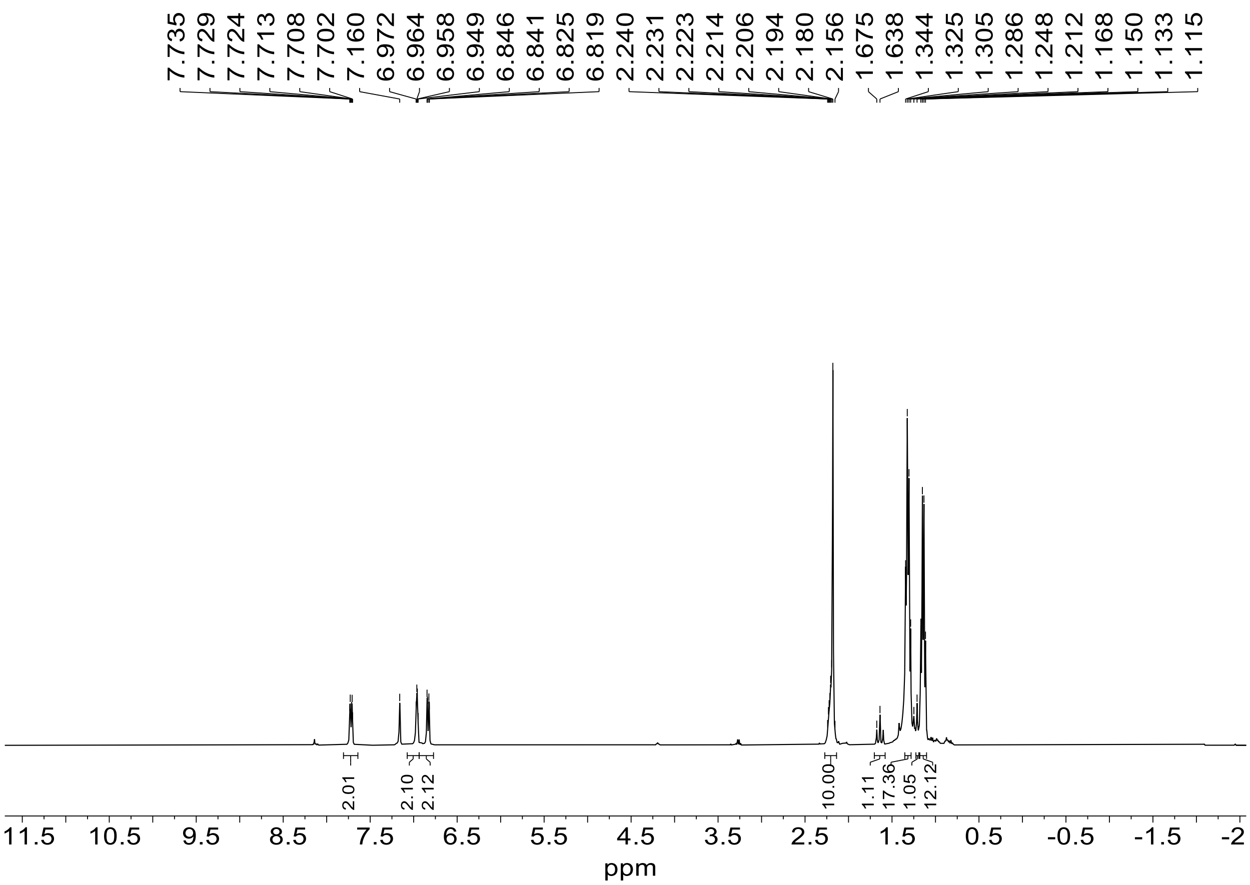

Figure S18. ^{1}H NMR (400 MHz, C_6D_6, 25 °C) spectrum of compound **5**.

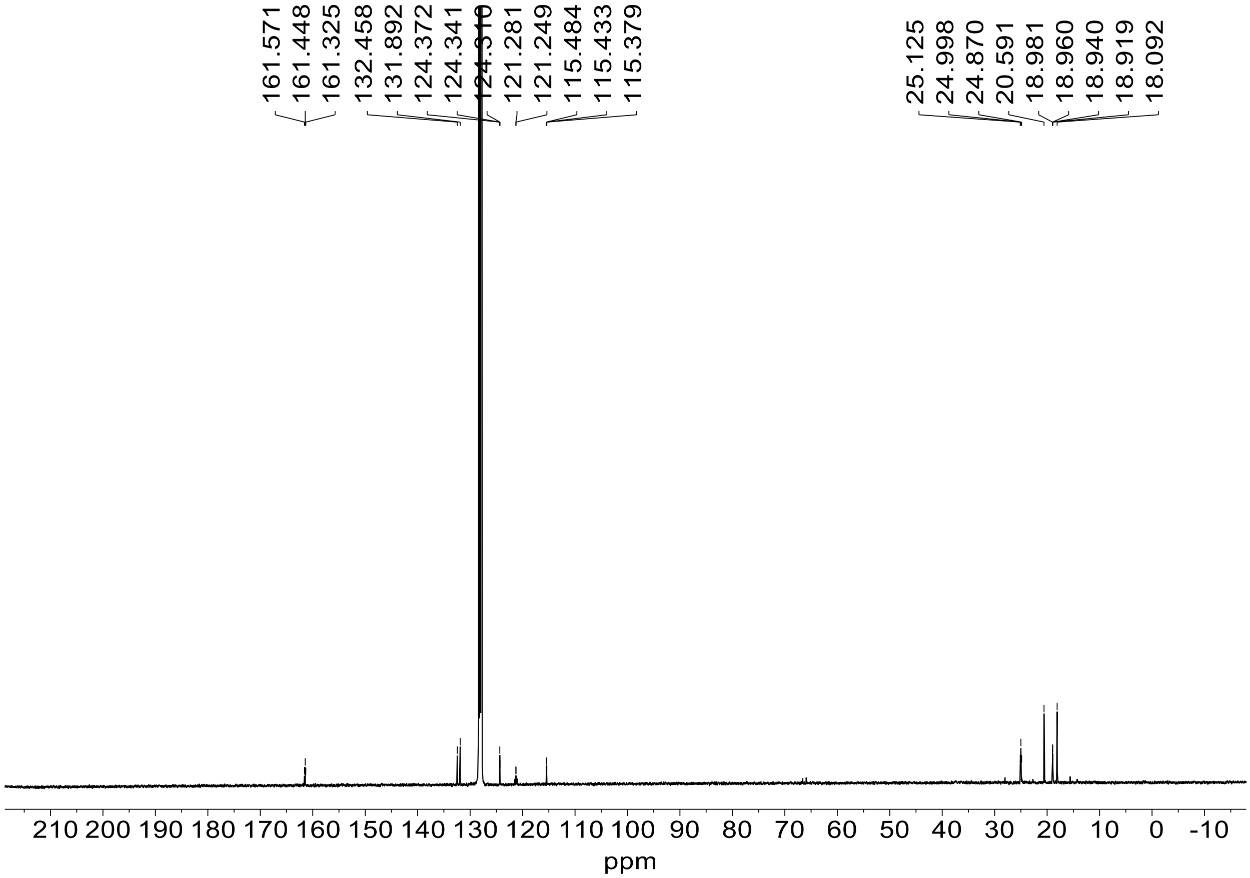

Figure S19. ^{13}C NMR (100 MHz, C_6D_6, 25 °C) spectrum of compound **5**.

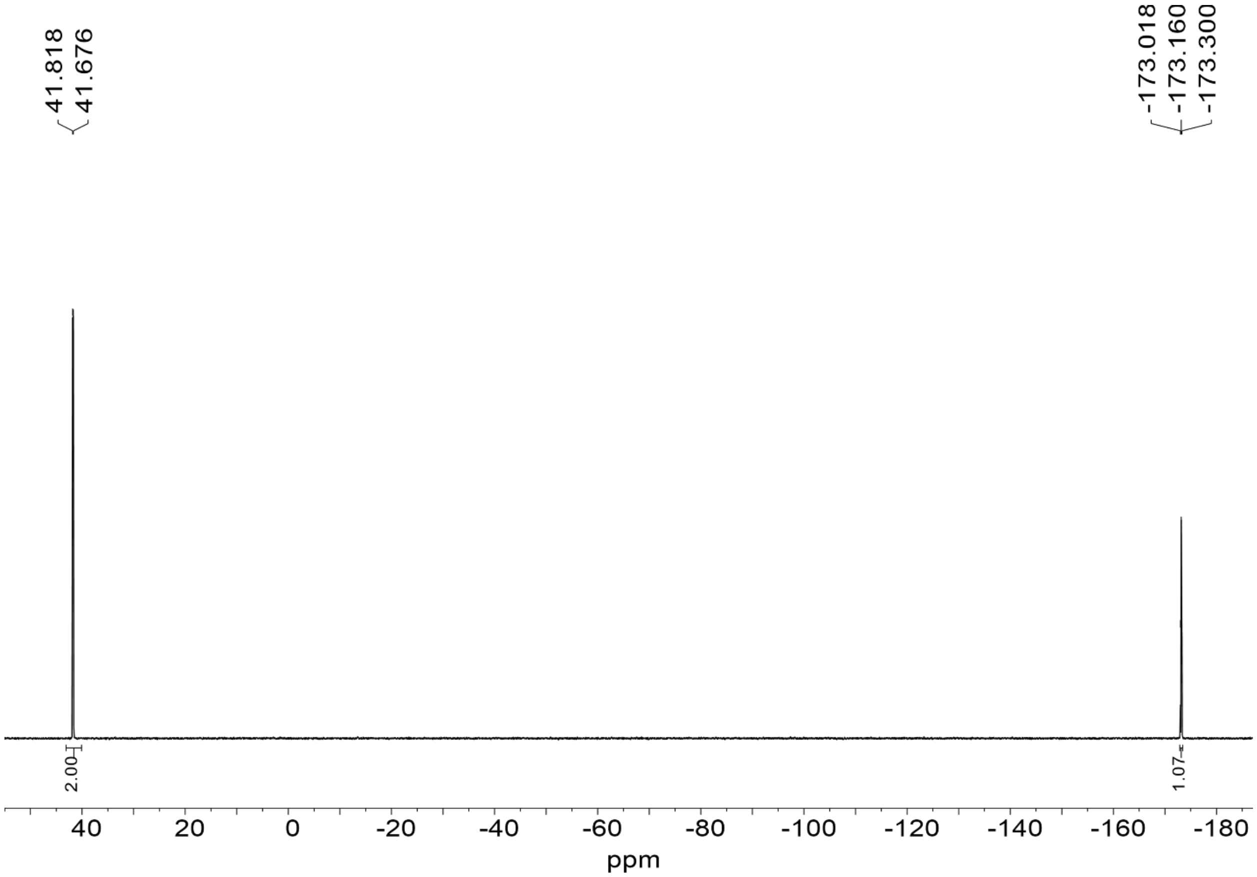

Figure S20. $^{31}P\{^{1}H\}$ NMR (162 MHz, C_6D_6, 25 °C) spectrum of compound **5**.

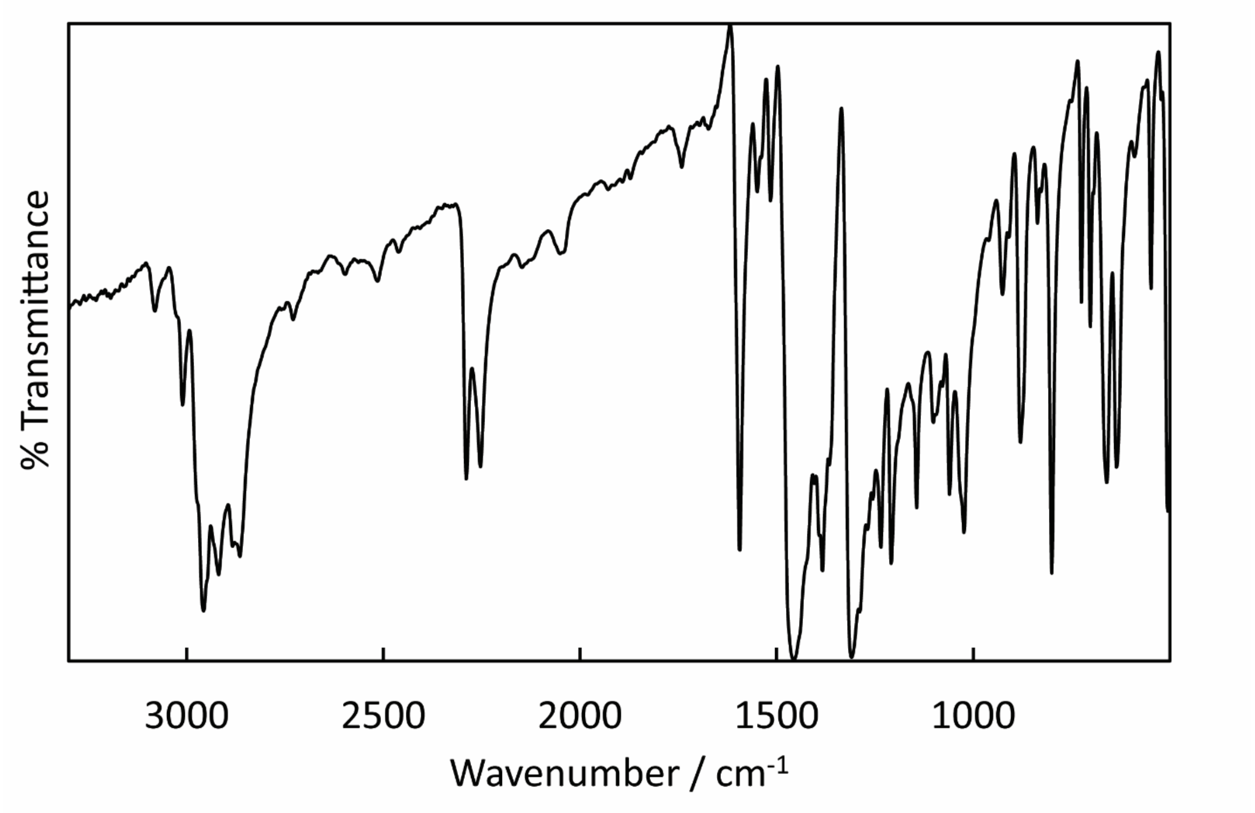

Figure S21. FT-IR spectrum (KBr) of compound **5**.

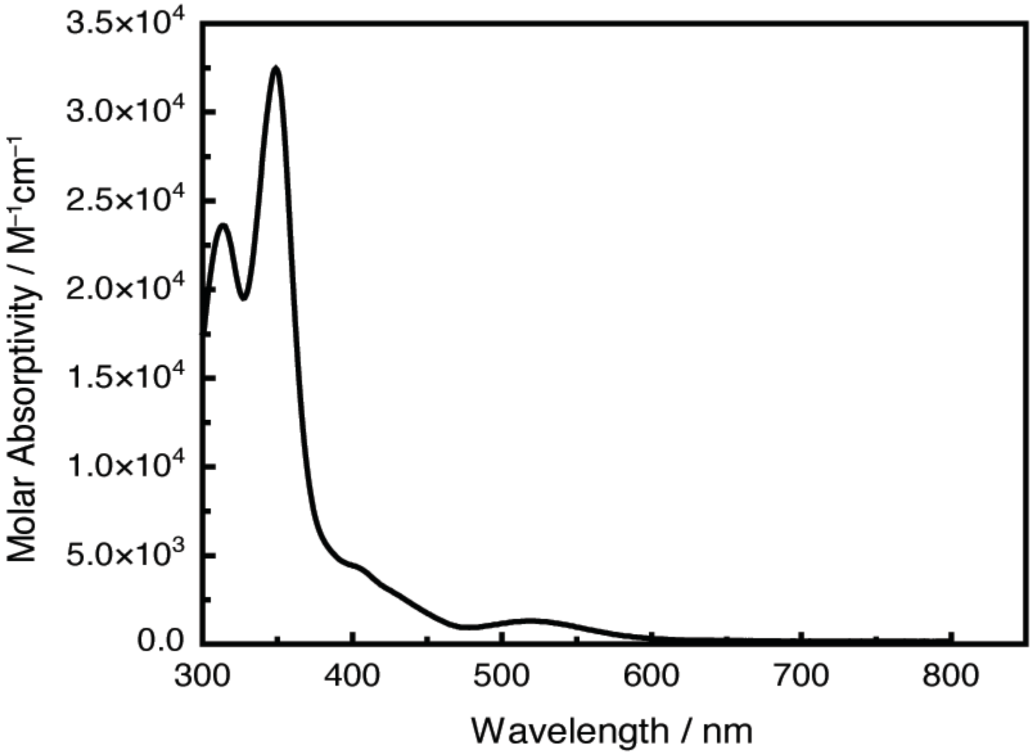

Figure S22. UV-vis spectrum (toluene, 25 °C) of compound **5**.

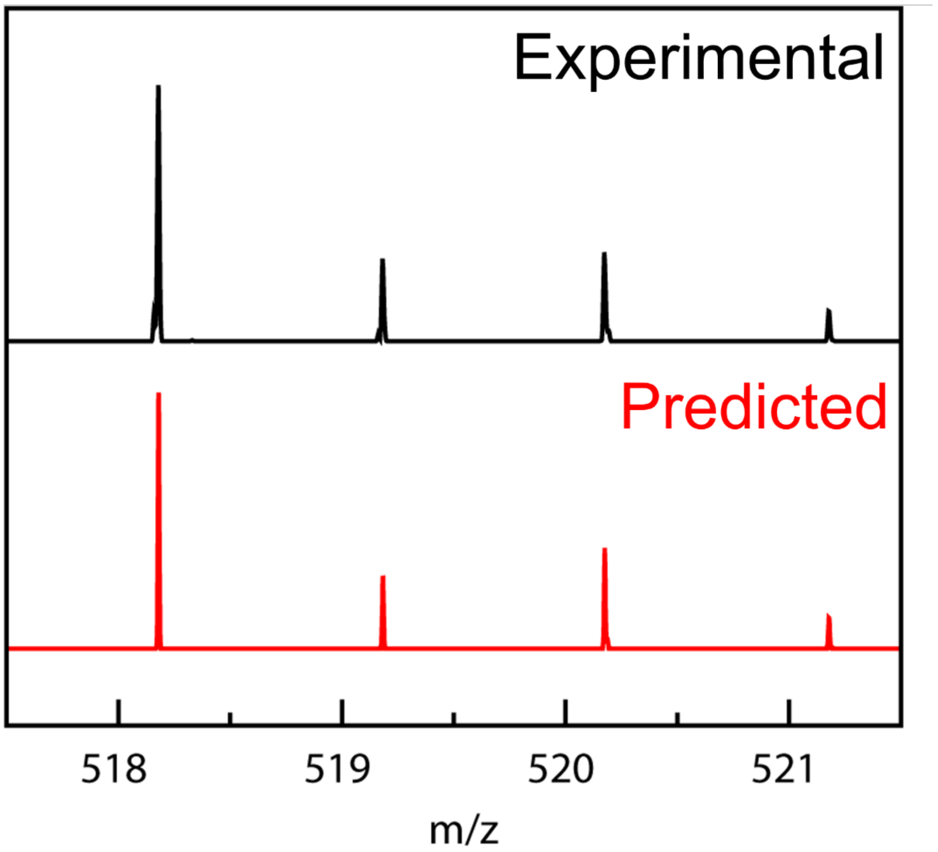

Figure S23. API-MS(+) of [(PNP)NiP(H)]$^+$ ([**2·H**]$^+$); black: experimental; red: simulation. The parent *m/z* peak is a 518.18.

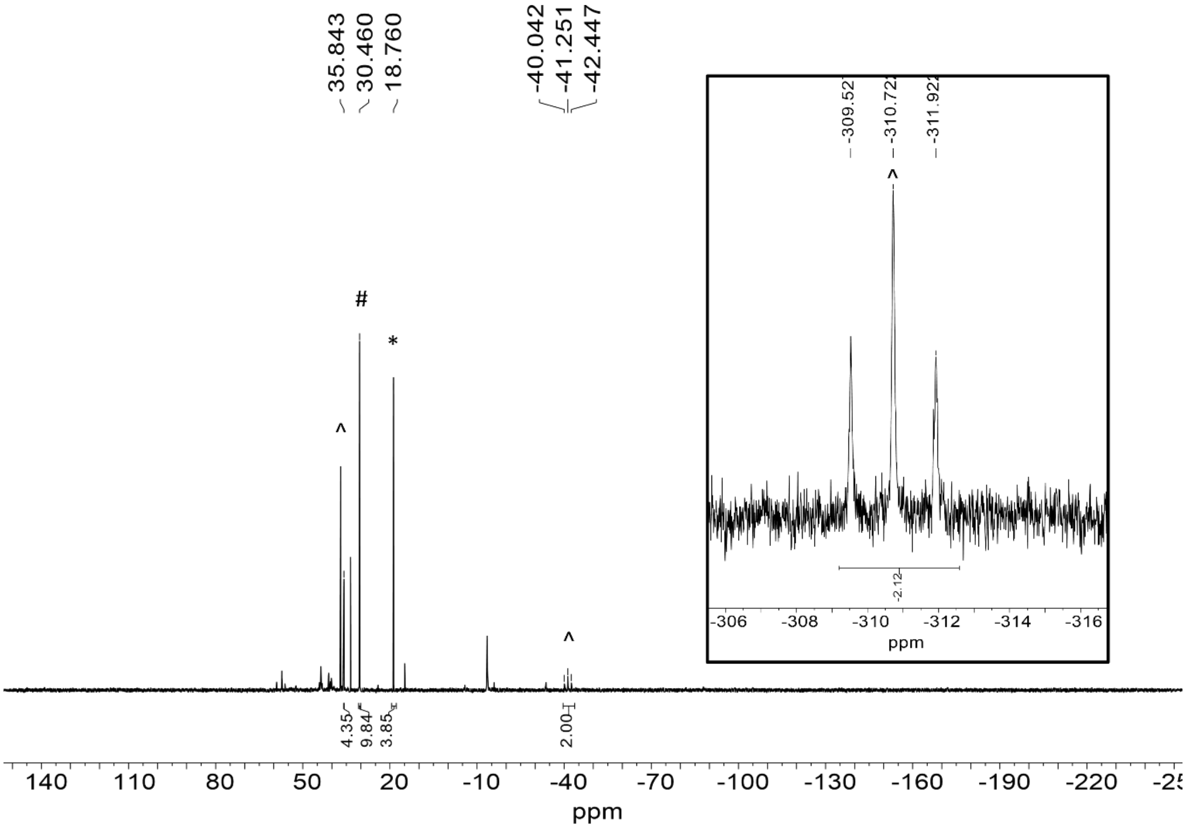

Figure S24. $^{31}P\{^{1}H\}$ NMR (162 MHz, C_6D_6, 25 °C) spectrum of the photolysis of compound **1** to produce compound **3** (#) which reacts further with *in situ* generated $CO_{(g)}$ to form compounds **7** (*) and **8** (^). Triplets centered at –41 and –311 ppm (inset) are ascribed to the *P_4* resonances of **8**.

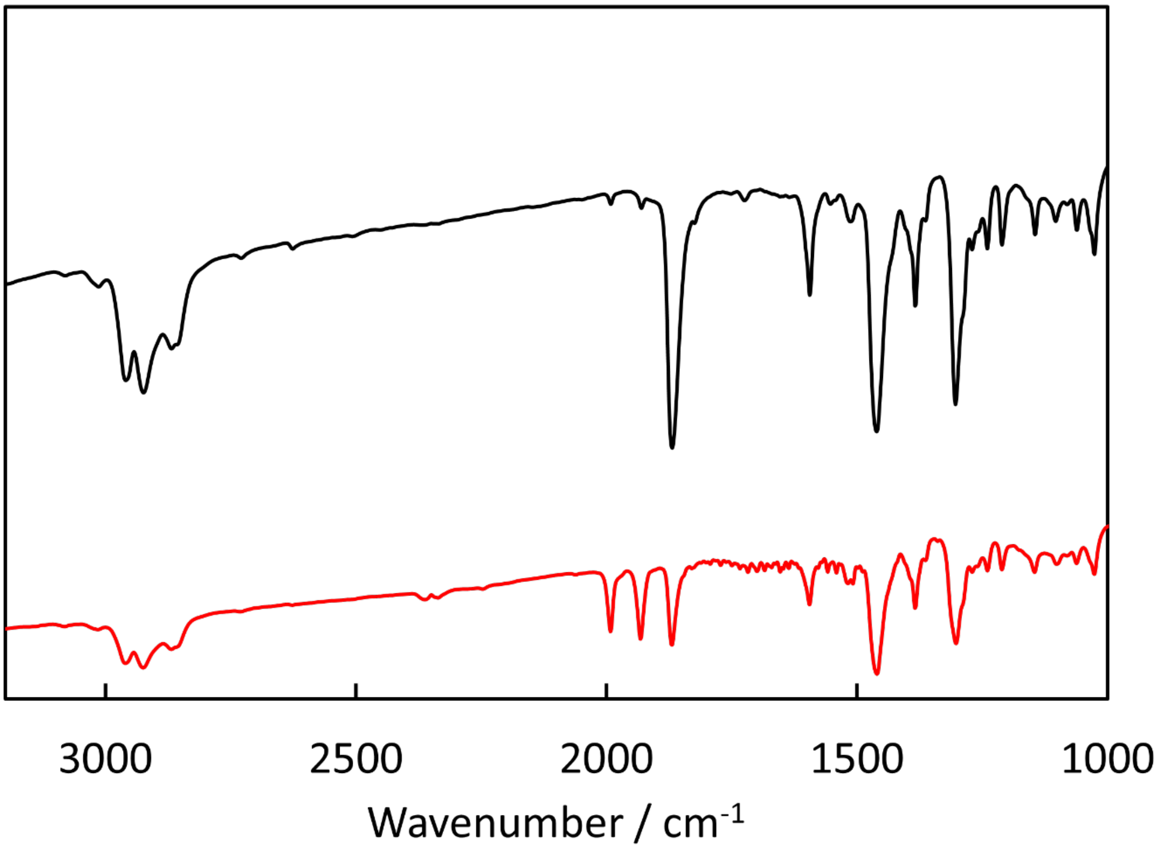

Figure S25. FT-IR spectrum (KBr) of compound **1** before (top, black) and after photolysis (bottom, red), which shows the production of compound **7** due to the growth of characteristic stretching frequencies at 1932 and 1992 cm^{-1}.

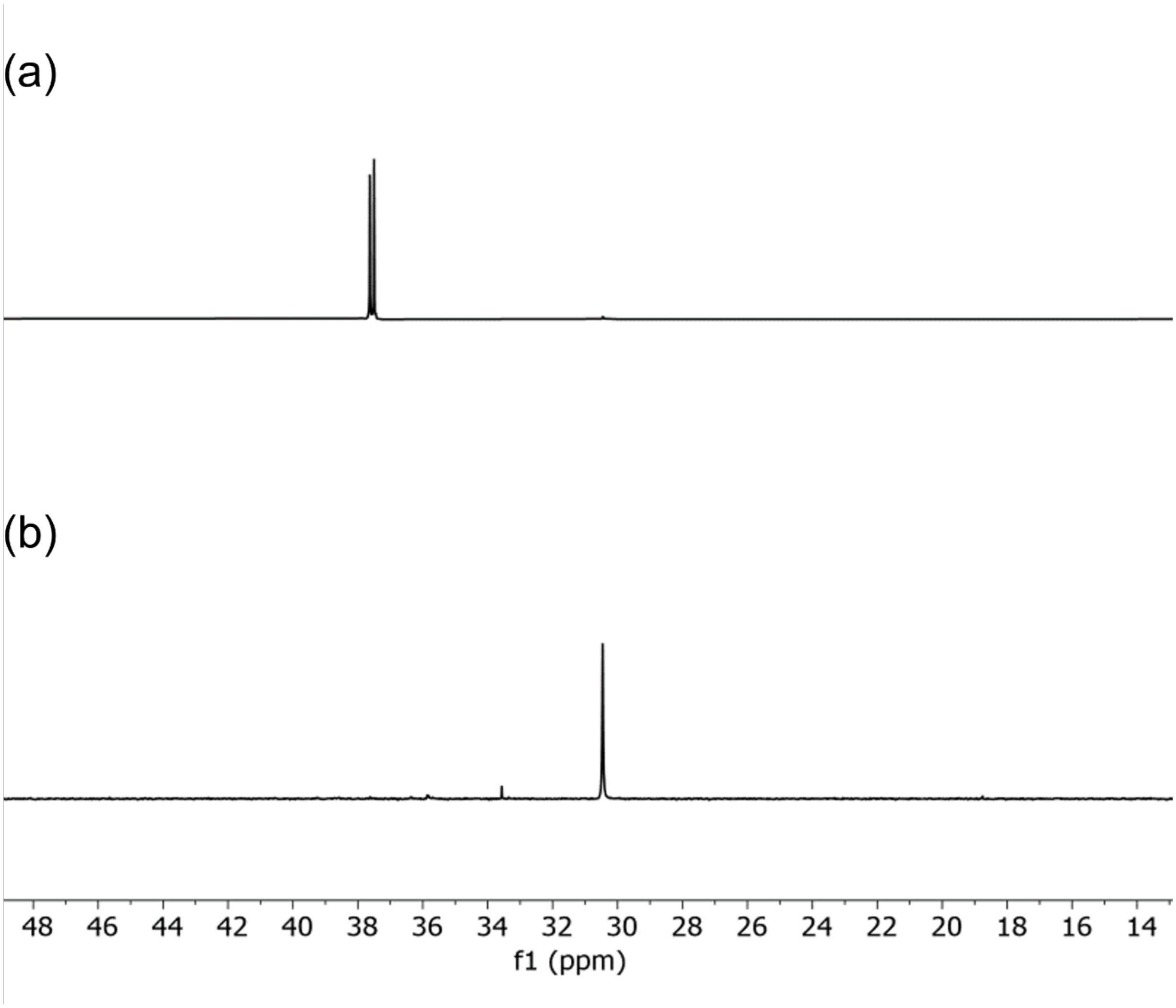

F**igure S26**. Photolysis (λ > 335 nm) of compound **1** in C_6D_6 after 4 freeze-pump-thaw cycles to remove dissolved $CO_{(g)}$ produced via decarbonylation which converts to **3**. $^{31}P\{^1H\}$ NMR (202 MHz, C_6D_6, 25 °C) spectrum at (a) t = 0 h and (b) t = 6 h.

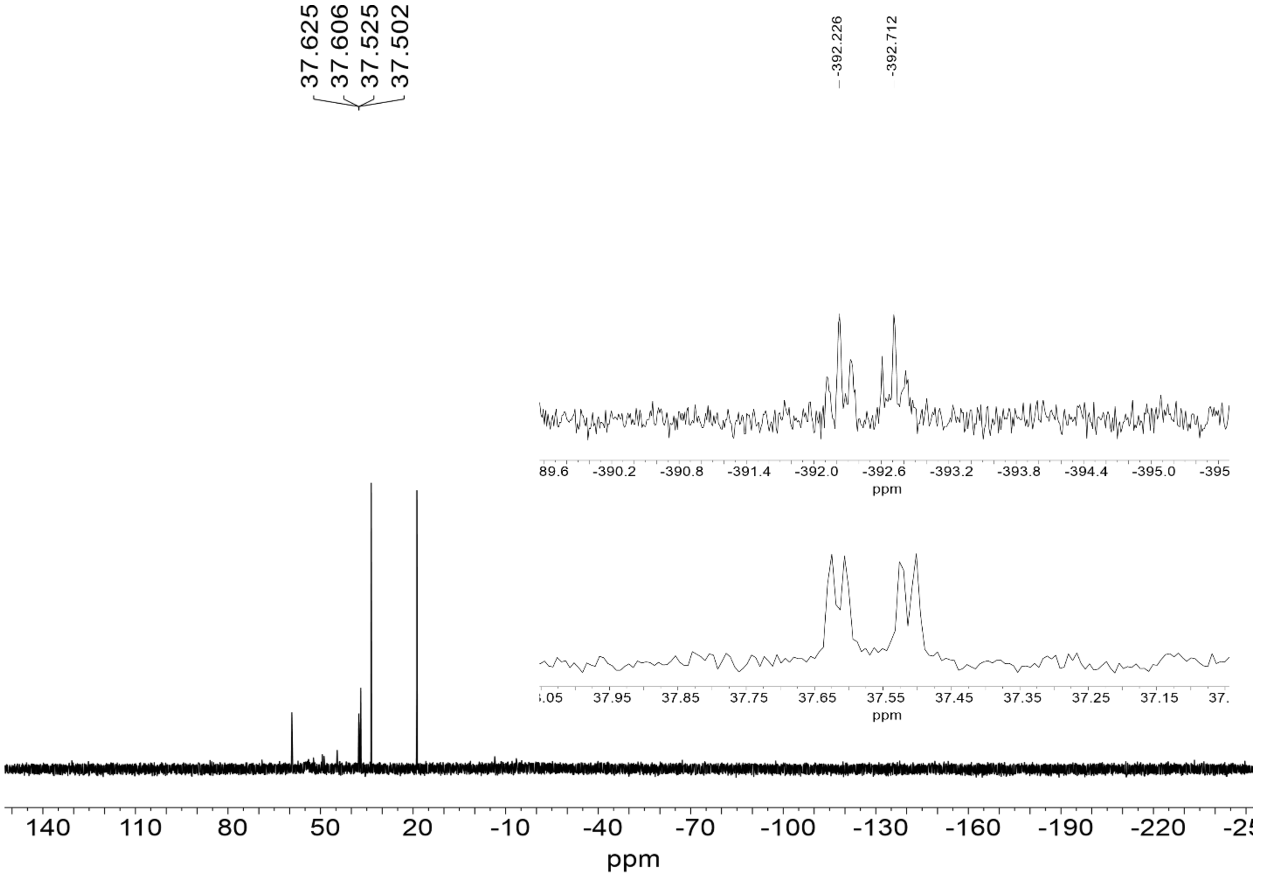

Figure S27. $^{31}P\{^{1}H\}$ NMR (202 MHz, C_6D_6, 25 °C) spectrum of compound ^{13}C-**1** synthesized by exposure of compound **2** to 1 atm of ^{13}CO. Insets show the Ni*P*^{13}CO (top) and *P*N*P* (bottom) resonances.

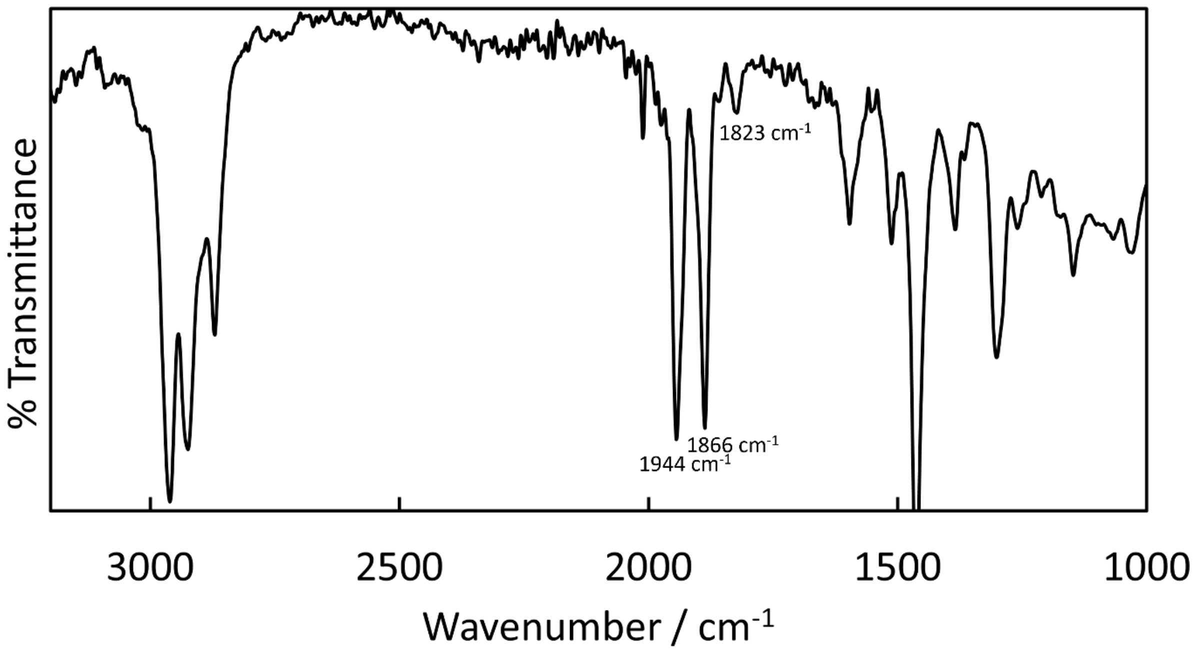

Figure S28. IR spectrum of crude reaction mixture obtained by treating compound **3** with one atmosphere of ^{13}CO. Compounds ^{13}C-**1** (1823 cm^{-1}) and ^{13}C-**7** (1866 cm^{-1}, 1944 cm^{-1}) are evidenced by the indicated stretching modes.

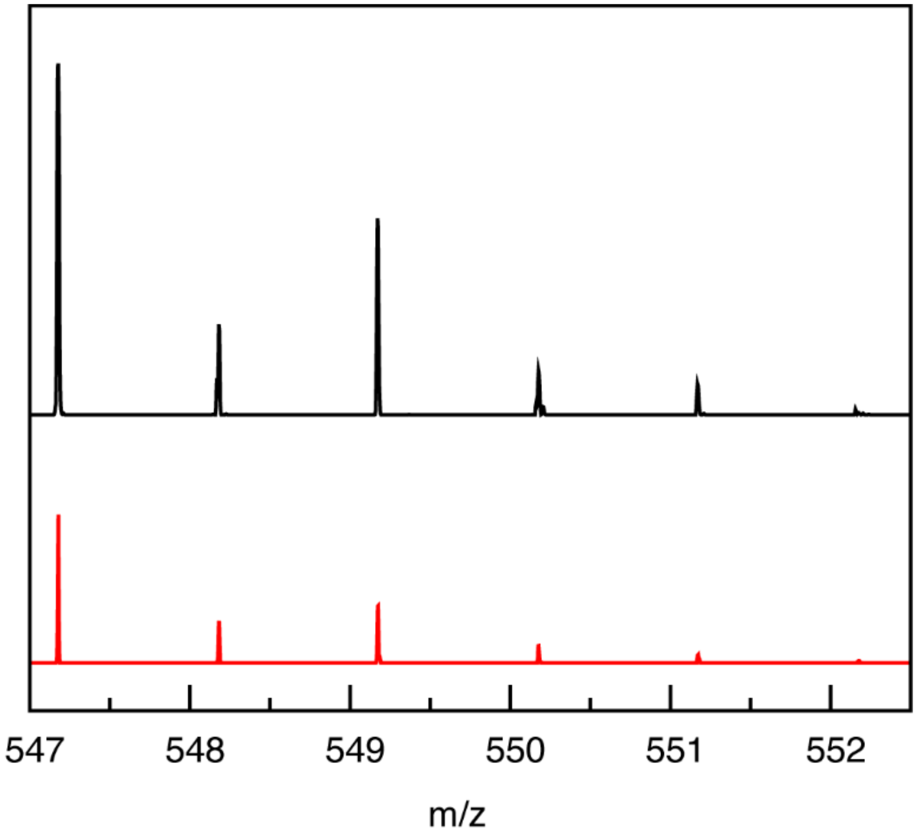

Figure S29. HR ESI-MS (m/z) of ion [^{13}C-**1·H**]$^+$ (calcd. for $C_{26}{}^{13}CH_{41}NNiP_3O^+$, [M + H]$^+$). Top: found, 547.178. Bottom: predicted, 547.175.

E. Reaction Kinetics Data

An N_2-filled J. Young NMR tube was charged with **1** (10 mg, 1.0 equiv) and 0.5 mL of C_6D_6 and was placed in a 500 MHz NMR instrument that had been pre-warmed to 35 °C, 45 °C, 55 °C, or 65 °C. $^{31}P\{^1H\}$ NMR spectra were recorded every 1635 seconds.[a] Conversion was assayed by integration of the resulting $^{31}P\{^1H\}$ NMR spectrum against an internal standard of PPh_3. Time-dependent spectra are collected in Figure S30. A plot of 1/[**1**] *vs.* time was linear, indicating second-order consumption of **1**; other rate laws were evaluated and relevant plots are collected in Figure S10. Second order rate constant of formation of **2** was determined by plotting 1/[**1**] *vs.* time using the equation:

$$\frac{1}{[\mathbf{1}]t} = kt + \frac{1}{[\mathbf{1}]0}$$

where,

$1/[\mathbf{1}]_t$ = concentration of the **1** at time *t*

$1/[\mathbf{1}]_0$ = concentration of the **1** at time $t = 0$

t = time

k = rate constant

Temperature-dependent plots of 1/[**1**] *vs.* time are collected (Figure S31) and the data obtained from these plots were used to construct Eyring (Figure 6) and Arrhenius plots (Figure S32)

[a] The thermal conversion of **1** was also examined under a CO atmosphere and under vacuum to examine the impact of CO pressure of P–P coupling. Thermolysis of **1** under one atmosphere of CO results in a new phosphorous-containing complex, characterized by ^{31}P NMR resonances as 52 ppm (d, J = 16 Hz), 37 ppm (dd, J = 464 Hz, 108 Hz), and –75 ppm (dd, J = 464 Hz, 16 Hz). Given that the reaction products are different (*i.e.*, not P–P coupling to afford compound **3**), we are unable to experimentally evaluate the rate of thermal P–P coupling under an atmosphere of CO. To evaluate the low-pressure limit, we carried out new kinetics experiments using a degassed sample with no head space gas. The rate of thermal P–P coupling at 55 °C was 4.7×10^{-3} $M^{-1}s^{-1}$, which is within error of our original measurement (4.6×10^{-3} $M^{-1}s^{-1}$), which was carried out under N_2. We believe this result is consistent with the mechanism in Figure 7 as both under N_2 and under vacuum, there is very little CO available. Thus, similar kinetics results are obtained.

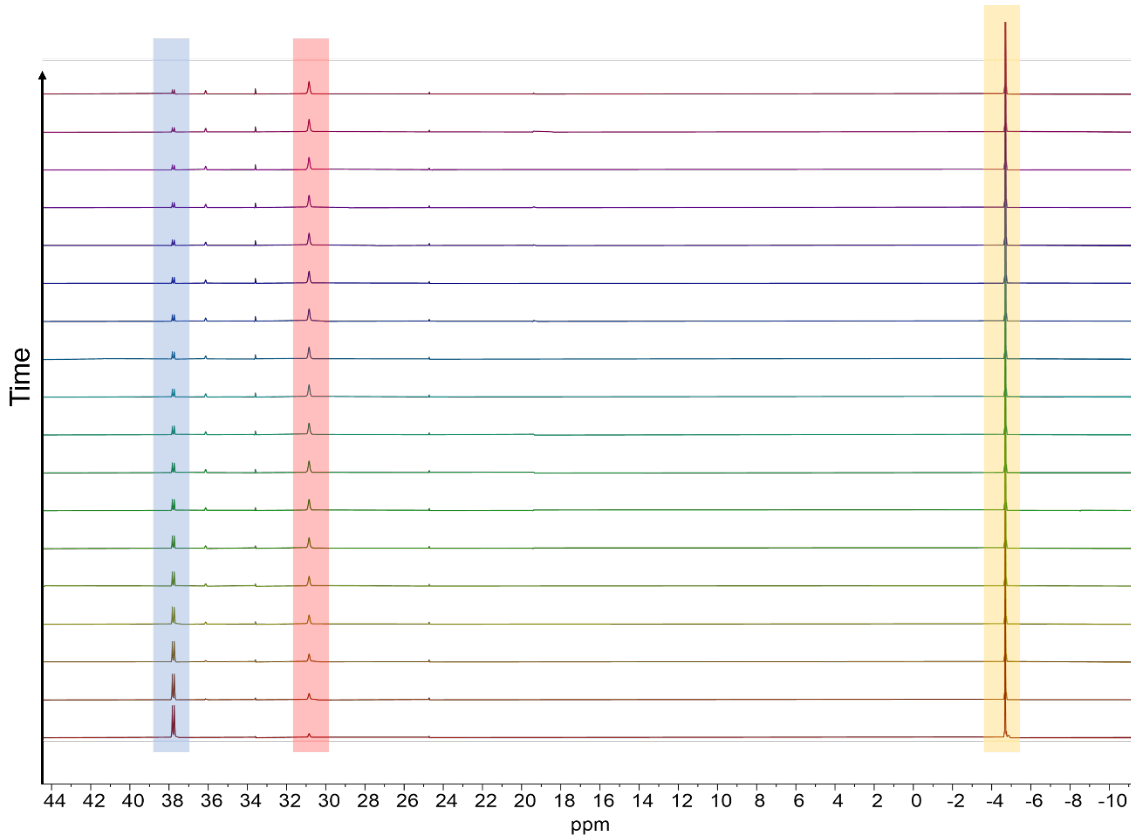

Figure S30. Stacked $^{31}P\{^{1}H\}$ plot (C_6D_6, 60 °C, 202 MHz) tracking conversion of **1**(blue) to **3**(red) over the period of 6 h. Concentration was measured against an PPh_3 (yellow) internal standard.

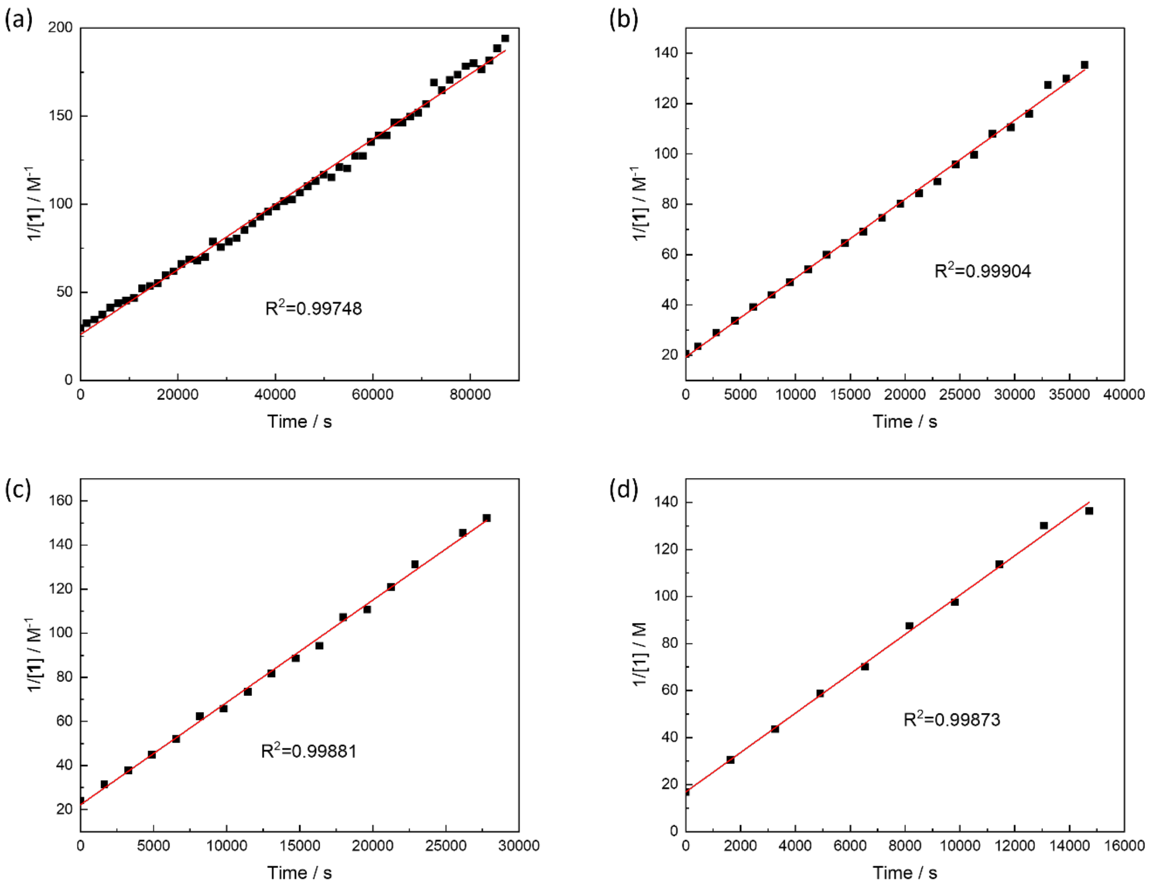

Figure S31. Plot of 1/[**1**] *vs.* time at (a) 35 °C, (b) 45 °C, (c) 55 °C, and (d) 65 °C. All reactions were performed with **[1]$_0$** = 0.052 M.

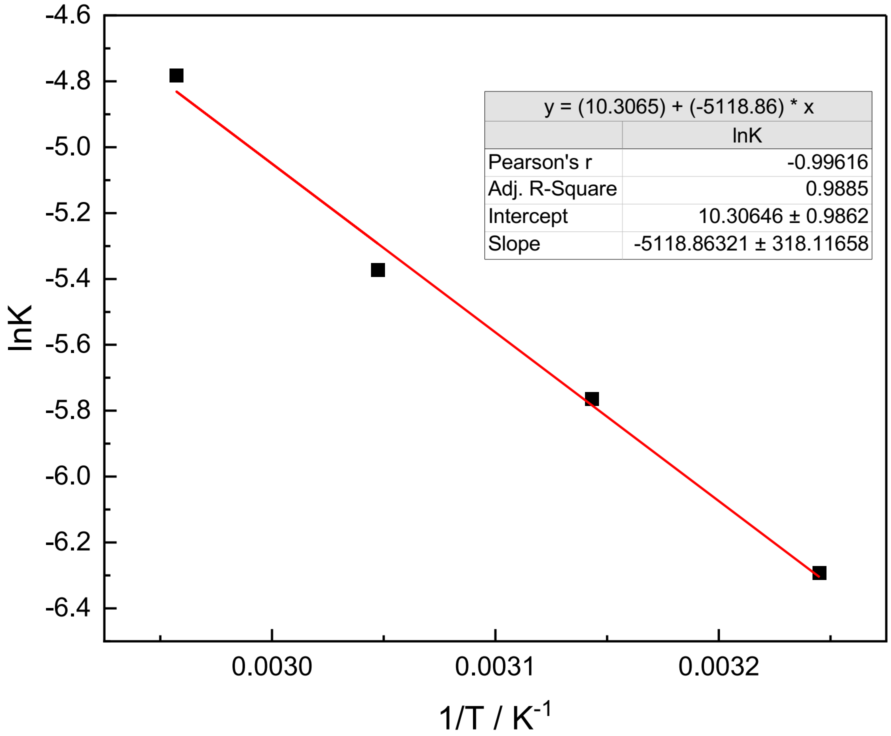

Figure S32. Arrhenius plot for the consumption of **1**.

F. Crystallographic Data

**Table S1.** X-ray experimental details of compound **1** (CCDC 2267449)	
Chemical formula	$0.5(C_{27}H_{40}NNiOP_3)\cdot 0.5(C_7H_8)$
M_r	319.18
Crystal system, space group	Monoclinic, *Pn*
Temperature (K)	111
a, *b*, *c* (Å)	11.6005(5), 9.9499(5), 14.3326(6)
β (°)	93.566(1)
V (Å^3)	1651.1(1)
Z	4
Radiation type	Mo *K*α
μ (mm^{-1})	0.76
Crystal size (mm)	0.29 × 0.21 × 0.16
Data collection	
Diffractometer	Bruker *APEX*-II CCD
Absorption correction	Multi-scan<br>*SADABS2016*/2 (Bruker,2016/2) was used for absorption correction. wR2(int) was 0.0829 before and 0.0528 after correction. The ratio of minimum to maximum transmission is 0.9087. The λ/2 correction factor is not present.
T_{min}, T_{max}	0.678, 0.746
No. of measured, independent and observed [$I > 2\sigma(I)$] reflections	23612, 9098, 8038
R_{int}	0.037
$(\sin \theta/\lambda)_{max}$ (Å^{-1})	0.697
Refinement	
$R[F^2 > 2\sigma(F^2)]$, $wR(F^2)$, S	0.042, 0.109, 1.08
No. of reflections	9098
No. of parameters	373
No. of restraints	2
H-atom treatment	H-atom parameters constrained
$\Delta\rho_{max}$, $\Delta\rho_{min}$ (e Å^{-3})	0.74, −0.74
Absolute structure	Flack x determined using 3354 quotients [(I+)-(I-)]/[(I+)+(I-)] (Parsons, Flack and Wagner, Acta Cryst. B69 (2013) 249-259).
Absolute structure parameter	−0.016(7)

Table S2. X-ray experimental details of compound **3** (CCDC 2267446)

Chemical formula	$C_{52}H_{80}N_2Ni_2P_6$
M_r	1036.42
Crystal system, space group	Orthorhombic, *Pbca*
Temperature (K)	110
a, *b*, *c* (Å)	17.791(2), 14.6002(1), 20.251(2)
V (Å^3)	5260.3(8)
Z	4
Radiation type	Mo *K*α
μ (mm^{-1})	0.93
Crystal size (mm)	0.21 × 0.11 × 0.11
**Data collection**	
Diffractometer	Bruker *APEX*-II CCD
Absorption correction	Multi-scan<br>*SADABS2016*/2 (Bruker,2016/2) was used for absorption correction. wR2(int) was 0.1318 before and 0.0705 after correction. The ratio of minimum to maximum transmission is 0.8011. The λ/2 correction factor is not present.
T_{min}, T_{max}	0.212, 0.264
No. of measured, independent and observed [$I > 2\sigma(I)$] reflections	35266, 7295, 5728
R_{int}	0.063
$(\sin \theta/\lambda)_{max}$ (Å^{-1})	0.695
**Refinement**	
$R[F^2 > 2\sigma(F^2)]$, $wR(F^2)$, S	0.077, 0.157, 1.17
No. of reflections	7295
No. of parameters	373
No. of restraints	84
H-atom treatment	H-atom parameters constrained<br>$w = 1/[\sigma^2(F_o^2) + (0.0143P)^2 + 13.9121P]$<br>where $P = (F_o^2 + 2F_c^2)/3$
$\Delta\rho_{max}$, $\Delta\rho_{min}$ (e Å^{-3})	0.74, −0.74

**Table S3.** X-ray experimental details of compound **5** (CCDC 2267448)	
Chemical formula	$C_{26}H_{42}NNiP_3{\cdot}(C_7H_8)$
M_r	611.51
Crystal system, space group	Monoclinic, *P2₁/c*
Temperature (K)	110
a, *b*, *c* (Å)	21.3247(8), 9.5257(6), 27.996(2)
β (°)	92.551(2)
V (Å^3)	3283.5(4)
Z	4
Radiation type	Cu *K*α
μ (mm^{-1})	2.39
Crystal size (mm)	0.23 × 0.08 × 0.03
Data collection	
Diffractometer	Bruker *APEX*-II CCD
Absorption correction	Multi-scan<br>*SADABS2016*/2 (Bruker,2016/2) was used for absorption correction. wR2(int) was 0.1085 before and 0.0770 after correction. The Ratio of minimum to maximum transmission is 0.8113. The λ/2 correction factor is not present.
T_{min}, T_{max}	0.612, 0.754
No. of measured, independent and observed [$I > 2\sigma(I)$] reflections	88142, 7076, 6415
R_{int}	0.056
$(\sin \theta/\lambda)_{max}$ (Å^{-1})	0.637
Refinement	
$R[F^2 > 2\sigma(F^2)]$, $wR(F^2)$, S	0.041, 0.106, 1.03
No. of reflections	7076
No. of parameters	373
H-atom treatment	H-atoms treated by a mixture of independent and constrained refinements
$\Delta\rho_{max}$, $\Delta\rho_{min}$ (e Å^{-3})	0.86, −0.45

**Table S4.** X-ray experimental details of compound **7** (CCDC 2308904)	
Chemical formula	$C_{28}H_{41}NNiO_2P_2$
M_r	544.27
Crystal system, space group	Monoclinic, $P2_1$/c
Temperature (K)	110
a, *b*, *c* (Å)	13.5909(5), 12.4510(4), 16.9181(6)
β (°)	96.827(2)
V ($Å^3$)	2842.6(2)
Z	4
Radiation type	Mo *K*α
μ (mm^{-1})	0.82
Crystal size (mm)	0.12 × 0.05 × 0.03
Data collection	
Diffractometer	Bruker *APEX*-II CCD
Absorption correction	Multi-scan<br>*SADABS2016*/2 (Bruker,2016/2) was used for absorption correction. wR2(int) was 0.1271 before and 0.0741 after correction. The ratio of minimum to maximum transmission is 0.7512. The λ/2 correction factor is not present.
T_{min}, T_{max}	0.561, 0.746
No. of measured, independent and observed [$I > 2\sigma(I)$] reflections	59505, 8652, 6690
R_{int}	0.091
$(\sin \theta/\lambda)_{max}$ ($Å^{-1}$)	0.997
Refinement	
$R[F^2 > 2\sigma(F^2)]$, $wR(F^2)$, S	0.040, 0.105, 1.04
No. of reflections	8652
No. of parameters	317
H-atom treatment	H-atom parameters constrained
$\Delta\rho_{max}$, $\Delta\rho_{min}$ (e $Å^{-3}$)	0.99, −0.78

Table S5. X-ray experimental details of compound **8** (CCDC 2267445)

Chemical formula	$C_{52}H_{80}N_2Ni_2P_8$
M_r	1198.36
Crystal system, space group	Tetragonal, $I4_1/a$
Temperature (K)	110
a, *b*, *c* (Å)	37.340(3), 37.340(3), 18.273(2)
V (Å^3)	25477(5)
Z	16
Radiation type	Mo *K*α
μ (mm^{-1})	0.82
Crystal size (mm)	0.12 × 0.05 × 0.03
Data collection	
Diffractometer	Bruker *APEX*-II CCD
Absorption correction	Multi-scan<br>*SADABS2016*/2 (Bruker,2016/2) was used for absorption correction. wR2(int) was 0.1417 before and 0.1075 after correction. The ratio of minimum to maximum transmission is 0.8662. The λ/2 correction factor is not present.
T_{min}, T_{max}	0.6131, 0.7078
No. of measured, independent and observed [$I > 2\sigma(I)$] reflections	339685, 15553, 9673
R_{int}	0.335
$(\sin \theta/\lambda)_{max}$ (Å^{-1})	0.669
Refinement	
$R[F^2 > 2\sigma(F^2)]$, $wR(F^2)$, S	0.110, 0.223, 1.06
No. of reflections	15553
No. of parameters	599
H-atom treatment	H-atom parameters constrained
$\Delta\rho_{max}$, $\Delta\rho_{min}$ (e Å^{-3})	0.78, −0.40

G. Computational Data

Calculation of P−H Bond Dissociation Energies for Complex 5

Bond dissociation energies (BDEs)were calculated with corrections for the basis set superposition error (BSSE) using Boys and Bernardi´s counterpoise method,[24-26] as implemented in Gaussian, according to the following equation:

$$\Delta E = \Delta E_{el} + ZPE(AB) - ZPE(A) - ZPE(B) - \delta^{BSSE} \qquad (1)$$

where $\Delta E_{el} = E_{AB}^{AB}(AB)$-$E_{A}^{A}(A)$-$E_{B}^{B}(B)$. Here $E_{Z}^{Y}(X)$ represents the energy of subsystem X at the optimized geometry Y using the basis set Z. The counterpoise correction is defined as:[3]

$$\delta^{BSSE} = E_{AB}^{A}(A) + E_{AB}^{B}(B) - E_{AB}^{AB}(A) - E_{AB}^{AB}(B) \qquad (2)$$

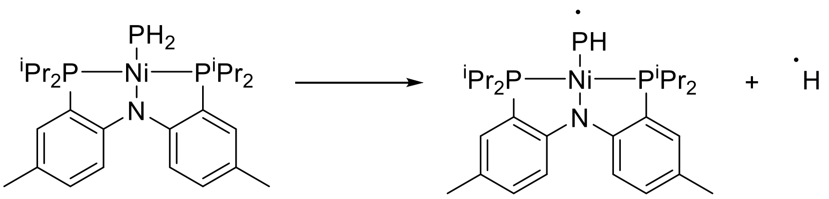

^{1}**AB** ^{2}**A** ^{2}**B**

Method	Sum of electronic and thermal Free Energies			δ^{BSSE}(a.u.)	BDE (kcal/mol)
	^{1}AB (a.u.)	^{2}A (a.u.)	^{2}B (a.u.)		
UB3LYP-D3/def2svp-CPCM(benzene)	-3601.670501	-3601.054828	-0.511922	N/A	65.1
UB3LYP-D3/def2tzvpp-CPCM(benzene)//UB3LYP-D3/def2svp-CPCM(benzene)	-3603.418094	-3602.800133	-0.51282032	N/A	66.0
UB3LYP-D3/6-311+g(d,p)-CPCM(benzene)//UB3LYP-D3/def2svp-CPCM(benzene)	-3603.199945	-3602.583157	-0.512822064	N/A	65.2
UB3LYP-D3/def2svp	-3601.665792	-3601.049714	-0.511913	0.001646142	66.4
UB3LYP-D3/def2tzvpp//UB3LYP-D3/def2svp	-3603.413319	-3602.794977	-0.512808224	0.000308438	66.4
UM06L-D3/def2tzvpp//UB3LYP-D3/def2svp	-3603.026107	-3602.410832	-0.514069617	0.000462598	63.8
UPBE0-D3/def2tzvpp//UB3LYP-D3/def2svp	-3601.338712	-3600.72493	-0.511689723	0.000283585	64.2

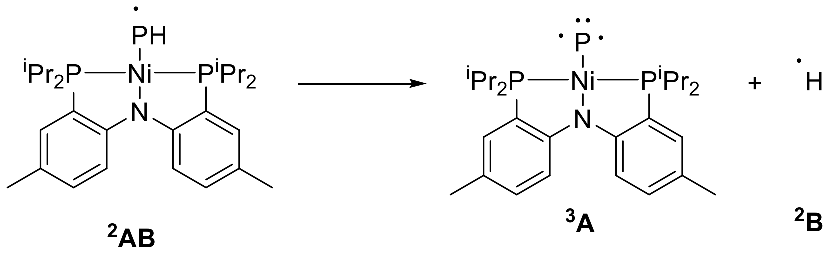

Method	Sum of electronic and thermal Free Energies			δ^{BSSE}*(a.u.)*	BDE (kcal/mol)
	^{2}AB (a.u.)	^{3}A (a.u.)	^{2}B (a.u.)		
UB3LYP-D3/def2svp-CPCM(benzene)	-3601.054828	-3600.438857	-0.511922	N/A	65.3
UB3LYP-D3/def2tzvpp-CPCM(benzene)//UB3LYP-D3/def2svp-CPCM(benzene)	-3602.800133	-3602.181679	-0.51282032	N/A	66.3
UB3LYP-D3/6-311+g(d,p)-CPCM(benzene)//UB3LYP-D3/def2svp-CPCM(benzene)	-3602.583157	-3601.966322	-0.512822064	N/A	65.3
UB3LYP-D3/def2svp	-3601.049714	-3600.433991	-0.511913	0.955850715	66.1
UB3LYP-D3/def2tzvpp//UB3LYP-D3/def2svp	-3602.794977	-3602.176755	-0.512808224	0.000281452	66.3
UM06L-D3/def2tzvpp//UB3LYP-D3/def2svp	-3602.410832	-3601.798824	-0.514069617	0.000503327	61.8
UPBE0-D3/def2tzvpp//UB3LYP-D3/def2svp	-3600.72493	-3600.111203	-0.511689723	0.000252988	64.2

NTO Analysis

Electronic transitions were calculated from TD-DFT single point calculations on optimized geometries. To better visualize the orbitals involved in the electronic transitions, the Natural Transition Orbitals (NTOs) were calculated for every major transition. All orbital images were generated using GaussView6 with an isovalue of 0.05.

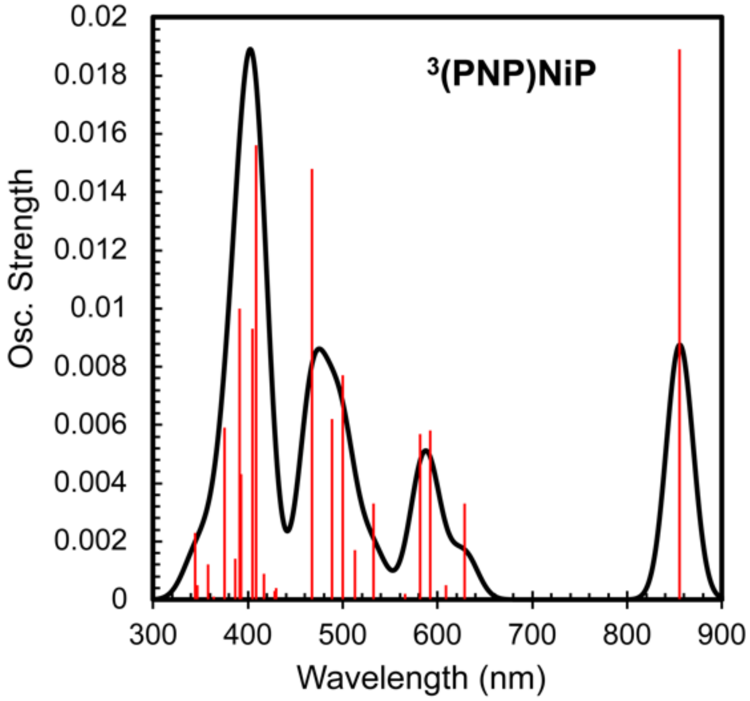

Figure S33. Simulated UV-vis spectrum of compound **2** in the triplet (lowest energy) spin state.

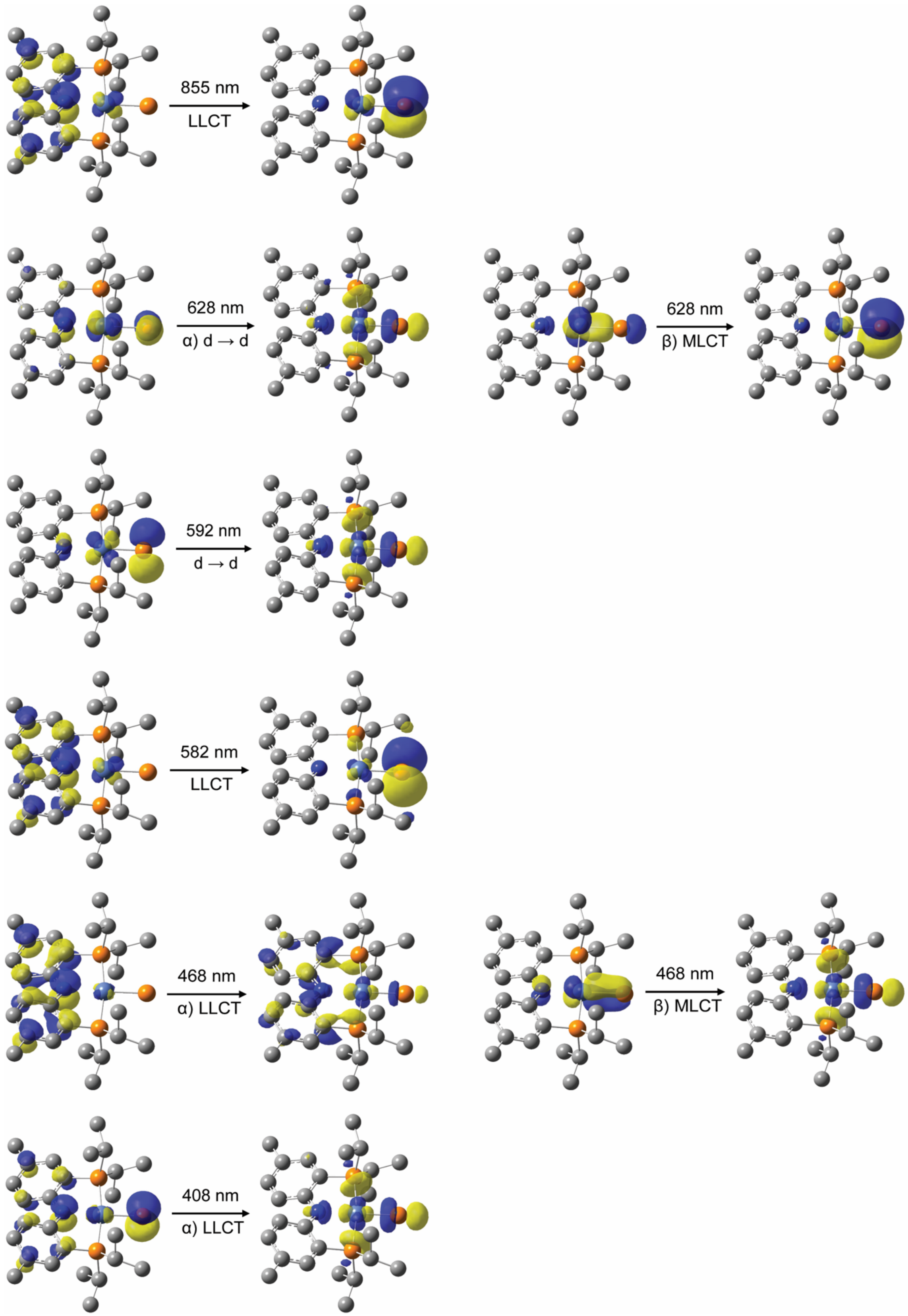

Figure S34. NTO analysis of compound **2** calculated with a triplet spin state.

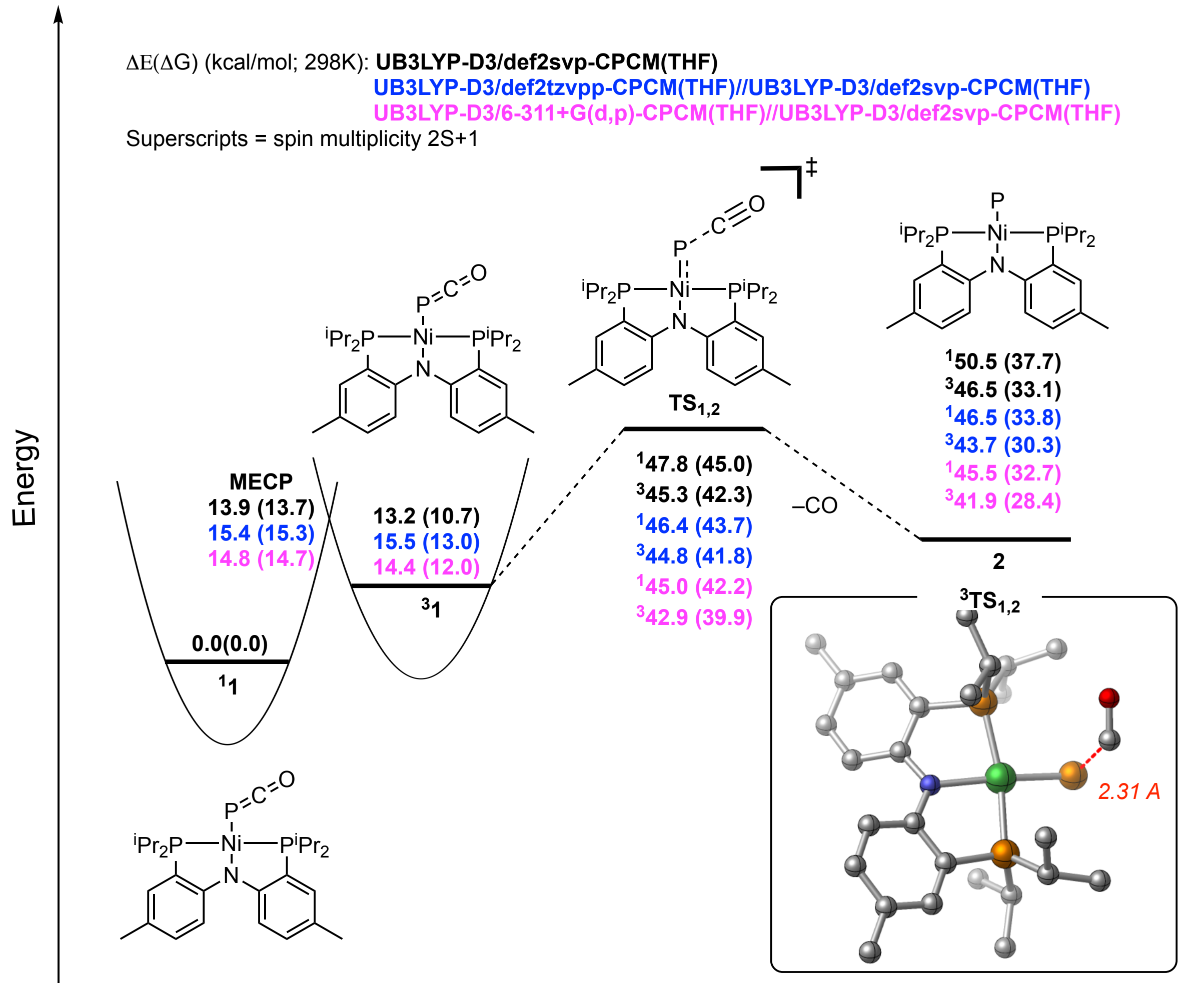

Figure S35. Computed potential energy surface for the photochemical conversion of **1** to **2** at different levels of theory. Calculated energies and Gibbs free energies (parenthesis) are given in kcal mol^{-1}. MECP = minimum energy crossing point.

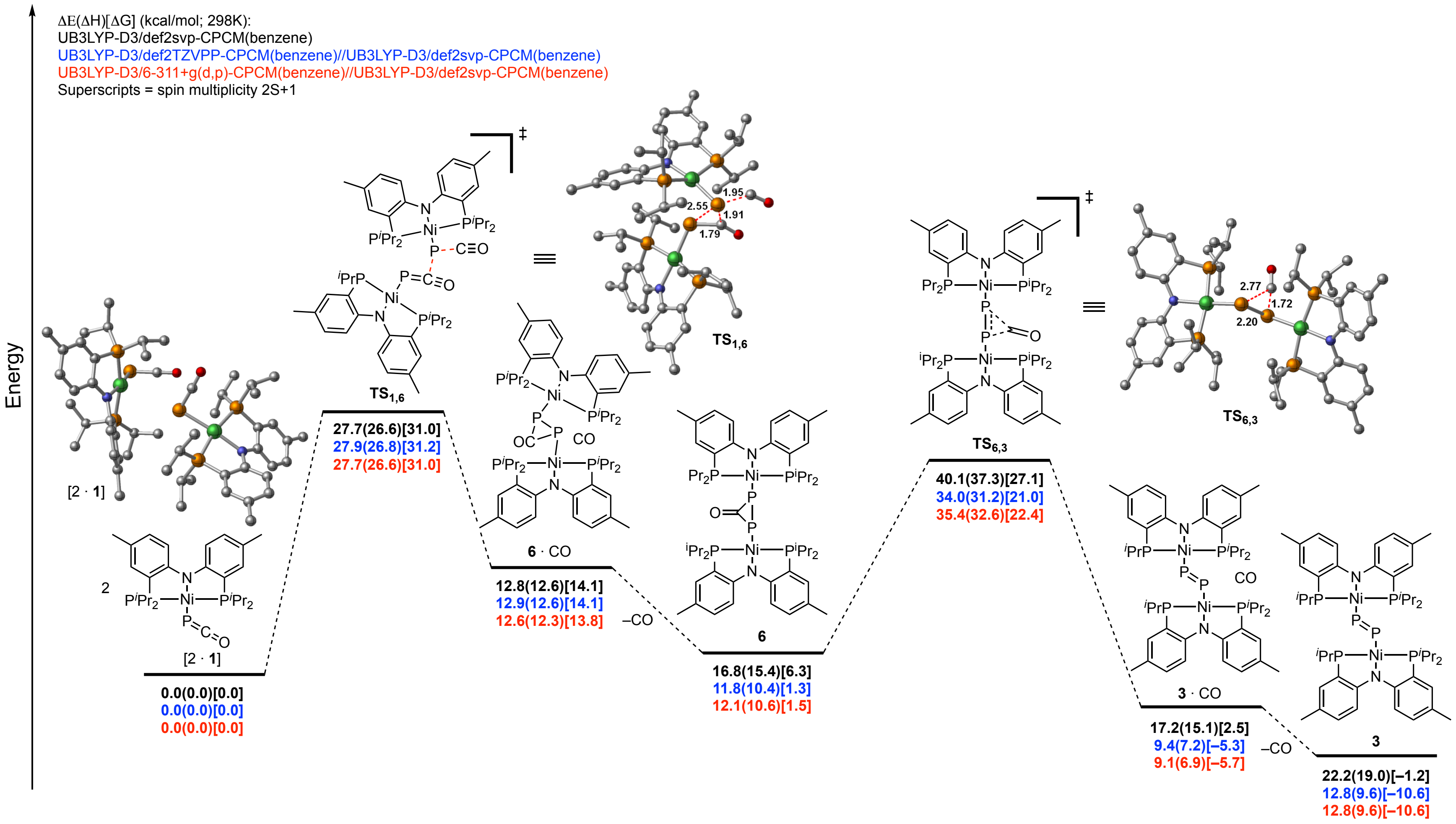

Figure S36. Computed potential energy surface for the thermal conversion of **1** to **3** via [2+1] cycloaddition calculated at various levels of theory. Calculated energies, enthalpy [brackets] and Gibbs free energies (parentheses) are given in kcal mol^{-1}. All structures are singlets.

Table S6. Cartesian coordinates (xyz format) and energies of all the structures involved in each reaction mechanism studied calculated at the CPCM(benzene) uB3LYP-d3/def2-svp level of theory.

^{1}**1**

E(scf) = –3714.27107875 a.u.

ν_{min} = 15.6384 cm^{-1}

Ni	2.902874	2.770797	7.118358
P	2.617348	2.536445	4.934798
P	3.455487	2.619228	9.268990
P	2.394479	5.076331	7.049564
N	3.044152	0.795593	7.145241
C	3.927158	-1.164186	5.880378
H	4.205210	-1.693280	6.791393
C	5.288550	2.859447	9.540584
H	5.437107	3.944307	9.407008
C	3.730288	3.459729	3.750778
H	3.730379	2.851015	2.830997
C	2.921110	0.255792	10.812956
H	3.105323	0.838960	11.719379
C	4.193158	-1.757749	4.650826
H	4.655322	-2.750150	4.638530
O	5.131130	5.652868	7.490120
C	6.058356	2.133138	8.428249
H	5.875314	1.046833	8.460285
H	7.140995	2.300848	8.547707
H	5.762995	2.491442	7.430741
C	2.859437	0.134977	8.356270
C	3.035727	4.971766	10.823221
H	4.114469	5.049132	11.026002
H	2.505728	5.418711	11.680572
H	2.807989	5.574133	9.932112
C	2.388634	-1.195433	8.498189
H	2.119909	-1.771681	7.612998
C	2.225578	-1.772055	9.754420
H	1.850118	-2.798648	9.814532
C	3.338392	0.125503	5.964824
C	0.635186	2.261576	2.895135
H	1.303462	2.764030	2.178930
H	-0.402065	2.478816	2.590684
H	0.788300	1.175152	2.801902
C	2.507219	-1.075271	10.943806
C	5.760350	2.439647	10.935832
H	5.243880	2.987250	11.739089
H	6.839809	2.638022	11.041549
H	5.601352	1.361811	11.099010
C	4.175493	-1.801280	2.109301
H	5.169804	-2.277549	2.093250
H	4.127696	-1.091026	1.269615
H	3.436769	-2.597717	1.909794
C	3.909186	-1.119707	3.428817
C	3.243206	4.868895	3.395442
H	2.254260	4.861034	2.912421
H	3.951898	5.331262	2.688363
H	3.182714	5.507983	4.288083
C	3.113343	0.795720	4.730230
C	2.356614	-1.736258	12.291880
H	2.383358	-0.999750	13.109544
H	3.166399	-2.464074	12.476984
H	1.406478	-2.290585	12.366728
C	3.082975	0.858699	9.559092
C	-0.070161	2.010784	5.325741
H	0.138855	0.928839	5.354957
H	-1.121575	2.145744	5.024218
H	0.049037	2.402171	6.347994
C	2.573767	3.520458	10.643488
H	2.825926	2.962599	11.561021
C	0.862462	2.724874	4.337175
H	0.681360	3.811647	4.406977
C	5.155098	3.466520	4.324742
H	5.216866	4.091706	5.228379
H	5.859036	3.876010	3.582055
H	5.493216	2.451861	4.587692
C	3.384680	0.175255	3.502834
H	3.202117	0.719919	2.572434
C	3.993651	5.405494	7.305800
C	1.059477	3.422730	10.413410
H	0.768797	3.970492	9.503242
H	0.518015	3.864991	11.265591
H	0.729053	2.377951	10.305936

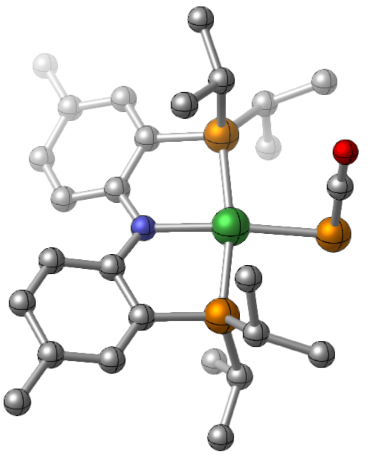

Temperature 298.150 Kelvin. Pressure 1.00000 Atm.

Zero-point correction= 0.611932 (Hartree/Particle)
Thermal correction to Energy= 0.651597
Thermal correction to Enthalpy= 0.652541
Thermal correction to Gibbs Free Energy= 0.540013
Sum of electronic and zero-point Energies= –3713.659146
Sum of electronic and thermal Energies= –3713.619482
Sum of electronic and thermal Enthalpies= –3713.618538
Sum of electronic and thermal Free Energies= –3713.731066

UB3LYP-D3/def2TZVPP-CPCM(benzene)//UB3LYP-D3/def2svp-CPCM(benzene)
E(scf) = –3716.1468 a.u.
UB3LYP-D3/6-311+g(d,p)-CPCM(benzene)//UB3LYP-D3/def2svp-CPCM(benzene)
E(scf) = –3715.9151 a.u.

[2 · **1**]
E(scf) = –7428.56044837 a.u.
ν_{min} = 12.0643 cm^{-1}

Ni	2.030749	9.357037	7.918529
Ni	3.119386	12.242491	13.529915
P	3.086281	10.384931	14.749622
P	3.831092	8.218684	8.553929
P	-0.031469	9.952801	7.309507
P	3.331310	14.287716	12.678560
P	3.208657	10.695238	6.373500
P	2.163819	11.166919	11.640110
N	1.091785	8.003511	9.007966
C	3.817939	12.450886	16.343463
C	-0.884261	8.382572	7.676106
C	3.361523	11.102895	16.401001
N	4.045212	13.017562	15.096781
C	-0.706994	6.285574	8.866916
H	-0.155404	5.582872	9.491677
C	6.777596	15.562482	15.424365
H	7.627904	15.783638	16.077628
C	1.758434	7.461852	10.100719
C	4.662916	14.921145	13.746752
C	-4.055665	6.327587	7.003864
H	-4.797682	6.260175	7.818700
H	-4.013815	5.333018	6.528589
H	-4.445633	7.038914	6.259894
C	5.983608	14.457165	15.716249
H	6.236912	13.832777	16.573348
C	3.288101	11.136111	18.848258
C	-0.143224	7.551006	8.563433
C	-1.946366	5.909606	8.356315
H	-2.337634	4.918588	8.608466
C	-2.702992	6.751866	7.521497

C	3.940063	13.131722	17.582589	H	1.210081	15.192511	12.041662
H	4.215441	14.186402	17.593977	C	3.867862	14.346152	10.899170
C	-2.138265	7.991256	7.193585	H	3.135328	13.668535	10.432922
H	-2.693239	8.663312	6.535441	C	3.321498	11.984502	7.398377
C	4.877539	14.113278	14.897593	C	0.892372	12.218619	11.718810
C	3.681309	12.486079	18.787484	O	3.427422	12.933889	8.087874
H	3.778104	13.055120	19.717832	O	-0.026445	12.957338	11.737678
C	3.126513	10.468756	17.628512	C	-1.619049	10.980067	5.106397
H	2.802932	9.426476	17.640462	H	-1.597819	11.242626	4.035586
C	1.133267	6.948891	11.262285	H	-2.446704	10.269069	5.250158
H	0.046954	6.989113	11.350119	H	-1.857143	11.899099	5.661988
C	3.291664	6.364541	12.264719	C	0.126256	9.184630	4.641353
C	1.886639	6.412770	12.304623	H	0.169911	9.480252	3.580708
H	1.364079	6.024178	13.183547	H	1.114540	8.790010	4.922502
C	3.046712	10.451856	20.171496	H	-0.603682	8.366186	4.740427
H	3.987113	10.315795	20.734033	C	-0.056053	12.530571	8.368527
H	2.595945	9.456791	20.036239	H	0.908111	12.335764	8.861037
H	2.373208	11.041914	20.815539	H	0.157774	12.927750	7.363409
C	5.467391	16.036162	13.479655	H	-0.573053	13.313209	8.945370
H	5.266446	16.640501	12.592385	C	-1.246763	10.705024	9.708666
C	3.918296	6.923772	11.141519	H	-0.350557	10.304991	10.206827
H	5.008834	6.923161	11.095108	H	-1.653732	11.507066	10.341932
C	7.393902	17.601195	14.038523	H	-1.986728	9.894203	9.649902
H	7.206530	18.404685	14.772600	C	3.001469	6.518794	6.473766
H	7.199253	18.017004	13.038125	H	2.120029	6.233594	7.068893
H	8.467684	17.357614	14.099918	H	2.725451	7.399790	5.876129
C	6.534745	16.391345	14.313225	H	3.230567	5.693034	5.780134
C	4.089857	5.701056	13.360014	C	4.683962	5.527093	8.081062
H	4.144918	4.608440	13.208632	H	3.871026	5.094919	8.684773
H	3.640247	5.862971	14.351746	H	4.985251	4.776526	7.331561
H	5.123363	6.077906	13.394885	H	5.539139	5.696198	8.750168
C	3.181552	7.468187	10.085262	C	6.687112	8.218889	9.101749
C	-0.910002	11.257146	8.317455	H	6.868054	7.504416	8.285894
H	-1.855159	11.471537	7.791369	H	7.574234	8.870413	9.168201
C	-0.263161	10.389050	5.508244	H	6.628418	7.656659	10.045794
H	0.507911	11.166533	5.367911	C	5.257136	10.143181	9.953234
C	5.447035	9.088068	8.861223	H	4.419781	10.817161	9.727301
H	5.579668	9.617021	7.900746	H	5.055988	9.676036	10.928666
C	4.212655	6.803406	7.373893	H	6.167434	10.755114	10.051472
H	5.030159	7.200634	6.746586	C	5.242943	13.677224	10.784672
C	4.494870	9.206816	14.395985	H	5.269726	12.726038	11.339013
H	4.093370	8.543672	13.610634	H	5.454348	13.457027	9.727692
C	1.484621	9.433915	14.715589	H	6.040791	14.324006	11.181884
H	1.398480	9.204314	13.638615	C	3.802202	15.680849	10.153888
C	1.842221	15.395143	12.922966	H	4.518594	16.420216	10.544639

H	4.049474	15.500824	9.095240	H	5.372772	10.575366	12.921993
H	2.795220	16.122583	10.182615	C	4.946140	8.374124	15.601277
C	2.173858	16.890486	12.998095	H	4.135341	7.777463	16.040793
H	2.714716	17.257203	12.115191	H	5.743144	7.677679	15.293857
H	1.240355	17.471230	13.083359	H	5.352317	9.022734	16.392755
H	2.788181	17.110686	13.884927	C	0.345513	10.391709	15.089353
C	1.087729	14.936237	14.181016	H	0.374106	11.304364	14.474096
H	1.729025	15.005373	15.074614	H	-0.628405	9.902875	14.925436
H	0.203667	15.573966	14.344934	H	0.405442	10.689943	16.148364
H	0.744459	13.896001	14.092521	C	1.410492	8.113297	15.486342
C	5.670270	10.008601	13.815608	H	0.441465	7.629103	15.279922
H	6.056335	10.732827	14.550862	H	2.195551	7.410298	15.178565
H	6.493714	9.330760	13.536331	H	1.476759	8.258090	16.574745

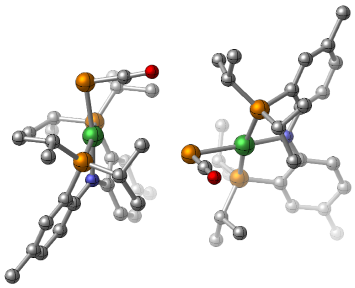

Temperature 298.150 Kelvin. Pressure 1.00000 Atm.

Zero-point correction= 1.226272 (Hartree/Particle)
Thermal correction to Energy= 1.307098
Thermal correction to Enthalpy= 1.308042
Thermal correction to Gibbs Free Energy= 1.107028
Sum of electronic and zero-point Energies= –7427.334177
Sum of electronic and thermal Energies= –7427.253351
Sum of electronic and thermal Enthalpies= –7427.252406
Sum of electronic and thermal Free Energies= –7427.453421

UB3LYP-D3/def2TZVPP-CPCM(benzene)//UB3LYP-D3/def2svp-CPCM(benzene)
E(scf) = –7432.3032 a.u.
UB3LYP-D3/6-311+g(d,p)-CPCM(benzene)//UB3LYP-D3/def2svp-CPCM(benzene)
E(scf) = –7431.8415 a.u.

^{1}2
E(scf) = –3600.96515872 a.u.
ν_{min} = 13.1599 cm^{-1}

Ni	8.879513	6.775859	17.758214	H	8.345519	4.224478	14.178046
P	8.964155	5.113383	16.329460	C	5.706731	2.547125	18.300820
P	8.791788	8.114121	19.491633	H	4.889589	2.007156	18.790175
P	9.649085	8.234773	16.456511	C	7.097328	2.936466	16.394999
N	8.025622	5.415231	18.983049	H	7.390885	2.697196	15.369133
C	7.721158	3.997535	17.063041	C	6.087905	2.176156	16.998203
C	8.500255	5.257986	14.531104	C	8.131814	5.604500	20.349005

C	8.467327	6.907148	20.820667	H	4.330287	1.066199	16.370570
C	7.372871	4.341129	18.401211	C	7.174436	6.021848	14.412927
C	10.614258	2.895182	15.638079	H	6.375371	5.543953	15.000186
H	9.890237	2.186871	16.071433	H	6.850847	6.057321	13.359888
H	11.612971	2.432182	15.701256	H	7.291258	7.056114	14.774103
H	10.374748	3.019447	14.570938	C	11.018366	4.041897	17.870141
C	10.605885	4.222158	16.402539	H	11.055511	5.004859	18.401412
H	11.306176	4.931708	15.928635	H	12.013869	3.572424	17.927092
C	9.609494	5.895868	13.686425	H	10.304674	3.394866	18.405002
H	9.843141	6.915099	14.035977	C	10.294751	9.058996	20.061545
H	9.279123	5.969951	12.637270	H	10.096689	9.327078	21.112963
H	10.540450	5.309247	13.700784	C	8.608371	7.175305	22.188864
C	8.020073	4.591128	21.338915	H	8.866058	8.186467	22.515948
H	7.846445	3.558520	21.037608	C	6.319085	3.591641	18.987184
C	7.359145	9.308324	19.448574	H	5.953326	3.857621	19.978673
H	7.652900	10.029514	18.665977	C	8.448792	6.178387	23.158281
C	8.172297	4.879866	22.691221	C	8.576114	6.468924	24.633563
H	8.087883	4.061376	23.413572	H	7.596175	6.423752	25.140970
C	11.510943	8.124077	20.011923	H	8.994066	7.471186	24.814225
H	11.343045	7.205732	20.595644	H	9.228764	5.735564	25.135995
H	12.398464	8.632998	20.421912	C	10.530419	10.347364	19.266002
H	11.728072	7.832510	18.971711	H	10.687483	10.131565	18.196157
C	6.106180	8.562051	18.970491	H	11.435648	10.852738	19.641166
H	6.267450	8.103250	17.982989	H	9.693774	11.056814	19.353634
H	5.255317	9.258571	18.895851	C	7.123254	10.045544	20.769743
H	5.829556	7.760210	19.674272	H	6.832154	9.343557	21.566858
C	5.429063	1.017644	16.290332	H	6.303351	10.773170	20.651181
H	5.738074	0.050101	16.724031	H	8.011570	10.600219	21.109365
H	5.688103	0.996300	15.220633				

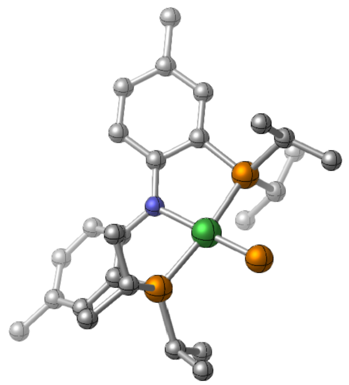

Temperature 298.150 Kelvin. Pressure 1.00000 Atm.

Zero-point correction= 0.602964 (Hartree/Particle)
Thermal correction to Energy= 0.640341
Thermal correction to Enthalpy= 0.641285
Thermal correction to Gibbs Free Energy= 0.533671

Sum of electronic and zero-point Energies= –3600.362195
Sum of electronic and thermal Energies= –3600.324818
Sum of electronic and thermal Enthalpies= –3600.323874
Sum of electronic and thermal Free Energies= –3600.431488

UB3LYP-D3/def2TZVPP-CPCM(benzene)//UB3LYP-D3/def2svp-CPCM(benzene)
E(scf) = –3602.7098 a.u.
UB3LYP-D3/6-311+g(d,p)-CPCM(benzene)//UB3LYP-D3/def2svp-CPCM(benzene)
E(scf) = –3602.4932 a.u.

[**3** · CO]
E(scf) = –7315.30747056 a.u.
ν_{min} = 9.1791 cm^{-1}

Ni	2.123089	8.276182	10.235800
Ni	2.916382	12.417259	13.752344
P	3.744050	11.180398	15.363724
P	2.932686	6.651411	11.467124
P	1.093812	9.655640	8.866720
P	2.119069	13.968098	12.402458
P	3.135729	9.908568	11.379958
P	1.854184	10.714188	12.758723
N	1.297021	6.807592	9.105607
C	5.045885	9.946910	14.872569
H	4.475364	9.220130	14.271513
C	4.382653	13.777990	15.927920
C	0.476299	8.510341	7.594371
C	4.488480	12.450534	16.437931
N	3.899369	13.953995	14.641682
C	6.046531	10.644015	13.941870
H	5.538993	11.065329	13.060844
H	6.802106	9.923414	13.589085
H	6.571063	11.460859	14.464229
C	0.577842	6.240686	6.781108
H	0.908525	5.207273	6.872912
C	5.121391	17.277390	13.394703
H	5.929897	17.988729	13.592813
C	1.263903	5.524219	9.621927
C	3.168626	15.387984	12.840807
C	-1.111041	8.393734	4.098221
H	-0.623245	7.955070	3.211984
H	-1.115597	9.487769	3.974295
H	-2.163070	8.057017	4.082699
C	5.075505	16.100395	14.134480
H	5.856168	15.913183	14.870784
C	5.354870	13.229163	18.594336
C	0.777748	7.141168	7.863693
C	-0.000932	6.655128	5.586425
H	-0.130613	5.920149	4.785230
C	-0.416996	7.982424	5.373260
C	4.705082	14.818288	16.844057
H	4.549518	15.857609	16.558640
C	2.377618	6.704282	13.244716
H	2.852894	7.631332	13.602997
C	-0.138507	8.897725	6.394158
H	-0.398086	9.946891	6.240453
C	4.055470	15.131237	13.927343
C	5.178591	14.543233	18.122253
H	5.405634	15.383133	18.787103
C	0.858120	6.915634	13.269288
H	0.580359	7.840655	12.741286
H	0.501863	6.995910	14.308538
H	0.333067	6.071273	12.794248
C	4.971798	12.198413	17.729990
H	5.031341	11.167275	18.088758
C	0.386060	4.481486	9.212108
H	-0.393140	4.687422	8.479788
C	1.454196	2.851547	10.699908
C	0.360840	14.493300	12.738403
H	0.223681	15.452072	12.207823
C	2.539952	10.326185	16.512907
H	3.090863	10.161558	17.453935
C	0.489007	3.197343	9.734026
H	-0.203742	2.429263	9.375182
C	5.912293	12.956167	19.969538
H	7.003322	13.125733	20.005418

H	5.732631	11.915163	20.279943
H	5.462159	13.617790	20.728137
C	0.206079	14.733515	14.246699
H	0.356477	13.796167	14.806591
H	-0.805427	15.109405	14.471454
H	0.937447	15.465727	14.621318
C	3.212605	16.596289	12.132091
H	2.484374	16.776935	11.338042
C	2.288771	13.691241	10.566120
H	1.792038	12.714024	10.448837
C	1.612187	14.692514	9.624324
H	0.558437	14.877215	9.880338
H	1.636396	14.295921	8.595265
H	2.136510	15.659146	9.609661
C	5.738128	9.220078	16.027896
H	6.370943	9.908999	16.608438
H	6.392796	8.427050	15.628829
H	5.025231	8.745030	16.719433
C	2.245579	3.895168	11.192841
H	2.968584	3.677354	11.983173
C	4.200514	18.893367	11.660108
H	3.569058	18.864150	10.758637
H	5.223976	19.158899	11.347180
H	3.834662	19.723041	12.291051
C	-0.657475	13.471869	12.219772
H	-0.590910	13.325019	11.132285
H	-1.680987	13.813727	12.445852
H	-0.513904	12.490224	12.698738
C	-1.092151	11.588424	8.882621
H	-1.499085	11.192076	7.938941
H	-0.455109	12.454022	8.651138
H	-1.943778	11.960254	9.476466
C	4.765150	6.306845	11.443988
H	4.881144	5.261569	11.780208
C	3.776669	13.504255	10.241297
H	4.342101	14.429940	10.430010
H	3.906369	13.240962	9.178800
H	4.215050	12.694517	10.844761
C	5.558905	7.229421	12.375282
H	6.635487	7.005970	12.293468
H	5.276150	7.108289	13.431612

H	5.414077	8.286985	12.103758
C	-0.345851	10.516670	9.679529
H	0.131157	10.986471	10.557359
C	4.171123	17.579416	12.401329
C	1.611742	1.430644	11.179983
H	0.634670	0.945071	11.337792
H	2.170997	1.382352	12.127234
H	2.160171	0.818403	10.442252
C	2.040290	8.978182	15.990350
H	1.517705	9.095058	15.029274
H	1.327198	8.541021	16.708938
H	2.855162	8.252561	15.852246
C	1.377925	11.292167	16.785749
H	1.733762	12.266653	17.154418
H	0.696544	10.867661	17.541084
H	0.800694	11.469010	15.863648
C	2.809862	5.540308	14.140147
H	2.259769	4.620095	13.893601
H	2.585412	5.783036	15.192495
H	3.887168	5.324543	14.072612
C	5.246645	6.419450	9.990088
H	5.144689	7.456890	9.631808
H	4.667290	5.773016	9.314427
H	6.308526	6.133396	9.914650
C	2.134006	5.204577	10.707443
C	-1.291703	9.423695	10.200497
H	-2.044419	9.866176	10.872671
H	-0.742404	8.649860	10.759156
H	-1.819228	8.926288	9.370777
C	2.233009	10.871908	8.016481
H	2.461428	11.577086	8.829983
C	1.710225	11.656548	6.807454
H	1.598702	11.003269	5.929498
H	2.441990	12.438104	6.542052
H	0.748545	12.152222	6.989341
C	3.528893	10.130228	7.650226
H	3.991900	9.662248	8.530972
H	4.255586	10.834140	7.212359
H	3.331879	9.340508	6.906638
C	3.383878	2.853802	8.092511
O	3.520502	3.962203	7.910386

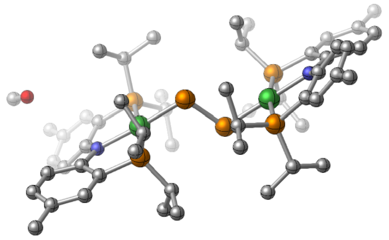

Temperature 298.150 Kelvin. Pressure 1.00000 Atm.

Zero-point correction= 1.215660 (Hartree/Particle)
Thermal correction to Energy= 1.295177
Thermal correction to Enthalpy= 1.296121
Thermal correction to Gibbs Free Energy= 1.097586
Sum of electronic and zero-point Energies= –7314.091810
Sum of electronic and thermal Energies= –7314.012294
Sum of electronic and thermal Enthalpies= –7314.011350
Sum of electronic and thermal Free Energies= –7314.209885

UB3LYP-D3/def2TZVPP-CPCM(benzene)//UB3LYP-D3/def2svp-CPCM(benzene)
E(scf) = –7318.9254 a.u.
UB3LYP-D3/6-311+g(d,p)-CPCM(benzene)//UB3LYP-D3/def2svp-CPCM(benzene)
E(scf) = –7318.4776 a.u.

^{1}3
E(scf) = –7202.07406188 a.u.
ν_{min} = 8.6871 cm^{-1}

Ni	2.040489	8.332875	10.168329	H	0.685965	5.317491	6.797943
Ni	2.956609	12.431242	13.708637	C	5.221691	17.262806	13.349110
P	3.800439	11.168684	15.291405	H	6.044284	17.960706	13.536754
P	2.864491	6.690314	11.365654	C	1.150808	5.591836	9.542635
P	0.982829	9.737692	8.849198	C	3.230384	15.406803	12.819564
P	2.149265	14.006724	12.393989	C	-1.392795	8.565072	4.143051
P	3.094997	9.945023	11.304222	H	-0.942569	8.133091	3.233950
P	1.854725	10.750688	12.720254	H	-1.388585	9.660711	4.034017
N	1.176188	6.883109	9.040805	H	-2.448718	8.240879	4.161043
C	5.076384	9.924848	14.760945	C	5.173046	16.080628	14.080439
H	4.484062	9.211883	14.164507	H	5.965147	15.876105	14.799744
C	4.483103	13.752285	15.867359	C	5.500337	13.165431	18.508712
C	0.318153	8.614267	7.582209	C	0.616712	7.238584	7.822402
C	4.581739	12.418763	16.362732	C	-0.248985	6.794261	5.566622
N	3.976682	13.946766	14.592792	H	-0.415490	6.071636	4.760986
C	6.065976	10.619246	13.816467	C	-0.658262	8.128252	5.386512
H	5.545502	11.055339	12.950250	C	4.837339	14.779550	16.786407
H	6.805264	9.893215	13.441089	H	4.689990	15.823465	16.513609
H	6.611091	11.424494	14.335736	C	2.345982	6.734630	13.154896
C	0.367924	6.356938	6.733932	H	2.833350	7.657246	13.508361

C	-0.334631	9.025127	6.410312
H	-0.589013	10.078878	6.281583
C	4.135180	15.128062	13.885676
C	5.332205	14.486136	18.052384
H	5.583377	15.316621	18.720379
C	0.828486	6.953798	13.212793
H	0.544629	7.884664	12.698560
H	0.494528	7.027496	14.259913
H	0.288900	6.116274	12.741943
C	5.086988	12.147995	17.642514
H	5.139895	11.112708	17.990181
C	0.261970	4.555017	9.140448
H	-0.529004	4.768549	8.423331
C	1.330475	2.912264	10.614427
C	0.408200	14.551523	12.784592
H	0.266805	15.514853	12.263488
C	2.608754	10.317486	16.455580
H	3.176186	10.136178	17.383622
C	0.358801	3.268799	9.660526
H	-0.350224	2.510279	9.312704
C	6.080883	12.872172	19.870107
H	7.174459	13.027751	19.886250
H	5.894105	11.830537	20.174106
H	5.654002	13.531980	20.643593
C	0.300245	14.784801	14.297979
H	0.459716	13.843377	14.848352
H	-0.701073	15.167569	14.554517
H	1.048315	15.509169	14.654303
C	3.278466	16.619848	12.119329
H	2.537592	16.817620	11.341256
C	2.264228	13.747749	10.550824
H	1.753911	12.777062	10.437396
C	1.571699	14.765428	9.638675
H	0.527085	14.956637	9.925468
H	1.563984	14.379930	8.605161
H	2.104196	15.727485	9.619838
C	5.783479	9.177215	15.893860
H	6.436665	9.852009	16.468521
H	6.419996	8.380780	15.472793
H	5.079317	8.703135	16.594973
C	2.145534	3.944970	11.089473
H	2.878696	3.719187	11.868191
C	4.291324	18.905517	11.644986
H	3.642312	18.892460	10.755682
H	5.312383	19.158496	11.314319
H	3.949903	19.735554	12.289003
C	-0.636270	13.544596	12.290230
H	-0.601925	13.402214	11.200673
H	-1.648991	13.897078	12.546560
H	-0.490620	12.558985	12.760335
C	-1.181517	11.692384	8.956320
H	-1.622272	11.315703	8.019809
H	-0.540725	12.553396	8.718166
H	-2.009853	12.065698	9.581354
C	4.692460	6.325846	11.306750
H	4.803614	5.281431	11.647124
C	3.740345	13.548491	10.182476
H	4.320471	14.466576	10.363340
H	3.837395	13.293356	9.114536
H	4.187025	12.728976	10.766391
C	5.515776	7.244012	12.216325
H	6.587963	7.008411	12.113464
H	5.253607	7.129561	13.278671
H	5.376485	8.302194	11.944428
C	-0.425115	10.599819	9.714583
H	0.080922	11.050700	10.586175
C	4.256828	17.586461	12.376882
C	1.467267	1.492173	11.105348
H	0.483801	1.039471	11.314464
H	2.066462	1.438472	12.027550
H	1.961221	0.849207	10.354962
C	2.081851	8.981354	15.929319
H	1.540990	9.114755	14.980548
H	1.378495	8.545086	16.657976
H	2.884778	8.247581	15.766719
C	1.464701	11.294912	16.761667
H	1.840066	12.260456	17.134469
H	0.792535	10.870357	17.525186
H	0.872196	11.489688	15.852950
C	2.790648	5.564209	14.035764
H	2.231731	4.647550	13.796093
H	2.588002	5.802895	15.093443
H	3.865483	5.344194	13.946352
C	5.145417	6.422116	9.842941
H	5.042254	7.456064	9.474976
H	4.546691	5.771198	9.187045
H	6.203224	6.127401	9.747642
C	2.040434	5.257484	10.607729
C	-1.368341	9.508967	10.244502
H	-2.095906	9.948560	10.945660

H	-0.812606	8.719546	10.774143	H	2.285026	12.536945	6.518159
H	-1.925512	9.031732	9.422388	H	0.602929	12.259894	7.011240
C	2.106046	10.956520	7.981211	C	3.386324	10.211296	7.570220
H	2.361684	11.650778	8.795925	H	3.871165	9.730969	8.432380
C	1.554797	11.758384	6.796334	H	4.104710	10.915660	7.119528
H	1.412512	11.115604	5.915066	H	3.163390	9.431077	6.823925

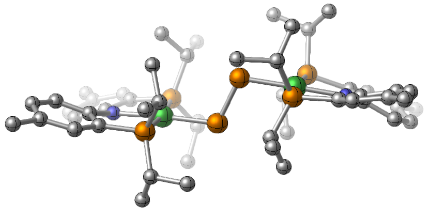

Temperature 298.150 Kelvin. Pressure 1.00000 Atm.

Zero-point correction= 1.209516 (Hartree/Particle)
Thermal correction to Energy= 1.285164
Thermal correction to Enthalpy= 1.286109
Thermal correction to Gibbs Free Energy= 1.097784
Sum of electronic and zero-point Energies= –7200.864546
Sum of electronic and thermal Energies= –7200.788898
Sum of electronic and thermal Enthalpies= –7200.787953
Sum of electronic and thermal Free Energies= –7200.976278

UB3LYP-D3/def2TZVPP-CPCM(benzene)//UB3LYP-D3/def2svp-CPCM(benzene)
E(scf) = –7205.5571 a.u.
UB3LYP-D3/6-311+g(d,p)-CPCM(benzene)//UB3LYP-D3/def2svp-CPCM(benzene)
E(scf) = –7205.1222 a.u.

^{1}5
E(scf) = –3602.22219848 a.u.
ν_{min} = 19.4151 cm^{-1}

Ni	8.663022	6.848070	17.711966	C	8.315993	6.946591	20.788925
P	8.895894	5.158013	16.287022	C	7.365060	4.316370	18.372586
P	8.695854	8.148822	19.469301	C	10.734486	3.066681	15.629756
P	9.028470	8.470434	16.225616	H	10.048913	2.308919	16.041860
N	7.949705	5.440446	18.940763	H	11.761094	2.676840	15.731113
C	7.748278	3.960895	17.048601	H	10.525664	3.169330	14.554099
C	8.427550	5.170256	14.480112	C	10.602616	4.391999	16.385650
H	8.408478	4.111158	14.173034	H	11.258291	5.149325	15.921058
C	5.812946	2.420937	18.306144	C	9.449910	5.901939	13.600401
H	5.022139	1.847701	18.800888	H	9.490527	6.974694	13.837965
C	7.204663	2.840252	16.408579	H	9.155712	5.808008	12.541992
H	7.523825	2.592272	15.392560	H	10.465295	5.488301	13.697533
C	6.239340	2.032992	17.023092	C	7.973199	4.610060	21.293214
C	8.042299	5.633473	20.307733	H	7.859251	3.571107	20.985987

C	7.431005	9.518677	19.519899
H	7.801455	10.238076	18.768972
C	8.088066	4.898246	22.648407
H	8.034965	4.072235	23.365219
C	11.414675	7.795119	19.866219
H	11.141995	6.850734	20.361879
H	12.368894	8.142550	20.294866
H	11.575288	7.583962	18.796537
C	6.089476	8.956936	19.028590
H	6.184468	8.517430	18.023759
H	5.331560	9.756116	18.987679
H	5.718869	8.171127	19.707290
C	5.671308	0.810347	16.345081
H	6.065540	-0.119861	16.791648
H	5.918934	0.790024	15.272593
H	4.573508	0.768450	16.439205
C	7.015575	5.751169	14.319441
H	6.275729	5.194817	14.914606
H	6.707247	5.702701	13.262043
H	6.988612	6.805370	14.635659
C	10.999981	4.248054	17.861557
H	10.915266	5.203905	18.397883
H	12.039153	3.889669	17.942132
H	10.348043	3.523598	18.375620
C	10.330087	8.866203	20.043091
H	10.189098	9.046473	21.121733
C	8.424853	7.211956	22.161856
H	8.643461	8.229133	22.497478
C	6.347819	3.523780	18.965748
H	5.948810	3.798496	19.941863
C	8.291318	6.207013	23.125551
C	8.376924	6.498060	24.603630
H	7.385503	6.436234	25.086719
H	8.772684	7.507395	24.795250
H	9.029046	5.775460	25.121972
C	10.734408	10.189823	19.387211
H	10.946710	10.059043	18.316440
H	11.653056	10.567117	19.866453
H	9.964319	10.969168	19.490669
C	7.297394	10.228184	20.870548
H	6.880363	9.551801	21.632342
H	6.609469	11.084734	20.776113
H	8.258511	10.614388	21.243790
H	10.343244	8.191608	15.730567
H	9.463187	9.625534	16.943586

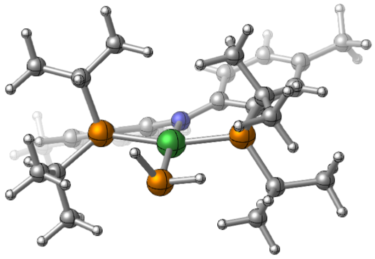

Temperature 298.150 Kelvin. Pressure 1.00000 Atm.

Zero-point correction= 0.620287 (Hartree/Particle)
Thermal correction to Energy= 0.658363
Thermal correction to Enthalpy= 0.659308
Thermal correction to Gibbs Free Energy= 0.551697
Sum of electronic and zero-point Energies= –3601.601911
Sum of electronic and thermal Energies= –3601.563835
Sum of electronic and thermal Enthalpies= –3601.562891
Sum of electronic and thermal Free Energies= –3601.670501

UB3LYP-D3/def2TZVPP-CPCM(benzene)//UB3LYP-D3/def2svp-CPCM(benzene)
E(scf) = –3603.9698 a.u.
UB3LYP-D3/6-311+g(d,p)-CPCM(benzene)//UB3LYP-D3/def2svp-CPCM(benzene)
E(scf) = –3603.7516 a.u.

6 · CO
E(scf) = –7428.53995211 a.u.
ν_{min} = 15.6713 cm^{-1}

Ni	5.484665	3.982801	9.253812	N	3.630590	2.733266	4.307208
P	3.274967	4.179168	9.042616	C	1.321496	0.880199	2.013296
P	7.620022	4.043790	9.801298	H	0.404196	1.063377	1.443883
P	5.629225	2.842502	7.361381	C	3.382295	6.418205	4.575610
O	8.749074	-1.058815	5.865249	H	4.036429	7.286136	4.498095
N	5.175949	4.821308	11.049574	C	3.047424	1.756845	3.524449
C	2.373597	4.533498	13.525256	C	3.126071	-0.545347	2.650835
H	2.224809	4.549367	14.609858	H	3.634385	-1.507804	2.591975
C	9.334433	-0.639149	6.739020	C	1.920027	-0.392365	1.960937
C	8.675404	5.769810	11.830395	C	1.857296	1.921635	2.760224
H	9.675967	5.502521	11.483849	H	1.355878	2.887674	2.739316
C	3.925062	4.675142	11.633323	C	3.063314	3.989042	4.503269
C	1.518442	4.259494	11.310367	C	3.709620	0.496910	3.390255
H	0.683929	4.075568	10.631628	C	3.920129	5.131555	4.471539
C	1.263296	4.334115	12.684475	C	1.291488	-1.524050	1.185333
C	3.662756	4.698624	13.028470	H	0.378884	-1.899560	1.681644
H	4.487248	4.817860	13.730974	H	0.993724	-1.205577	0.172072
C	6.236716	5.465161	11.670470	H	1.982062	-2.375121	1.080734
C	2.810701	4.401999	10.787518	C	2.010686	6.639655	4.786034
C	7.559128	5.159927	11.241153	C	1.186073	5.510834	4.891111
C	-0.130895	4.201217	13.246348	H	0.119876	5.641608	5.099220
H	-0.511729	5.169309	13.617279	C	1.692833	4.217274	4.756879
H	-0.159131	3.500630	14.097478	H	1.022458	3.365153	4.867626
H	-0.840384	3.837786	12.487152	C	1.474261	8.042625	4.930934
C	8.547140	6.728334	12.841671	H	1.936420	8.563085	5.787359
C	7.237549	7.078068	13.220901	H	1.686293	8.652009	4.036020
H	7.091714	7.859070	13.974235	H	0.385336	8.045308	5.088444
C	6.115505	6.476265	12.660702	C	6.678370	-0.173461	3.075308
H	5.126123	6.811902	12.970998	H	7.381116	-0.655384	3.776708
C	9.748448	7.367025	13.494307	C	5.175315	-0.932628	5.545025
H	9.897667	6.991000	14.522019	H	6.110292	-0.823507	6.114595
H	9.635003	8.461330	13.568793	C	5.948069	4.886593	2.369580
H	10.672083	7.160886	12.931925	H	6.984016	4.537495	2.217142
P	7.443926	2.377380	6.059478	C	6.858881	5.792813	5.110769
O	6.924289	0.374828	8.070144	H	6.849871	5.346306	6.119005
C	6.714188	1.370823	7.414794	C	2.798447	5.709867	8.087741
Ni	5.459581	2.514517	4.922700	H	2.693521	5.341981	7.056523
P	5.318980	0.412818	4.246789	C	2.346287	2.738001	8.308703
P	5.673992	4.648126	4.213316	H	2.860715	2.623606	7.336698

C	8.729955	4.825576	8.522123
H	8.413091	4.291015	7.609568
C	8.320277	2.406428	10.371397
H	8.622394	1.894289	9.442161
C	6.273525	-1.186906	1.997174
H	5.577725	-0.734665	1.274046
H	7.170406	-1.506668	1.440355
H	5.800341	-2.089777	2.404186
C	7.396162	1.017521	2.424683
H	6.732585	1.539907	1.717838
H	7.736868	1.751311	3.167888
H	8.272370	0.662487	1.856671
C	4.008174	-0.579482	6.474870
H	4.078668	0.459332	6.825255
H	3.040939	-0.699290	5.959946
H	4.013085	-1.237173	7.358794
C	5.092689	-2.382120	5.057362
H	5.979667	-2.681112	4.480385
H	5.030582	-3.055201	5.929311
H	4.196351	-2.561644	4.444011
C	5.821732	6.330121	1.872077
H	5.949788	6.362931	0.776772
H	4.825384	6.741841	2.100005
H	6.577826	6.996313	2.310928
C	5.009774	3.948671	1.598475
H	5.073829	2.917429	1.969007
H	3.959876	4.267006	1.698160
H	5.265705	3.956752	0.526449
C	6.528800	7.281957	5.264775
H	7.352759	7.780256	5.801537
H	6.412138	7.792143	4.297857
H	5.618040	7.441315	5.854111
C	8.268878	5.594240	4.528878
H	9.018258	6.025499	5.211855
H	8.518657	4.530931	4.395979
H	8.379597	6.100481	3.556968
C	10.241678	4.630455	8.659354
H	10.743308	5.078237	7.784849
H	10.650267	5.120326	9.556525
H	10.518535	3.566436	8.684432
C	8.342450	6.302554	8.372837
H	8.813180	6.724719	7.471307
H	7.252474	6.430793	8.279829
H	8.673883	6.895371	9.238666
C	7.179338	1.604861	11.019089
H	6.751547	2.148335	11.877513
H	6.376452	1.399044	10.298642
H	7.561036	0.636747	11.381601
C	9.521108	2.513277	11.319075
H	9.887001	1.501336	11.559026
H	10.361592	3.076926	10.892594
H	9.232467	2.997455	12.264747
C	0.853292	2.917148	8.016784
H	0.485686	2.029301	7.475524
H	0.254234	3.009551	8.934880
H	0.658886	3.789709	7.379234
C	2.630083	1.487678	9.151755
H	2.164790	1.565016	10.147144
H	2.224075	0.595123	8.650497
H	3.711232	1.330154	9.292342
C	3.961481	6.709881	8.128204
H	4.192072	7.020357	9.159886
H	4.873991	6.268590	7.701370
H	3.709255	7.608777	7.542565
C	1.486266	6.358876	8.536817
H	1.245991	7.201667	7.868333
H	0.635570	5.663985	8.512096
H	1.570850	6.749273	9.563106

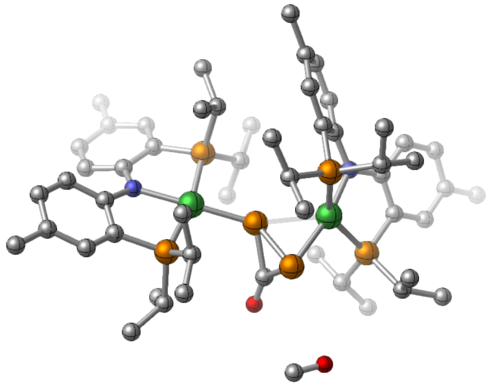

Temperature 298.150 Kelvin. Pressure 1.00000 Atm.

Zero-point correction= 1.225924 (Hartree/Particle)
Thermal correction to Energy= 1.306672
Thermal correction to Enthalpy= 1.307616
Thermal correction to Gibbs Free Energy= 1.109049
Sum of electronic and zero-point Energies= –7427.314028
Sum of electronic and thermal Energies= –7427.233280
Sum of electronic and thermal Enthalpies= –7427.232336
Sum of electronic and thermal Free Energies= –7427.430903

UB3LYP-D3/def2TZVPP-CPCM(benzene)//UB3LYP-D3/def2svp-CPCM(benzene)
E(scf) = –7432.2827 a.u.
UB3LYP-D3/6-311+g(d,p)-CPCM(benzene)//UB3LYP-D3/def2svp-CPCM(benzene)
E(scf) = –7431.8215 a.u.

6
E(scf) = –7315.30814068 a.u.
ν_{min} = 4.6670 cm^{-1}

Ni	5.005285	4.641384	10.007512	C	8.690838	2.341016	13.363345
P	4.102250	6.673363	10.166892	C	8.507571	3.617422	13.928311
P	6.133514	2.767292	10.115511	H	9.138659	3.924309	14.768747
P	3.951679	4.122144	8.110243	C	5.038084	8.004730	9.257772
O	4.207364	6.409350	6.384863	H	4.801672	7.798972	8.200631
N	5.685130	4.979507	11.866366	C	2.276130	6.987218	9.901981
C	4.363231	7.012994	14.733796	H	2.060818	7.901430	10.478816
H	4.359833	6.990880	15.828452	C	7.453587	2.629762	8.802017
C	4.342436	5.250883	6.730401	H	6.876104	2.306244	7.921613
C	7.924962	2.040605	12.232795	C	7.555637	4.510975	13.451454
H	8.087190	1.084582	11.732195	H	7.481242	5.494318	13.913931
C	5.054931	5.953754	12.630197	C	1.874810	7.239827	8.447067
C	3.661826	7.984046	12.669788	H	2.409846	8.088137	7.998375
H	3.098017	8.744855	12.123637	H	0.795026	7.458549	8.402253
C	3.695971	8.055929	14.066546	H	2.067138	6.365041	7.815943
C	5.015732	5.992087	14.050726	C	4.611724	9.431207	9.617255
H	5.477441	5.191693	14.626844	H	3.534874	9.603011	9.466941
C	6.700440	4.177613	12.364538	H	5.152632	10.152260	8.981891
C	5.057523	1.244114	10.104214	H	4.853403	9.666491	10.665740
H	4.588643	1.297748	9.107571	C	1.483806	5.827163	10.521308
C	4.301599	6.958507	11.959320	H	1.658547	4.894179	9.962094
C	6.977633	2.938294	11.714889	H	0.403886	6.046703	10.489869
C	3.039375	9.182332	14.825759	H	1.764985	5.654136	11.571854
H	2.489064	8.810639	15.705821	C	5.750038	-0.112192	10.256428
H	2.329958	9.737874	14.193062	H	6.476469	-0.306716	9.455634
H	3.784752	9.907725	15.198004	H	4.996753	-0.916668	10.217243

H	6.267622	-0.195802	11.224334
C	6.541601	7.787625	9.477104
H	6.809667	7.902441	10.540408
H	7.120515	8.525967	8.898670
H	6.855480	6.781981	9.161632
C	3.955717	1.439454	11.156408
H	4.379517	1.463139	12.173571
H	3.233503	0.608556	11.106400
H	3.408415	2.381037	10.995703
C	9.683352	1.362199	13.940610
H	9.883478	0.531495	13.246346
H	9.315287	0.921342	14.884141
H	10.645356	1.849490	14.170917
C	8.609046	1.654689	9.049699
H	9.240636	1.993896	9.883636
H	9.246343	1.615645	8.151751
H	8.278406	0.629910	9.265370
C	7.993283	4.038616	8.514873
H	7.192911	4.708006	8.170638
H	8.761840	3.994100	7.726276
H	8.454444	4.478182	9.414710
P	5.115608	3.692795	6.156855
Ni	3.817942	2.437826	4.747843
P	5.203959	0.715245	4.916682
P	2.285872	3.975512	4.296895
N	2.808552	1.286486	3.458216
C	3.727655	-1.638018	1.289622
H	3.416354	-2.093089	0.343673
C	-0.105115	3.490777	2.794920
H	-0.360134	4.545216	2.907271
C	3.418508	0.151680	2.942664
C	5.204197	-1.535590	3.160250
H	6.076211	-1.911252	3.701990
C	4.792858	-2.212624	2.008727
C	3.065786	-0.496242	1.725347
H	2.279747	-0.078788	1.098080
C	1.529984	1.672756	3.071318
C	2.913248	5.502302	3.418895
H	3.200538	6.174736	4.244323
C	4.554161	-0.378914	3.617090
C	1.149788	3.034795	3.227267
C	5.464039	-3.479782	1.539980
H	5.656646	-3.458246	0.454518
H	6.425898	-3.641541	2.050725
H	4.835168	-4.366777	1.734819

C	-1.059218	2.630272	2.242357
C	-0.725093	1.264381	2.194679
H	-1.468643	0.544013	1.838507
C	5.165979	-0.176409	6.552134
H	5.697957	0.513839	7.228077
C	6.993522	0.909947	4.394067
H	7.310683	-0.100263	4.087436
C	1.239660	4.475868	5.762202
H	1.994724	4.785437	6.496649
C	0.519089	0.792824	2.598465
H	0.706863	-0.279895	2.571410
C	7.940852	1.397862	5.491415
H	7.945477	0.730543	6.364970
H	8.970705	1.430916	5.098539
H	7.675849	2.410680	5.827541
C	5.868735	-1.535969	6.571620
H	6.911916	-1.476678	6.224425
H	5.881718	-1.935387	7.599078
H	5.337407	-2.263901	5.939796
C	7.025162	1.825425	3.162037
H	6.712958	2.847098	3.430757
H	8.046493	1.875792	2.750651
H	6.354508	1.464028	2.367078
C	1.898878	6.199659	2.505292
H	0.987181	6.509562	3.033990
H	2.356207	7.106292	2.075501
H	1.603623	5.546659	1.669308
C	3.704307	-0.270166	7.012364
H	3.102668	-0.856971	6.299386
H	3.641892	-0.763744	7.995547
H	3.248355	0.728565	7.095988
C	4.183543	5.124786	2.640112
H	3.973046	4.350982	1.882719
H	4.586267	6.008542	2.118593
H	4.964882	4.736291	3.309582
C	-2.390285	3.132199	1.739830
H	-2.613484	4.140360	2.121956
H	-2.410925	3.187506	0.636725
H	-3.214456	2.465044	2.041447
C	0.263966	5.639473	5.578708
H	-0.527703	5.412753	4.849466
H	-0.229048	5.850410	6.542011
H	0.777796	6.561518	5.269836
C	0.563779	3.222216	6.331948
H	1.289140	2.402927	6.462088

H	0.132030	3.450292	7.320005	H	-0.243451	2.862060	5.675343

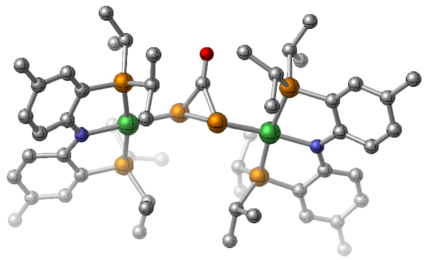

Temperature 298.150 Kelvin. Pressure 1.00000 Atm.

Zero-point correction= 1.218907 (Hartree/Particle)
Thermal correction to Energy= 1.296409
Thermal correction to Enthalpy= 1.297353
Thermal correction to Gibbs Free Energy= 1.104269
Sum of electronic and zero-point Energies= –7314.089234
Sum of electronic and thermal Energies= –7314.011732
Sum of electronic and thermal Enthalpies= –7314.010788
Sum of electronic and thermal Free Energies= –7314.203872

UB3LYP-D3/def2TZVPP-CPCM(benzene)//UB3LYP-D3/def2svp-CPCM(benzene)
E(scf) = –7318.9215 a.u.
UB3LYP-D3/6-311+g(d,p)-CPCM(benzene)//UB3LYP-D3/def2svp-CPCM(benzene)
E(scf) = –7318.472812 a.u.

[1]Int1
E(scf) = –7428.53987403 a.u.
ν_{min} = 3.9016 cm^{-1}

Ni	1.719060	8.106366	10.505009	H	6.726730	17.655352	15.538842
Ni	2.824266	13.312592	12.989662	C	1.831516	5.364063	9.474065
P	2.241327	11.928949	14.638850	C	5.047302	15.444841	12.844531
P	3.681969	7.337559	9.771766	C	-4.545261	6.646611	8.014118
P	-0.404522	8.574207	10.918387	H	-4.664818	6.703924	6.919249
P	4.491947	14.105998	11.744119	H	-5.064768	7.511142	8.455094
P	2.709440	9.788061	11.587734	H	-5.076959	5.737196	8.345569
P	2.098435	11.970322	11.268919	C	5.283026	16.124685	15.147511
N	0.989854	6.455635	9.640537	H	5.086377	15.992325	16.209368
C	2.895340	14.357138	15.684485	C	1.518968	14.002119	18.188675
C	-1.222121	7.409139	9.781705	C	-0.324768	6.466319	9.204711
C	2.229255	13.136574	16.008421	C	-2.185182	5.730206	7.789877
N	3.583376	14.423780	14.479655	H	-2.539439	5.088968	6.976195
C	-0.848478	5.652800	8.164249	C	-3.089865	6.611784	8.411197
H	-0.187921	4.971906	7.627786	C	2.724163	15.424853	16.602463
C	6.232471	17.063741	14.761360	H	3.092339	16.417710	16.350730

C	-2.571028	7.454167	9.400056
H	-3.240648	8.170651	9.877884
C	4.587787	15.328445	14.192828
C	2.058135	15.243594	17.811555
H	1.949417	16.101007	18.483475
C	1.603893	12.965622	17.250352
H	1.161392	12.001087	17.499853
C	1.412086	4.011386	9.383630
H	0.352227	3.768125	9.459012
C	3.719907	3.212599	9.177306
C	2.333690	2.979083	9.237357
H	1.963752	1.950127	9.182175
C	0.868247	13.804801	19.535706
H	0.209745	14.650346	19.794329
H	1.621060	13.728459	20.340369
H	0.264192	12.884803	19.563441
C	5.990317	16.417759	12.479619
H	6.294773	16.494573	11.433905
C	4.143506	4.540136	9.312904
H	5.216566	4.749588	9.307418
C	7.555380	18.351073	13.009526
H	8.412516	18.400959	13.701124
H	7.082330	19.349436	13.014230
H	7.948660	18.180630	11.995275
C	6.579512	17.274124	13.413856
C	4.696074	2.078129	8.984115
H	4.479615	1.237496	9.664246
H	5.732812	2.400455	9.165769
H	4.650497	1.676633	7.956318
C	3.234315	5.594119	9.470446
C	-0.983059	8.229773	12.669020
H	-0.946487	9.218953	13.152912
C	-0.945456	10.299990	10.491694
H	-0.261894	10.900109	11.115835
C	5.222265	7.238152	10.825081
H	5.849963	6.476204	10.333763
C	4.177362	8.076439	8.132583
H	4.488157	9.099922	8.399494
C	3.541145	10.625185	15.020877
H	3.880065	10.360263	14.005915
C	0.565056	11.097910	14.507587
H	0.789766	10.299715	13.779798
C	4.046712	14.767092	10.045371
H	3.453757	13.926739	9.642360
C	5.995011	12.996006	11.498201
H	5.767328	12.475223	10.556316
C	3.428669	10.991698	10.446023
C	1.383608	14.633789	12.935826
O	4.254300	11.110611	9.561318
O	0.789119	15.531790	13.320762
C	2.938278	8.167626	7.230762
H	2.134584	8.750798	7.705428
H	3.200957	8.655275	6.277906
H	2.532998	7.168351	7.002251
C	5.331127	7.336780	7.449325
H	5.047660	6.302546	7.196395
H	5.597851	7.848979	6.509875
H	6.237807	7.300413	8.072661
C	4.842404	6.726227	12.222061
H	4.277940	5.782122	12.172647
H	5.751766	6.548404	12.819282
H	4.225305	7.467285	12.754432
C	6.024938	8.542711	10.890335
H	6.322438	8.908391	9.897466
H	5.455234	9.341359	11.382132
H	6.943710	8.373263	11.476181
C	-0.582603	10.585744	9.030001
H	-0.728823	11.654916	8.808816
H	0.473736	10.344428	8.831518
H	-1.206556	9.997756	8.338180
C	-2.380079	10.711974	10.832856
H	-3.121376	10.208495	10.195040
H	-2.636565	10.516288	11.884163
H	-2.491398	11.796623	10.668019
C	0.026372	7.304986	13.364031
H	0.075296	6.325685	12.860764
H	1.040714	7.731376	13.349969
H	-0.265488	7.136268	14.413617
C	-2.409517	7.680414	12.772902
H	-2.679642	7.552087	13.834313
H	-3.155825	8.346060	12.316830
H	-2.490849	6.698925	12.281269
C	6.054446	11.959279	12.622440
H	5.100981	11.427795	12.729929
H	6.843686	11.218459	12.416450
H	6.286177	12.438384	13.585838
C	7.343859	13.714889	11.371446
H	7.366834	14.465645	10.571499
H	7.617318	14.216392	12.311943
H	8.126930	12.970573	11.148603

C	5.194306	15.023401	9.061171	C	3.098505	9.324308	15.694478
H	5.814541	14.130765	8.900972	H	3.962310	8.640277	15.750102
H	4.771370	15.306161	8.082762	H	2.745563	9.488512	16.724904
H	5.844800	15.851088	9.383499	H	2.310574	8.806437	15.128772
C	3.133525	15.991967	10.171377	C	-0.455352	12.058118	13.879386
H	2.758612	16.283440	9.176787	H	-0.142250	12.410824	12.888717
H	2.262887	15.794410	10.811731	H	-1.427858	11.551695	13.768436
H	3.676449	16.851450	10.595790	H	-0.613161	12.939570	14.521149
C	4.698024	11.288048	15.778982	C	-0.050902	10.457696	15.762254
H	4.429012	11.460806	16.832771	H	-0.870635	9.785674	15.457206
H	5.586131	10.635911	15.752229	H	0.654018	9.866047	16.356244
H	4.969773	12.258542	15.341821	H	-0.492509	11.224908	16.414224

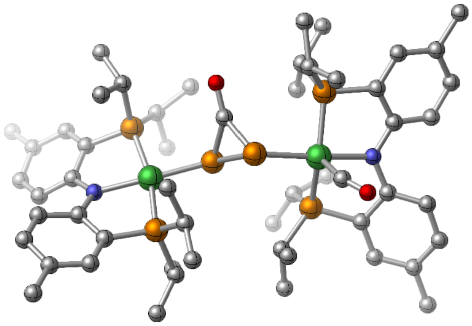

Temperature 298.150 Kelvin. Pressure 1.00000 Atm.

Zero-point correction= 1.226311 (Hartree/Particle)
Thermal correction to Energy= 1.306647
Thermal correction to Enthalpy= 1.307591
Thermal correction to Gibbs Free Energy= 1.107137
Sum of electronic and zero-point Energies= –7427.313563
Sum of electronic and thermal Energies= –7427.233227
Sum of electronic and thermal Enthalpies= –7427.232283
Sum of electronic and thermal Free Energies= –7427.432737

UB3LYP-D3/def2TZVPP-CPCM(benzene)//UB3LYP-D3/def2svp-CPCM(benzene)
E(scf) = –7432.28 a.u.
UB3LYP-D3/6-311+g(d,p)-CPCM(benzene)//UB3LYP-D3/def2svp-CPCM(benzene)
E(scf) = –7431.8192 a.u.

^{1}Int2
E(scf) = –7428.53566955 a.u.
ν_{min} = 11.7745 cm^{-1}

Ni	2.194419	8.353197	10.066210	P	0.546724	9.584393	9.128490
Ni	2.876162	12.568577	13.782309	P	2.405291	14.009875	12.146180
P	4.454020	11.330297	14.767158	P	3.208389	10.050016	11.237767
P	2.560848	6.797353	11.626701	P	1.954433	10.842669	12.620737

N	1.223017	6.899486	9.075426
C	4.413478	13.802227	15.904101
C	0.410053	8.594375	7.588835
C	4.857370	12.468628	16.143176
N	3.848628	14.085723	14.667704
C	0.798777	6.385310	6.674352
H	1.150740	5.358486	6.781817
C	4.737509	17.609464	13.785473
H	5.403479	18.402276	14.141721
C	0.964145	5.699209	9.684678
C	3.138821	15.524914	12.855001
C	-0.503298	8.638595	3.864571
H	0.192882	8.326868	3.068491
H	-0.594234	9.734752	3.823122
H	-1.490692	8.215047	3.609351
C	4.734931	16.394449	14.455753
H	5.414618	16.261769	15.295508
C	5.673014	13.061630	18.376616
C	0.800990	7.243777	7.796445
C	0.388028	6.845863	5.424236
H	0.411908	6.156697	4.574119
C	-0.037335	8.169808	5.221667
C	4.522524	14.712017	16.984264
H	4.097188	15.710907	16.890410
C	-0.013088	9.030947	6.329462
H	-0.327367	10.066730	6.192071
C	3.896825	15.314268	14.050778
C	5.134810	14.344683	18.179652
H	5.190704	15.078898	18.989708
C	5.506051	12.134328	17.339165
H	5.892938	11.125279	17.478691
C	0.057371	4.715102	9.194009
H	-0.489102	4.899467	8.269797
C	0.462342	3.241033	11.109032
C	-0.176124	3.535943	9.887999
H	-0.889521	2.816673	9.472270
C	6.390211	12.701901	19.654861
H	7.391244	13.166327	19.700213
H	6.527874	11.613904	19.750303
H	5.836028	13.051892	20.541428
C	3.157767	16.772395	12.205484
H	2.566407	16.913885	11.301876
C	1.323306	4.216179	11.618626
H	1.813950	4.026260	12.574361
C	3.904871	19.191347	11.975826
H	3.422497	19.135172	10.987618
H	4.923534	19.588706	11.832188
H	3.351415	19.940712	12.569545
C	3.925693	17.846793	12.660174
C	0.216372	1.935536	11.825006
H	-0.861927	1.732104	11.938811
H	0.665746	1.934816	12.830060
H	0.644297	1.081147	11.271212
C	1.570578	5.426021	10.947732
C	-1.207745	9.566763	9.794230
H	-1.816431	9.977819	8.969583
C	0.886161	11.365548	8.674820
H	1.118331	11.789034	9.666301
C	1.949024	7.144674	13.359771
H	2.469825	8.085826	13.592948
C	4.349198	6.220377	11.728050
H	4.658728	6.316522	10.673583
C	6.014787	11.118831	13.743549
H	5.600080	10.841338	12.757601
C	3.951044	9.654291	15.447251
H	3.971452	9.048315	14.526197
C	0.602132	14.261411	11.641095
H	0.507548	13.582127	10.775515
C	3.304711	13.714587	10.526364
H	2.905946	12.735479	10.218601
C	3.650426	8.168319	8.839013
C	1.526068	12.786462	15.164215
O	4.425603	7.699901	8.140896
O	1.070940	13.221486	16.119266
C	4.584896	4.762163	12.139660
H	4.050268	4.060866	11.484204
H	5.661804	4.534310	12.067074
H	4.277194	4.563217	13.176873
C	5.197926	7.197356	12.551803
H	5.049433	8.240675	12.234168
H	4.967173	7.130986	13.626386
H	6.267843	6.960994	12.433427
C	2.262877	6.132198	14.465468
H	1.894038	6.522785	15.428579
H	1.762286	5.167728	14.297422
H	3.339815	5.947521	14.579932
C	0.453249	7.464707	13.284940
H	0.100892	7.859323	14.251478
H	0.261536	8.226049	12.517037
H	-0.136182	6.566793	13.040321

C	2.505178	9.704668	15.961830	C	2.159193	11.451276	7.823530
H	1.793683	9.991503	15.175907	H	2.433059	12.504608	7.659800
H	2.210763	8.712724	16.340282	H	3.006139	10.953039	8.317804
H	2.406864	10.421538	16.792686	H	2.016939	10.980783	6.838545
C	4.836233	8.965443	16.499012	C	-0.262730	12.188486	8.078292
H	4.666595	9.396916	17.496146	H	0.055545	13.240833	7.988297
H	4.553649	7.900890	16.556152	H	-0.544113	11.849784	7.070711
H	5.909512	9.010772	16.284149	H	-1.166044	12.174762	8.704235
C	6.713104	12.482107	13.626494	C	4.794823	13.525278	10.812537
H	7.228789	12.734608	14.566252	H	5.323666	13.230429	9.891727
H	7.464049	12.450170	12.820676	H	5.254405	14.451978	11.192052
H	6.006668	13.293340	13.408331	H	4.942975	12.734364	11.558028
C	7.009951	10.035575	14.168339	C	3.063229	14.715810	9.393994
H	6.565287	9.031602	14.179304	H	3.413826	15.725187	9.655545
H	7.847653	10.014436	13.450928	H	3.629718	14.398392	8.502693
H	7.436927	10.243548	15.162703	H	2.005095	14.776402	9.100872
C	-1.376054	10.451975	11.034118	C	-0.358240	13.771711	12.731776
H	-0.775340	10.082165	11.877179	H	-0.175097	12.722995	13.001233
H	-1.074741	11.495310	10.860281	H	-0.267808	14.382066	13.644336
H	-2.433938	10.459155	11.345205	H	-1.398472	13.855163	12.376050
C	-1.671429	8.129355	10.061610	C	0.209934	15.676913	11.198036
H	-2.709823	8.138463	10.431892	H	-0.842269	15.676181	10.868290
H	-1.640137	7.509780	9.154153	H	0.296248	16.388618	12.033210
H	-1.043499	7.639769	10.818910	H	0.814620	16.055474	10.363877

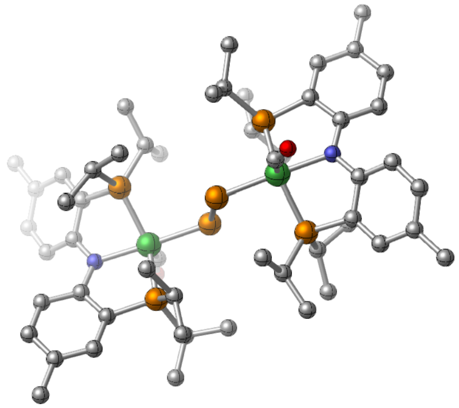

Temperature 298.150 Kelvin. Pressure 1.00000 Atm.

Zero-point correction= 1.225555 (Hartree/Particle)
Thermal correction to Energy= 1.306596
Thermal correction to Enthalpy= 1.307540
Thermal correction to Gibbs Free Energy= 1.107723
Sum of electronic and zero-point Energies= –7427.310115
Sum of electronic and thermal Energies= –7427.229073
Sum of electronic and thermal Enthalpies= –7427.228129
Sum of electronic and thermal Free Energies= –7427.427946

UB3LYP-D3/def2TZVPP-CPCM(benzene)//UB3LYP-D3/def2svp-CPCM(benzene)
E(scf) = –7432.2745 a.u.
UB3LYP-D3/6-311+g(d,p)-CPCM(benzene)//UB3LYP-D3/def2svp-CPCM(benzene)
E(scf) = –7431.8159 a.u.

3 · 2 CO
E(scf) = –7428.54037520 a.u.
ν_{min} = 6.9563 cm^{-1}

Ni	2.063603	8.282825	10.297597	C	-0.189751	8.005489	5.293261
Ni	2.816223	12.435548	13.838876	C	4.889240	14.800162	16.801439
P	3.775356	11.185229	15.363414	H	4.725752	15.844983	16.546563
P	2.832355	6.639027	11.531393	C	2.304189	6.694081	13.314924
P	1.125983	9.670551	8.870640	H	2.792757	7.618113	13.661765
P	1.991268	13.962724	12.473271	C	0.042636	8.922828	6.323810
P	3.025412	9.911824	11.485982	H	-0.187855	9.975250	6.147841
P	1.738490	10.714812	12.863935	C	3.934581	15.161651	13.969837
N	1.280742	6.825985	9.124937	C	5.466644	14.503971	18.031679
C	5.071684	10.004136	14.746206	H	5.768900	15.333850	18.678828
H	4.482923	9.290381	14.148017	C	0.787470	6.920281	13.361919
C	4.459599	13.777273	15.909214	H	0.509618	7.839109	12.823043
C	0.575898	8.532371	7.561699	H	0.450075	7.021866	14.405668
C	4.589202	12.444525	16.397210	H	0.246691	6.072962	12.910380
N	3.847231	13.973354	14.679102	C	5.175770	12.170123	17.640460
C	6.003754	10.762530	13.792320	H	5.246670	11.134036	17.982116
H	5.438451	11.224325	12.969116	C	0.298086	4.529303	9.205153
H	6.740986	10.070536	13.353743	H	-0.441339	4.753931	8.437610
H	6.553442	11.556303	14.324076	C	1.250732	2.880692	10.749098
C	0.684126	6.254869	6.768723	C	0.200113	14.464544	12.605737
H	0.988751	5.216109	6.887987	H	0.104463	15.395618	12.019916
C	4.903580	17.349318	13.411953	C	2.682913	10.277293	16.579137
H	5.706862	18.076706	13.568549	H	3.305057	10.108605	17.474023
C	1.189605	5.547157	9.645504	C	0.337476	3.247632	9.741537
C	2.974321	15.416392	12.945858	H	-0.364455	2.498580	9.360938
C	-0.794956	8.420539	3.974860	C	6.318816	12.884999	19.798545
H	-0.250966	7.979813	3.122930	H	7.414084	13.015841	19.738009
H	-0.785879	9.514579	3.851514	H	6.130440	11.849564	20.122079
H	-1.845051	8.088646	3.888687	H	5.960881	13.558723	20.594516
C	4.945842	16.151527	14.118124	C	-0.118705	14.775418	14.072266
H	5.789394	15.967413	14.781285	H	0.036644	13.886052	14.705634
C	5.652315	13.183619	18.478486	H	-1.170375	15.088838	14.176362
C	0.837872	7.160356	7.854523	H	0.518577	15.582040	14.459912
C	0.185622	6.671563	5.539316	C	2.929930	16.644658	12.272191
H	0.089130	5.933514	4.736251	H	2.145083	16.818950	11.531420

C	2.345412	13.656807	10.666132	H	0.344254	1.006194	11.363579
H	1.865183	12.676863	10.512648	H	1.854615	1.404481	12.220816
C	1.769983	14.649534	9.652525	H	1.905882	0.825158	10.542004
H	0.690766	14.818953	9.783631	C	2.180718	8.927288	16.065424
H	1.918651	14.255382	8.633060	H	1.582969	9.050111	15.150054
H	2.280509	15.622647	9.702367	H	1.535916	8.456194	16.825828
C	5.848320	9.246149	15.825778	H	3.000805	8.227421	15.849263
H	6.499602	9.924532	16.398276	C	1.520002	11.206791	16.953257
H	6.495856	8.488787	15.352740	H	1.872247	12.182658	17.318348
H	5.190894	8.722100	16.536408	H	0.901220	10.746543	17.741038
C	2.057377	3.902223	11.264225	H	0.878358	11.389472	16.075554
H	2.743223	3.667147	12.081902	C	2.741569	5.532366	14.211001
C	3.801231	18.990500	11.810578	H	2.177447	4.615794	13.983901
H	3.133643	18.958075	10.935528	H	2.539871	5.786092	15.265262
H	4.795279	19.320152	11.465930	H	3.815207	5.305161	14.124964
H	3.419800	19.777873	12.485132	C	5.119609	6.352863	10.018276
C	-0.744411	13.407287	12.025714	H	5.032012	7.387696	9.648922
H	-0.565921	13.229754	10.956277	H	4.517614	5.710554	9.358867
H	-1.790266	13.738499	12.135268	H	6.174570	6.045699	9.930134
H	-0.636117	12.444408	12.549667	C	2.006724	5.210243	10.765548
C	-1.093020	11.556524	8.738067	C	-1.300583	9.440174	10.126727
H	-1.452392	11.114584	7.795373	H	-2.096324	9.894303	10.738875
H	-0.467762	12.428233	8.498470	H	-0.763730	8.705965	10.747292
H	-1.975996	11.929204	9.283499	H	-1.774506	8.893144	9.295750
C	4.659120	6.266535	11.480676	C	2.322400	10.881485	8.093444
H	4.763768	5.223875	11.828935	H	2.520468	11.574868	8.925230
C	3.858586	13.471528	10.494530	C	1.872793	11.687762	6.869557
H	4.397185	14.408316	10.706958	H	1.790128	11.045853	5.980192
H	4.092857	13.172244	9.459752	H	2.631366	12.457053	6.647313
H	4.240499	12.687488	11.165968	H	0.913708	12.201137	7.012119
C	5.480768	7.190004	12.386008	C	3.624282	10.124166	7.784683
H	6.552301	6.946766	12.295051	H	4.033952	9.638890	8.682387
H	5.209170	7.092602	13.447641	H	4.382968	10.822334	7.394573
H	5.350234	8.244922	12.097038	H	3.454782	9.346746	7.021686
C	-0.354908	10.532126	9.602251	C	3.328170	2.769438	8.282849
H	0.082188	11.040666	10.477859	O	3.395909	3.857477	7.979258
C	3.871125	17.653105	12.505203	C	1.682020	15.001277	17.088540
C	1.342034	1.460411	11.247928	O	1.777782	16.044610	16.661714

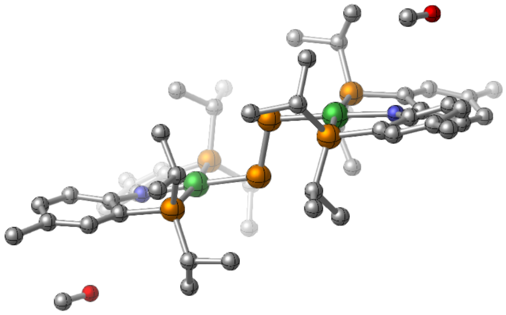

Temperature 298.150 Kelvin. Pressure 1.00000 Atm.

Zero-point correction= 1.221937 (Hartree/Particle)
Thermal correction to Energy= 1.305390
Thermal correction to Enthalpy= 1.306334
Thermal correction to Gibbs Free Energy= 1.096991
Sum of electronic and zero-point Energies= –7427.318439
Sum of electronic and thermal Energies= –7427.234986
Sum of electronic and thermal Enthalpies= –7427.234041
Sum of electronic and thermal Free Energies= –7427.443384

UB3LYP-D3/def2TZVPP-CPCM(benzene)//UB3LYP-D3/def2svp-CPCM(benzene)
E(scf) = –7432.2934 a.u.
UB3LYP-D3/6-311+g(d,p)-CPCM(benzene)//UB3LYP-D3/def2svp-CPCM(benzene)
E(scf) = –7431.8327 a.u.

^{1}TS$_{1,2}$
E(scf) = –3714.19488539 a.u.
ν_{min} = –196.0374 cm^{-1}

Ni	2.774259	2.741038	7.066343	H	5.640753	0.920261	8.553627
P	2.424079	2.491028	4.908608	H	6.945215	2.125160	8.722894
P	3.238441	2.581950	9.219587	H	5.640563	2.371959	7.528694
P	2.497486	4.839812	6.971514	C	2.634458	0.085334	8.304033
N	2.875818	0.730075	7.101779	C	2.767647	4.982911	10.654759
C	3.733918	-1.231756	5.821920	H	3.834088	5.065500	10.912823
H	3.987830	-1.781821	6.727720	H	2.191282	5.481824	11.451164
C	5.058341	2.746020	9.609433	H	2.601724	5.539151	9.719384
H	5.255116	3.825945	9.505163	C	2.157970	-1.243866	8.450893
C	3.414138	3.445993	3.649475	H	1.940557	-1.841180	7.565693
H	3.339339	2.860423	2.717890	C	1.919108	-1.790250	9.708294
C	2.556781	0.260531	10.759188	H	1.540349	-2.815628	9.770229
H	2.691385	0.862561	11.661927	C	3.167734	0.067132	5.922833
C	4.012697	-1.804690	4.585365	C	0.357369	2.119145	2.978108
H	4.459399	-2.803958	4.560217	H	0.944026	2.676665	2.231819
O	5.651946	5.647457	6.921954	H	-0.708701	2.259087	2.734175
C	5.865936	1.998868	8.539366	H	0.587184	1.048164	2.864253

C	2.128929	-1.065653	10.896009	C	-0.213730	1.820775	5.438796
C	5.425562	2.301462	11.028105	H	0.055992	0.752283	5.453085
H	4.879748	2.865160	11.800473	H	-1.283619	1.898858	5.186330
H	6.502648	2.461083	11.201810	H	-0.067181	2.217797	6.454965
H	5.218850	1.229624	11.174528	C	2.304254	3.528042	10.525316
C	4.041574	-1.796538	2.043887	H	2.518568	3.008156	11.474209
H	3.988056	-1.074782	1.214444	C	0.628625	2.588273	4.410458
H	3.316054	-2.600950	1.828846	H	0.393418	3.663371	4.499282
H	5.043075	-2.257777	2.025787	C	4.882897	3.473110	4.094877
C	3.758965	-1.138159	3.371935	H	5.003085	4.068807	5.012582
C	2.868639	4.852674	3.382175	H	5.506895	3.928461	3.308715
H	1.839976	4.839522	2.991068	H	5.267953	2.460243	4.290459
H	3.502291	5.356275	2.633564	C	3.245819	0.160837	3.461748
H	2.881227	5.463349	4.299389	H	3.077186	0.721021	2.538037
C	2.964892	0.761474	4.696428	C	4.781685	5.628601	7.670002
C	1.892736	-1.693711	12.247650	C	0.799372	3.428468	10.240053
H	1.891609	-0.939953	13.049919	H	0.552323	3.929184	9.290435
H	2.675123	-2.434045	12.491651	H	0.229842	3.918877	11.046314
H	0.927881	-2.226395	12.283486	H	0.464374	2.382046	10.173050
C	2.794817	0.834173	9.503714				

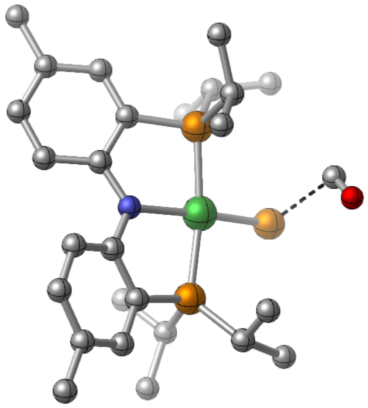

Temperature 298.150 Kelvin. Pressure 1.00000 Atm.

Zero-point correction= 0.608889 (Hartree/Particle)
Thermal correction to Energy= 0.649111
Thermal correction to Enthalpy= 0.650056
Thermal correction to Gibbs Free Energy= 0.535578
Sum of electronic and zero-point Energies= –3713.585996
Sum of electronic and thermal Energies= –3713.545774
Sum of electronic and thermal Enthalpies= –3713.544830
Sum of electronic and thermal Free Energies= –3713.659308

UB3LYP-D3/def2TZVPP-CPCM(benzene)//UB3LYP-D3/def2svp-CPCM(benzene)
E(scf) = –3716.0728 a.u.

UB3LYP-D3/6-311+g(d,p)-CPCM(benzene)//UB3LYP-D3/def2svp-CPCM(benzene)
E(scf) = –3715.8434 a.u.

^{1}TS$_{1,6}$
E(scf) = –7428.51630357 a.u.
ν_{min} = –230.8412 cm^{-1}

Ni	5.511809	3.871026	9.288367	P	5.760297	4.764792	4.053381
P	3.325197	4.097170	8.987441	N	3.742991	2.820261	4.173048
P	7.620300	3.882788	9.963114	C	1.185128	0.795874	2.329141
P	5.791696	2.927828	7.295353	H	0.238106	0.947115	1.800827
O	9.225524	0.032876	5.698939	C	3.481311	6.499244	4.542610
N	5.138814	4.727005	11.060996	H	4.134532	7.369309	4.481467
C	2.245810	4.497370	13.432171	C	3.066065	1.780281	3.560535
H	2.053767	4.537191	14.509387	C	2.960915	-0.626524	3.049781
C	8.651538	0.869607	5.146761	H	3.417590	-1.615419	3.105828
C	8.589905	5.784572	11.890627	C	1.706000	-0.506783	2.446279
H	9.605993	5.518579	11.594035	C	1.834540	1.904312	2.858058
C	3.867717	4.598369	11.599401	H	1.389500	2.887645	2.715073
C	1.476830	4.178548	11.190038	C	3.172499	4.066934	4.406095
H	0.667850	3.985098	10.483584	C	3.656170	0.481563	3.560196
C	1.169963	4.276424	12.552488	C	4.019540	5.216890	4.390928
C	3.553122	4.654595	12.982537	C	0.950520	-1.704035	1.924515
H	4.351357	4.794104	13.711697	H	0.086564	-1.949592	2.567437
C	6.166177	5.420900	11.680491	H	0.552345	-1.522160	0.912308
C	2.787884	4.313368	10.714799	H	1.590796	-2.598455	1.879334
C	7.505658	5.119343	11.302894	C	2.114671	6.711794	4.781975
C	-0.244051	4.146966	13.063769	C	1.298596	5.575005	4.872972
H	-0.632350	5.113696	13.430336	H	0.236765	5.693872	5.108643
H	-0.306621	3.438701	13.906905	C	1.805469	4.288057	4.696097
H	-0.928257	3.794259	12.276824	H	1.136213	3.436246	4.806236
C	8.413119	6.795047	12.843073	C	1.573223	8.107990	4.966737
C	7.087268	7.132171	13.172511	H	2.092768	8.635574	5.784604
H	6.904487	7.946108	13.881756	H	1.706573	8.718884	4.057442
C	5.994477	6.475017	12.616473	H	0.499462	8.096360	5.206582
H	4.988997	6.798510	12.885874	C	6.496458	-0.197559	2.835047
C	9.582441	7.498461	13.487673	H	7.344027	-0.609559	3.409747
H	9.691986	7.212577	14.548817	C	5.448471	-0.793336	5.599160
H	9.459344	8.594250	13.464527	H	6.397688	-0.536212	6.096412
H	10.529700	7.255405	12.982197	C	5.960502	5.084004	2.214098
P	7.650919	2.474562	5.604219	H	6.988214	4.743461	2.000104
O	7.639619	0.876578	7.961809	C	6.990501	5.851048	4.957736
C	7.101024	1.709591	7.265727	H	7.067989	5.306691	5.913401
Ni	5.606480	2.603685	4.661232	C	2.891454	5.647353	8.041590
P	5.360776	0.450988	4.198207	H	2.842119	5.302878	6.998166

C	2.413026	2.668650	8.210514	H	9.136409	6.163930	4.934626
H	2.962164	2.559017	7.257486	H	8.637792	4.728662	4.019753
C	8.886310	4.478963	8.727978	H	8.386159	6.360454	3.337044
H	8.666321	3.838074	7.859120	C	10.366450	4.291962	9.073958
C	8.227452	2.278935	10.716460	H	10.980857	4.586657	8.206581
H	8.719244	1.763057	9.876606	H	10.682998	4.915752	9.923336
C	5.926625	-1.303471	1.937490	H	10.607699	3.243880	9.304135
H	5.093003	-0.925331	1.326895	C	8.550980	5.922463	8.332146
H	6.713887	-1.651505	1.247996	H	9.125984	6.212698	7.438149
H	5.569284	-2.177462	2.496639	H	7.480387	6.035149	8.100213
C	7.017893	0.958430	1.972145	H	8.793836	6.628613	9.140902
H	6.201059	1.407839	1.386298	C	7.005617	1.445769	11.132002
H	7.475601	1.752784	2.578223	H	6.376810	1.987511	11.857455
H	7.771812	0.585493	1.259261	H	6.390582	1.199405	10.254477
C	4.311143	-0.497594	6.583809	H	7.331883	0.501420	11.598428
H	4.303682	0.564075	6.864483	C	9.212885	2.458225	11.875890
H	3.330630	-0.752263	6.150535	H	9.559509	1.469326	12.219484
H	4.445750	-1.088406	7.504068	H	10.100977	3.042802	11.596204
C	5.504658	-2.277622	5.223553	H	8.734542	2.963388	12.729255
H	6.365753	-2.519545	4.583853	C	0.930444	2.851029	7.871512
H	5.603158	-2.878266	6.143215	H	0.581912	1.972725	7.302533
H	4.585973	-2.610310	4.716074	H	0.301859	2.926232	8.771265
C	5.814357	6.552142	1.799839	H	0.754861	3.735262	7.245427
H	5.928194	6.646556	0.706655	C	2.663815	1.415240	9.058935
H	4.816245	6.937802	2.062024	H	2.154685	1.487752	10.033033
H	6.567178	7.202395	2.267335	H	2.283460	0.522828	8.537574
C	4.983383	4.188989	1.439839	H	3.738957	1.266646	9.245011
H	5.047076	3.144235	1.769898	C	4.045561	6.651291	8.165911
H	3.943078	4.518479	1.590852	H	4.211961	6.947526	9.214018
H	5.200932	4.236231	0.360483	H	4.985436	6.221320	7.789939
C	6.624950	7.301724	5.287731	H	3.822039	7.557871	7.580173
H	7.454833	7.757832	5.853115	C	1.556055	6.285001	8.435599
H	6.465225	7.914181	4.387534	H	1.351513	7.145144	7.777862
H	5.729870	7.366526	5.917963	H	0.708240	5.592019	8.346880
C	8.359263	5.764727	4.262710	H	1.585240	6.648383	9.474765

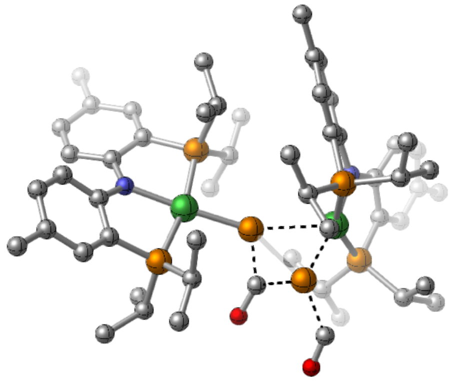

Temperature 298.150 Kelvin. Pressure 1.00000 Atm.

Zero-point correction= 1.226135 (Hartree/Particle)
Thermal correction to Energy= 1.305395
Thermal correction to Enthalpy= 1.306339
Thermal correction to Gibbs Free Energy= 1.112300
Sum of electronic and zero-point Energies= –7427.290169
Sum of electronic and thermal Energies= –7427.210909
Sum of electronic and thermal Enthalpies= –7427.209965
Sum of electronic and thermal Free Energies= –7427.404004

UB3LYP-D3/def2TZVPP-CPCM(benzene)//UB3LYP-D3/def2svp-CPCM(benzene)
E(scf) = –7432.2588 a.u.
UB3LYP-D3/6-311+g(d,p)-CPCM(benzene)//UB3LYP-D3/def2svp-CPCM(benzene)
E(scf) = –7431.7974 a.u.

^{1}TS$_{6,3}$
E(scf) = –7315.27105112 a.u.
ν_{min} = –117.2410 cm^{-1}

Ni	4.964830	4.055455	10.204303	H	5.821560	6.077465	14.288397
P	3.344777	5.582927	10.117250	C	7.026076	5.122477	11.987254
P	6.796032	2.829415	10.547062	C	6.515787	1.237709	11.497885
P	4.039530	2.795969	8.523672	H	6.385081	0.470398	10.717449
N	5.673513	5.190112	11.674261	C	3.557557	6.354022	11.750095
C	3.992796	7.192441	14.359824	C	7.758250	3.970666	11.590816
H	4.162054	7.482885	15.401748	C	9.831879	4.840052	12.560716
C	9.121664	3.838329	11.890941	C	9.121203	6.009012	12.892849
H	9.653015	2.936310	11.582303	H	9.652033	6.837179	13.373348
C	4.777999	5.981673	12.380852	C	3.582425	6.879960	8.783630
C	2.621872	7.170121	12.401688	H	2.900313	6.561643	7.980655
H	1.708205	7.460994	11.881194	C	1.603827	4.934848	9.962212
C	2.818582	7.619689	13.711596	C	7.813145	2.458511	9.025394
C	4.944902	6.400544	13.727286	H	7.055583	2.081588	8.316759

C	7.766286	6.157222	12.616921
H	7.274333	7.096840	12.868413
P	5.117086	2.976884	6.618852
O	4.947321	0.064632	8.673845
C	4.634955	1.195509	8.688162
Ni	3.803100	2.233811	4.926418
P	4.395638	0.098263	5.031976
P	2.909274	4.196879	4.570310
N	2.947854	1.700971	3.184493
C	3.994861	-0.733650	0.528551
H	3.938253	-0.844297	-0.559443
C	0.805728	4.684751	2.665772
H	0.741562	5.707754	3.040306
C	3.468760	0.601605	2.518280
C	4.752036	-1.494971	2.663153
H	5.288935	-2.223516	3.273665
C	4.657536	-1.717655	1.284051
C	3.418793	0.389485	1.114289
H	2.950055	1.134890	0.472249
C	1.935301	2.495177	2.675707
C	4.076878	5.580826	4.090947
H	4.389864	6.002109	5.062777
C	4.196149	-0.364553	3.275897
C	1.799907	3.829939	3.165713
C	-0.121916	4.268909	1.704654
C	-0.036082	2.929584	1.279623
H	-0.774900	2.547492	0.567356
C	3.229025	-0.980086	6.037485
C	6.132272	-0.309848	5.573412
C	1.837241	4.799929	5.977003
H	2.565194	4.774200	6.805965
C	0.950301	2.066841	1.743248
H	0.948151	1.031600	1.402337
H	1.670029	4.365346	9.018677
H	3.747433	-1.112699	6.999434
H	6.185853	0.264501	6.513767
C	-1.172923	5.200225	1.151925
H	-2.171267	4.731349	1.152215
H	-1.237799	6.130348	1.737524
H	-0.954891	5.483766	0.106819
C	5.239082	-2.948265	0.632050
H	5.801973	-2.695478	-0.281970
H	5.921987	-3.481676	1.311291
H	4.449684	-3.661024	0.333286
C	1.821237	8.511547	14.409269

H	0.889748	8.607722	13.830744
H	1.559606	8.122497	15.407544
H	2.224363	9.528544	14.559131
C	11.293671	4.690312	12.903422
H	11.739756	3.817910	12.401945
H	11.871641	5.581868	12.608362
H	11.441907	4.558912	13.989801
C	5.305299	4.958610	3.408140
H	5.013229	4.410784	2.496736
H	6.019505	5.745956	3.115141
H	5.822855	4.252625	4.073594
C	3.472461	6.684985	3.215927
H	4.230090	7.464351	3.029028
H	3.159239	6.283332	2.239886
H	2.601307	7.173077	3.673446
C	1.251444	6.210747	5.879450
H	0.688570	6.440929	6.798620
H	2.028366	6.980913	5.768967
H	0.545581	6.306393	5.041631
C	0.756344	3.748804	6.257288
H	0.219178	3.991295	7.188970
H	0.018573	3.711161	5.440470
H	1.194892	2.745728	6.364832
C	3.208529	8.299460	9.220986
H	3.320881	8.990362	8.369037
H	2.171351	8.375163	9.579119
H	3.867240	8.649025	10.030872
C	5.023000	6.798476	8.255744
H	5.751561	6.979985	9.062666
H	5.232942	5.807531	7.820652
H	5.183632	7.557351	7.471848
C	0.473433	5.959076	9.820277
H	0.673028	6.702398	9.035491
H	-0.459446	5.437353	9.549125
H	0.283400	6.494979	10.761511
C	1.343580	3.931913	11.093028
H	1.307647	4.436729	12.071426
H	0.379202	3.423119	10.933650
H	2.132192	3.163970	11.134145
C	8.328194	3.780251	8.441135
H	9.138308	4.206562	9.053019
H	8.710350	3.607399	7.422533
H	7.520912	4.525683	8.367798
C	8.912373	1.400772	9.153400
H	9.355972	1.223780	8.159175

H	9.727772	1.717429	9.821680
H	8.522625	0.436416	9.512007
C	7.677384	0.829861	12.411235
H	7.442506	-0.131289	12.897843
H	8.623050	0.703424	11.865846
H	7.840490	1.577584	13.202443
C	5.202508	1.365202	12.285543
H	5.250783	2.198805	13.005327
H	4.348107	1.554356	11.617539
H	5.001503	0.439241	12.848649
C	6.461037	-1.772442	5.885059
H	6.447797	-2.408243	4.986603
H	7.476956	-1.835070	6.311003
H	5.771765	-2.205262	6.624756
C	7.127081	0.327742	4.596619
H	8.147961	0.275653	5.010288
H	7.125165	-0.186226	3.622658
H	6.884974	1.388624	4.425087
C	2.954996	-2.354553	5.419874
H	3.876539	-2.929678	5.247721
H	2.311658	-2.945202	6.093834
H	2.436711	-2.259139	4.453167
C	1.931218	-0.198475	6.290463
H	2.129600	0.720047	6.863638
H	1.455132	0.098630	5.341995
H	1.213721	-0.812825	6.859881

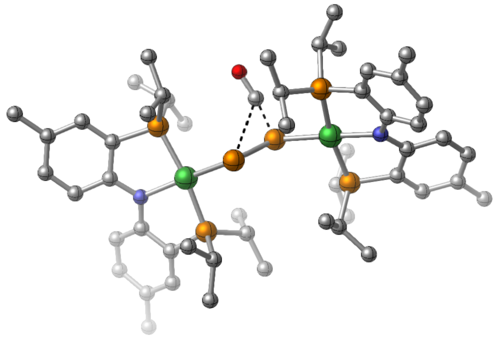

Temperature 298.150 Kelvin. Pressure 1.00000 Atm.

Zero-point correction= 1.216361 (Hartree/Particle)
Thermal correction to Energy= 1.294185
Thermal correction to Enthalpy= 1.295129
Thermal correction to Gibbs Free Energy= 1.100297
Sum of electronic and zero-point Energies= –7314.054690
Sum of electronic and thermal Energies= –7313.976866
Sum of electronic and thermal Enthalpies= –7313.975922
Sum of electronic and thermal Free Energies= –7314.170754

UB3LYP-D3/def2TZVPP-CPCM(benzene)//UB3LYP-D3/def2svp-CPCM(benzene)
E(scf) = –7318.8862 a.u.
UB3LYP-D3/6-311+g(d,p)-CPCM(benzene)//UB3LYP-D3/def2svp-CPCM(benzene)
E(scf) = –7318.435654 a.u.

^{3}1

E(scf) = –3714.25009283 a.u.
ν_{min} = 21.9933 cm^{-1}

Ni	3.763110	2.908952	7.007221
P	2.740827	2.623181	4.880271
P	3.739125	2.677966	9.349331
P	5.604175	4.325094	6.378989

N	3.593822	0.920119	7.013862
C	4.415749	-0.988115	5.651729
H	4.781600	-1.504451	6.540755
C	5.389308	2.670376	10.231351
H	5.731401	3.714715	10.158943
C	3.029486	3.582844	3.305494
H	2.491926	3.044135	2.506971
C	2.643690	0.342683	10.556156
H	2.637142	0.903309	11.494749
C	4.591320	-1.565735	4.396999
H	5.086249	-2.539465	4.327503
O	5.719516	5.769155	8.808023
C	6.363981	1.781546	9.445719
H	6.041582	0.727585	9.466837
H	7.368543	1.837151	9.895837
H	6.440601	2.098120	8.395119
C	3.152894	0.260938	8.148207
C	3.049904	5.089261	10.611333
H	4.014788	5.121991	11.138320
H	2.318788	5.654764	11.212188
H	3.183300	5.615670	9.653386
C	2.596178	-1.046438	8.151802
H	2.522745	-1.603251	7.217966
C	2.108785	-1.627564	9.317661
H	1.683943	-2.635128	9.262426
C	3.789846	0.271152	5.794353
C	0.244452	1.820345	3.730191
H	0.406313	2.444553	2.838446
H	-0.845204	1.719239	3.866237
H	0.650466	0.816476	3.525775
C	2.128477	-0.958546	10.554420
C	5.318661	2.269588	11.707974
H	4.638044	2.910420	12.289524
H	6.320007	2.356188	12.161821
H	4.993449	1.224097	11.823972
C	4.337858	-1.587005	1.872303
H	5.375579	-1.928958	1.723809
H	4.087301	-0.901075	1.049045
H	3.691326	-2.476295	1.773589
C	4.162758	-0.928179	3.218600
C	2.428598	4.989792	3.442717
H	1.339567	4.971176	3.603129
H	2.616404	5.568519	2.523758
H	2.892511	5.532959	4.282441
C	3.395287	0.939425	4.602668
C	1.616645	-1.617934	11.811418
H	1.498683	-0.891765	12.630349
H	2.307143	-2.403959	12.165247
H	0.640113	-2.102645	11.646050
C	3.141896	0.954439	9.396821
C	0.502922	1.612613	6.243313
H	0.877266	0.579146	6.180391
H	-0.593419	1.570147	6.348733
H	0.918447	2.054921	7.159501
C	2.546682	3.656536	10.397159
H	2.475174	3.152626	11.375718
C	0.873938	2.419199	4.991911
H	0.505856	3.451359	5.126635
C	4.517207	3.647631	2.933241
H	5.085481	4.237596	3.666976
H	4.627456	4.136954	1.951489
H	4.977883	2.651432	2.867682
C	3.574057	0.336310	3.350484
H	3.253161	0.862013	2.447194
C	5.660044	5.149932	7.806703
C	1.165966	3.633466	9.728396
H	1.204818	4.109662	8.734038
H	0.436949	4.190281	10.339502
H	0.789527	2.607299	9.600324

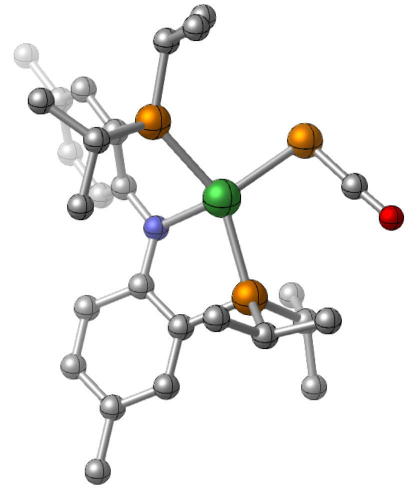

Temperature   298.150 Kelvin. Pressure   1.00000 Atm.

Zero-point correction=   0.610873 (Hartree/Particle)
Thermal correction to Energy=   0.651117
Thermal correction to Enthalpy=   0.652061
Thermal correction to Gibbs Free Energy=   0.536092
Sum of electronic and zero-point Energies=   –3713.639219
Sum of electronic and thermal Energies=   –3713.598976
Sum of electronic and thermal Enthalpies=   –3713.598032
Sum of electronic and thermal Free Energies=   –3713.714001

UB3LYP-D3/def2TZVPP-CPCM(benzene)//UB3LYP-D3/def2svp-CPCM(benzene)
E(scf) = -3716.1221 a.u.
UB3LYP-D3/6-311+g(d,p)-CPCM(benzene)//UB3LYP-D3/def2svp-CPCM(benzene)
E(scf) = -3715.8921 a.u.

^{3}2
E(scf) = –3600.97145347 a.u.
ν_{min} = 13.2291 cm^{-1}

Ni	8.871716	6.775305	17.762850
P	8.960247	5.114706	16.317706
P	8.788651	8.120785	19.497392
P	9.617576	8.281784	16.441623
N	8.033577	5.414402	18.981494
C	7.742139	3.979586	17.067624
C	8.461419	5.249026	14.527201
H	8.288598	4.214354	14.186307
C	5.737188	2.522361	18.314719
H	4.922927	1.981009	18.807283
C	7.129885	2.905700	16.409946
H	7.427106	2.659995	15.386661
C	6.126200	2.141109	17.017994
C	8.125203	5.609217	20.350241
C	8.436447	6.916775	20.824680
C	7.389243	4.330370	18.402649
C	10.622323	2.906927	15.614377
H	9.922309	2.192074	16.075390
H	11.629876	2.460608	15.654067
H	10.350357	3.015628	14.553324
C	10.613043	4.242055	16.364438
H	11.296514	4.952499	15.867925
C	9.553648	5.869898	13.647497
H	9.801285	6.890194	13.979889

H	9.197100	5.929407	12.605828	C	11.056940	4.082437	17.825465
H	10.480001	5.275572	13.646001	H	11.098991	5.051774	18.344919
C	8.019499	4.594252	21.338477	H	12.056580	3.620218	17.868619
H	7.867882	3.558776	21.035147	H	10.358608	3.437571	18.382828
C	7.387208	9.349547	19.450867	C	10.314929	9.020355	20.084154
H	7.712555	10.077512	18.687423	H	10.134379	9.247067	21.148310
C	8.147630	4.885228	22.693072	C	8.552874	7.186729	22.194750
H	8.067560	4.065270	23.414219	H	8.792082	8.201398	22.524939
C	11.508389	8.060138	19.983457	C	6.339131	3.579270	18.991960
H	11.322817	7.121662	20.528976	H	5.967983	3.851769	19.979624
H	12.411916	8.528563	20.406566	C	8.394282	6.188240	23.163026
H	11.710809	7.807108	18.930214	C	8.492330	6.483413	24.639593
C	6.126428	8.641078	18.936689	H	7.500372	6.454719	25.124555
H	6.294305	8.198297	17.942829	H	8.919881	7.480663	24.825693
H	5.291828	9.356979	18.861240	H	9.122662	5.743097	25.159674
H	5.819924	7.831815	19.619406	C	10.572918	10.337612	19.344008
C	5.482689	0.968077	16.320039	H	10.711243	10.168488	18.264655
H	5.824396	0.007550	16.745021	H	11.491475	10.805156	19.735941
H	5.721717	0.955152	15.245501	H	9.752983	11.060129	19.474040
H	4.384988	0.988962	16.421731	C	7.142552	10.071360	20.779014
C	7.138024	6.021415	14.437415	H	6.804830	9.366369	21.554653
H	6.351026	5.552064	15.047708	H	6.353237	10.830905	20.652937
H	6.787249	6.052474	13.392922	H	8.041273	10.585984	21.152705
H	7.269548	7.057514	14.787814				

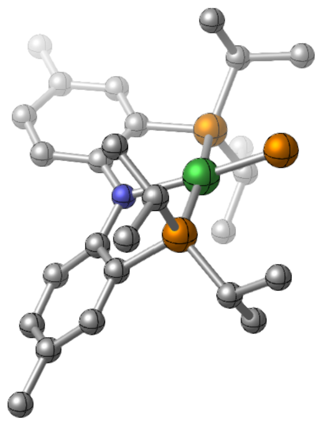

Temperature 298.150 Kelvin. Pressure 1.00000 Atm.

Zero-point correction= 0.602955 (Hartree/Particle)
Thermal correction to Energy= 0.640359
Thermal correction to Enthalpy= 0.641303
Thermal correction to Gibbs Free Energy= 0.532597
Sum of electronic and zero-point Energies= –3600.368498
Sum of electronic and thermal Energies= –3600.331094

Sum of electronic and thermal Enthalpies= –3600.330150
Sum of electronic and thermal Free Energies= –3600.438857

UB3LYP-D3/def2TZVPP-CPCM(benzene)//UB3LYP-D3/def2svp-CPCM(benzene)
E(scf) = –3602.7143 a.u.
UB3LYP-D3/6-311+g(d,p)-CPCM(benzene)//UB3LYP-D3/def2svp-CPCM(benzene)
E(scf) = –3602.4989 a.u.

DLPNO-CCSD(T)/def2svp-CPCM(benzene)
E(scf) = –3595.018831904572 a.u.

^{3}3

E(scf) = -7202.04294299 a.u.
ν_{min} = 8.0573 cm^{-1}

Ni	1.477908	8.317122	10.574317
Ni	2.651278	12.653715	13.733701
P	2.833518	11.350699	15.535653
P	3.253326	7.050487	11.057306
P	-0.114824	9.428132	9.514949
P	2.796405	14.141907	12.100209
P	1.437296	9.535813	12.410352
P	0.963063	11.497280	12.762803
N	1.104594	6.856707	9.237253
C	3.837813	9.804365	15.263301
H	3.154776	9.166069	14.677579
C	4.305023	13.606493	15.975915
C	-0.286207	8.407056	8.015899
C	3.803941	12.443313	16.627263
N	4.114648	13.729003	14.607697
C	5.046353	10.151593	14.383937
H	4.729311	10.571524	13.417599
H	5.648829	9.249232	14.188166
H	5.697829	10.892351	14.875122
C	0.493584	6.430714	6.860897
H	1.098692	5.525418	6.814341
C	6.798936	16.005864	13.317984
H	7.805196	16.337819	13.594100
C	1.554670	5.579541	9.536871
C	4.302666	15.038485	12.586061
C	-1.711822	8.431190	4.448426
H	-1.081774	8.358811	3.546085
H	-2.078860	9.466661	4.520488
H	-2.588969	7.782061	4.277168
C	6.160590	15.072523	14.126658
H	6.683001	14.688215	15.002509
C	4.598986	13.201871	18.818051
C	0.444333	7.181559	8.067806
C	-0.185656	6.844654	5.720654
H	-0.107390	6.235066	4.814344
C	-0.954327	8.023778	5.688416
C	4.887248	14.591402	16.820071
H	5.205148	15.544582	16.399094
C	3.820890	6.860852	12.823748
H	3.915476	7.917653	13.122009
C	-0.972898	8.794629	6.855798
H	-1.531530	9.732864	6.853888
C	4.852892	14.600543	13.825581
C	5.028111	14.384702	18.188189
H	5.476359	15.179072	18.794019
C	2.700894	6.240213	13.669590
H	1.742431	6.756988	13.509691
H	2.952867	6.317616	14.740228
H	2.561097	5.175109	13.427534
C	3.967309	12.253218	18.006458
H	3.576465	11.344981	18.473336
C	0.954416	4.373421	9.092098
H	0.058296	4.415450	8.472487
C	2.594706	3.002563	10.290356
C	1.451300	15.426282	11.957856
H	1.895358	16.265181	11.393417
C	1.358817	10.881011	16.585359
H	1.771633	10.661668	17.583917
C	1.464735	3.132569	9.462467
H	0.959806	2.229507	9.104140
C	4.802511	12.982208	20.296965
H	5.859498	12.764293	20.532595

H	4.202913	12.136208	20.666963	H	5.688024	8.771848	11.603214
H	4.523786	13.874913	20.881275	H	4.605795	9.619580	10.481800
C	1.105319	15.909049	13.373645	C	-1.781483	9.517842	10.358702
H	0.653463	15.092923	13.960996	H	-1.675729	10.376688	11.045572
H	0.383993	16.741299	13.329972	C	6.195074	16.536205	12.161637
H	1.998464	16.256075	13.916006	C	3.148163	1.645176	10.649598
C	4.946066	16.013883	11.809885	H	2.354423	0.963882	10.999279
H	4.463611	16.378519	10.900453	H	3.906141	1.714458	11.444908
C	3.077825	13.447051	10.394203	H	3.624970	1.159004	9.780150
H	2.112977	12.958096	10.191076	C	0.597269	9.641894	16.102798
C	3.364087	14.449330	9.272376	H	0.150511	9.802678	15.112215
H	2.645533	15.283180	9.251001	H	-0.215767	9.416800	16.813278
H	3.306668	13.938400	8.296924	H	1.238400	8.749685	16.039052
H	4.376895	14.868564	9.357728	C	0.442141	12.107792	16.694664
C	4.247028	9.071746	16.543060	H	0.984722	12.990675	17.067456
H	4.940562	9.679431	17.145388	H	-0.390861	11.902020	17.386820
H	4.763959	8.131795	16.285182	H	0.017017	12.358826	15.709680
H	3.383925	8.811078	17.174671	C	5.172895	6.180249	13.065420
C	3.172138	4.187221	10.764674	H	5.148968	5.107799	12.821322
H	4.036533	4.119120	11.428148	H	5.436185	6.264054	14.133410
C	6.876879	17.603739	11.341555	H	5.989613	6.640849	12.490817
H	7.931972	17.350376	11.144044	C	4.391299	7.569970	8.571670
H	6.875518	18.577836	11.862244	H	3.619350	8.337404	8.404535
H	6.378210	17.749096	10.370871	H	3.991386	6.615043	8.199811
C	0.214700	14.912088	11.211708	H	5.277379	7.831388	7.970360
H	0.433834	14.646642	10.166611	C	2.665527	5.446945	10.421568
H	-0.564094	15.692438	11.198139	C	-1.971864	8.240014	11.190850
H	-0.207235	14.021480	11.704160	H	-2.942922	8.267634	11.712103
C	-2.978406	9.737428	9.425677	H	-1.176384	8.125853	11.941287
H	-3.107590	8.879287	8.748122	H	-1.959520	7.345649	10.545776
H	-2.890310	10.642674	8.810587	C	0.413317	11.142927	9.008361
H	-3.899428	9.830422	10.024985	H	0.740337	11.540148	9.985417
C	4.766830	7.493999	10.057213	C	-0.632251	12.105206	8.440997
H	5.496071	6.678131	10.202362	H	-1.047461	11.757726	7.482741
C	4.147104	12.353734	10.483923	H	-0.159174	13.084280	8.253887
H	5.121686	12.771452	10.784811	H	-1.461624	12.274180	9.142738
H	4.274108	11.860899	9.507352	C	1.643366	11.016494	8.101039
H	3.857188	11.588909	11.219673	H	2.413119	10.379001	8.563524
C	5.355376	8.818489	10.555683	H	2.086585	12.007810	7.918933
H	6.224155	9.107356	9.941722	H	1.379725	10.577463	7.126360

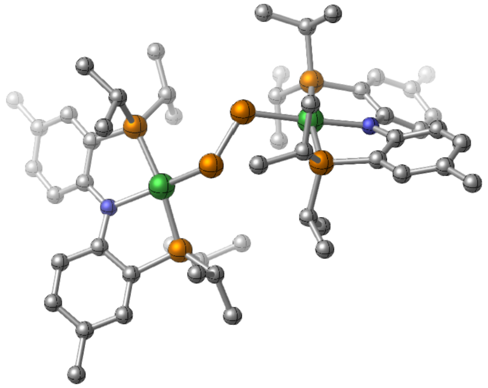

Temperature 298.150 Kelvin. Pressure 1.00000 Atm.

Zero-point correction= 1.208043 (Hartree/Particle)
Thermal correction to Energy= 1.284506
Thermal correction to Enthalpy= 1.285451
Thermal correction to Gibbs Free Energy= 1.092331
Sum of electronic and zero-point Energies= –7200.834900
Sum of electronic and thermal Energies= –7200.758437
Sum of electronic and thermal Enthalpies= –7200.757492
Sum of electronic and thermal Free Energies= –7200.950612

UB3LYP-D3/def2TZVPP-CPCM(benzene)//UB3LYP-D3/def2svp-CPCM(benzene)
E(scf) = –7205.5241 a.u.
UB3LYP-D3/6-311+g(d,p)-CPCM(benzene)//UB3LYP-D3/def2svp-CPCM(benzene)
E(scf) = –7205.0892 a.u.

^{3}TS$_{1,2}$
E(scf) = –3714.19890432 a.u.
ν_{min} = –219.7548 cm^{-1}

Ni	2.869408	2.646698	6.908281	O	4.989271	5.804800	7.978533
P	2.625064	2.419305	4.736700	C	6.000204	1.932585	8.229168
P	3.401077	2.487641	9.050868	H	5.797135	0.850185	8.265019
P	2.338088	4.743168	6.886930	H	7.085279	2.080957	8.352779
N	3.060245	0.647926	6.927901	H	5.713422	2.289164	7.228256
C	3.970076	-1.287585	5.643323	C	2.848906	-0.013265	8.129757
H	4.233379	-1.834582	6.548185	C	2.995257	4.868711	10.532015
C	5.239495	2.677706	9.335113	H	4.073788	4.961405	10.727080
H	5.417181	3.757820	9.210892	H	2.461525	5.357650	11.363511
C	3.715864	3.388084	3.574659	H	2.768610	5.428203	9.611476
H	3.680563	2.840112	2.617758	C	2.406040	-1.353988	8.270250
C	2.799604	0.138881	10.586985	H	2.190695	-1.948403	7.382609
H	2.931931	0.736253	11.493214	C	2.196983	-1.917350	9.525936
C	4.266533	-1.851433	4.406712	H	1.843348	-2.951901	9.583216
H	4.735257	-2.840575	4.381708	C	3.373041	-0.002430	5.745557

C	0.673525	2.146392	2.668157
H	1.322492	2.705306	1.976331
H	-0.370425	2.328338	2.363615
H	0.875371	1.071942	2.536275
C	2.405695	-1.198786	10.717082
C	5.687078	2.244887	10.734339
H	5.188942	2.817492	11.531793
H	6.772834	2.402733	10.844352
H	5.486076	1.174947	10.902764
C	4.306305	-1.836808	1.865055
H	3.586248	-2.642280	1.635765
H	5.309494	-2.294473	1.856627
H	4.260189	-1.110060	1.039489
C	4.004582	-1.187762	3.193429
C	3.230902	4.821674	3.333859
H	2.223516	4.855899	2.891837
H	3.916877	5.328369	2.635012
H	3.214868	5.401044	4.269599
C	3.163590	0.691086	4.519329
C	2.205344	-1.844616	12.066143
H	1.263018	-2.415798	12.105477
H	2.180576	-1.096857	12.873650
H	3.018807	-2.554447	12.299124
C	3.006305	0.728846	9.334020
C	-0.049090	1.792846	5.082375
H	0.197524	0.718529	5.085233
H	-1.100388	1.900106	4.769627
H	0.044115	2.162545	6.115254
C	2.540255	3.408650	10.422394
H	2.823508	2.887183	11.352215
C	0.869628	2.567232	4.127208
H	0.652825	3.644634	4.232868
C	5.154399	3.349069	4.108830
H	5.225105	3.891401	5.064698
H	5.838285	3.831945	3.392103
H	5.502308	2.317265	4.271105
C	3.465852	0.100535	3.284595
H	3.296752	0.663278	2.362458
C	4.456685	5.668206	6.960652
C	1.021119	3.290014	10.238416
H	0.700648	3.792546	9.312000
H	0.500491	3.767916	11.084300
H	0.696419	2.239515	10.187512

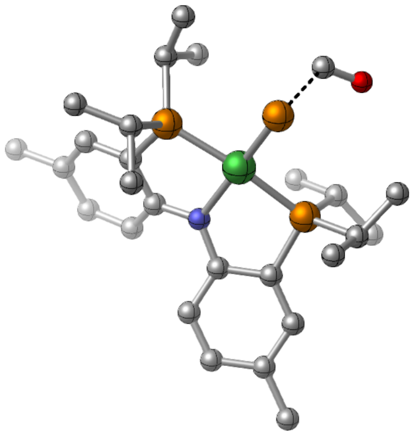

Temperature 298.150 Kelvin. Pressure 1.00000 Atm.

Zero-point correction=	0.608952 (Hartree/Particle)
Thermal correction to Energy=	0.649100
Thermal correction to Enthalpy=	0.650044
Thermal correction to Gibbs Free Energy=	0.535229
Sum of electronic and zero-point Energies=	–3713.589952
Sum of electronic and thermal Energies=	–3713.549804
Sum of electronic and thermal Enthalpies=	–3713.548860
Sum of electronic and thermal Free Energies=	–3713.663675

UB3LYP-D3/def2TZVPP-CPCM(benzene)//UB3LYP-D3/def2svp-CPCM(benzene)
E(scf) = –3716.0754 a.u.
UB3LYP-D3/6-311+g(d,p)-CPCM(benzene)//UB3LYP-D3/def2svp-CPCM(benzene)
E(scf) = –3715.8467 a.u.

H. References

1. Armarego, W. L. F.; Chai, C. *Purification of Laboratory Chemicals, 5th Edition*. Butterworth-Heinemann, 2003.
2. Pangborn, A. B.; Giardello, M. A.; Grubbs, R. H.; Rosen, R. K.; Timmers, F. J. Safe and Convenient Procedure for Solvent Purification. *Organometallics* **1996**, *15*, 1518–1520.
3. Ozerov, O. V.; Guo, C.; Fan, L.; Foxman, B. M. Oxidative Addition of N−C and N−H Bonds to Zerovalent Nickel, Palladium, and Platinum. *Organometallics* **2004**, *23*, 5573–5580.
4. Heift, D.; Benkő, Z.; Grützmacher, H. Coulomb repulsion versus cycloaddition: formation of anionic four-membered rings from sodium phosphaethynolate, Na(OCP). *Dalton Trans.* **2014**, *43*, 831–840.
5. Podewitz, M.; van Beek, J. D.; Wörle, M.; Ott, T.; Stein, D.; Rüegger, H.; Meier, B. H.; Reiher, M.; Grützmacher, H. Ion Dynamics in Confined Spaces: Sodium Ion Mobility in Icosahedral Container Molecules. *Angew. Chem. Int. Ed.* **2010**, *49*, 7465–7469.
6. Fulmer, G. R.; Miller, A. J. M.; Sherden, N. H.; Gottlieb, H. E.; Nudelman, A.; Stoltz, B. M.; Bercaw, J. E.; Goldberg, K. I. NMR Chemical Shifts of Trace Impurities: Common Laboratory Solvents, Organics, and Gases in Deuterated Solvents Relevant to the Organometallic Chemist. *Organometallics* **2010**, *29*, 2176–2179.
7. Ozarowski, A. *EPR Simulation Program*, 2023.
8. Ozarowski, A. The Zero-Field-Splitting Parameter D in Binuclear Copper(II) Carboxylates Is Negative. *Inorg. Chem.* **2008**, *47*, 9760–9762.
9. Butzlaff, C. H.; Trautwein, A. X.; Winkler, H. [15] Magnetic susceptibility. In *Methods Enzymol.*, Academic Press, 1993; Vol. 227, pp 412–437.
10. Bill, E. mpView.1.4.1, Program for Viewing and Data Import for Files from MPMS3 SQUID Magnetometer, Max-Planck Institute for Chemical Energy Conversion, Mülheim/Ruhr, 2021.
11. Bill, E. julX, Program for Simulation of Molecular Magnetic Data, Max-Planck Institute for Chemical Energy Conversion, Mülheim/Ruhr, 2008.
12. (a) Lee, C.; Yang, W.; Parr, R. G. Development of the Colle-Salvetti Correlation-Energy Formula into a Functional of the Electron Density. *Phys. Rev. B* **1988**, *37*, 785–789. (b) Becke, A. D. Density-Functional Thermochemistry. III. The Role of Exact Exchange. *J. Chem. Phys.* **1993**, *98*, 5648–5652.
13. (a) Grimme, S. Accurate description of van der Waals complexes by density functional theory including empirical corrections. *J. Comput. Chem.* **2004**, *25*, 1463–1473. (b) Grimme, S.; Antony, J.; Ehrlich, S.; Krieg, H. A consistent and accurate ab initio parametrization of density functional dispersion correction (DFT-D) for the 94 elements H-Pu. *J. Chem. Phys.* **2010**, *132*, 154104. (c) Grimme, S. Density functional theory with London dispersion corrections. *WIRES Comput. Mol. Sci.* **2011**, *1*, 211–228. (d) Ehrlich, S.; Moellmann, J.; Grimme, S. Dispersion-Corrected Density Functional Theory for Aromatic Interactions in Complex Systems. *Acc. Chem. Res.* **2012**, *46*, 916–926.
14. (a) Weigend, F.; Ahlrichs, R. Balanced basis sets of split valence, triple zeta valence and quadruple zeta valence quality for H to Rn: Design and assessment of accuracy. *Phys. Chem. Chem. Phys.* **2005**, *7*, 3297–3305. (b) Weigend, F. Accurate Coulomb-fitting basis sets for H to Rn. *Phys. Chem. Chem. Phys.* **2006**, *8*, 1057–1065.
15. (a) Klamt, A.; Schüürmann, G. COSMO: a new approach to dielectric screening in solvents with explicit expressions for the screening energy and its gradient. *J. Chem. Soc. Perkin Trans. 2* **1993**, 799–805. (b) Tomasi, J.; Persico, M. Molecular Interactions in Solution: An Overview of Methods Based on Continuous Distributions of the Solvent. *Chem. Rev.* **1994**, *94*, 2027–2094. (c) Andzelm, J.; Kölmel, C.; Klamt, A. Incorporation of solvent effects into density functional calculations of

molecular energies and geometries. *J. Chem. Phys.* **1995**, *103*, 9312–9320. (d) Barone, V.; Cossi, M. Quantum Calculation of Molecular Energies and Energy Gradients in Solution by a Conductor Solvent Model. *J. Phys. Chem. A* **1998**, *102*, 1995–2001. (e) Cossi, M.; Rega, N.; Scalmani, G.; Barone, V. Energies, structures, and electronic properties of molecules in solution with the C-PCM solvation model. *J. Comput. Chem.* **2003**, *24*, 669–681.

16. Gaussian 16, Revision C.01, Frisch, M. J.; Trucks, G. W.; Schlegel, H. B.; Scuseria, G. E.; Robb, M. A.; Cheeseman, J. R.; Scalmani, G.; Barone, V.; Petersson, G. A.; Nakatsuji, H.; Li, X.; Caricato, M.; Marenich, A. V.; Bloino, J.; Janesko, B. G.; Gomperts, R.; Mennucci, B.; Hratchian, H. P.; Ortiz, J. V.; Izmaylov, A. F.; Sonnenberg, J. L.; Williams-Young, D.; Ding, F.; Lipparini, F.; Egidi, F.; Goings, J.; Peng, B.; Petrone, A.; Henderson, T.; Ranasinghe, D.; Zakrzewski, V. G.; Gao, J.; Rega, N.; Zheng, G.; Liang, W.; Hada, M.; Ehara, M.; Toyota, K.; Fukuda, R.; Hasegawa, J.; Ishida, M.; Nakajima, T.; Honda, Y.; Kitao, O.; Nakai, H.; Vreven, T.; Throssell, K.; Montgomery, J. A., Jr.; Peralta, J. E.; Ogliaro, F.; Bearpark, M. J.; Heyd, J. J.; Brothers, E. N.; Kudin, K. N.; Staroverov, V. N.; Keith, T. A.; Kobayashi, R.; Normand, J.; Raghavachari, K.; Rendell, A. P.; Burant, J. C.; Iyengar, S. S.; Tomasi, J.; Cossi, M.; Millam, J. M.; Klene, M.; Adamo, C.; Cammi, R.; Ochterski, J. W.; Martin, R. L.; Morokuma, K.; Farkas, O.; Foresman, J. B.; Fox, D. J. Gaussian, Inc., Wallingford CT, 2016.
17. Marques, M. A. L.; Gross, E. K. U. Time-Dependent Density Functional Theory. *Annu. Rev. Phys. Chem.* **2004**, *55*, 427–455.
18. Press, W. H. Numerical recipes in FORTRAN : the art of scientific computing. 2nd ed.; Cambridge University Press: Cambridge England ; New York, NY, USA, **1992**; p 963.
19. Dennington, R.; Keith, T.; Millam, J. GaussView, Version 6.1.1., Shawnee Mission, KS, 2019.
20. Legault, C. Y. CYLview, 1.0b, Université de Sherbrooke: Sherbrooke, Canada, http://www.cylview.org, 2009.
21. Glendening, E.; Reed, A.; Carpenter, J.; Weinhold. F. NBO Version 3.1.
22. Neese, F. The ORCA program system. *WIREs Comp. Mol. Sci.* **2012**, *2*, 73–78.
23. Cossairt, B. M.; Cummins, C. C. Properties and Reactivity Patterns of AsP_3: An Experimental and Computational Study of Group 15 Elemental Molecules. *J. Am. Chem. Soc.* **2009**, *131*, 15501–15511.
24. Salvador, P.; Duran, M. The effect of counterpoise correction and relaxation energy term to the internal rotation barriers: Application to the $BF_3\cdots NH_3$ and $C_2H_4\cdots SO_2$ dimers. *J. Chem. Phys.* **1999**, *111*, 4460–4465.
25. Simon, S.; Duran, M.; Dannenberg, J. J. How does basis set superposition error change the potential surfaces for hydrogen-bonded dimers? *J. Chem. Phys.* **1996**, *105*, 11024–11031.
26. Boys, S. F.; Bernardi, F. The calculation of small molecular interactions by the differences of separate total energies. Some procedures with reduced errors. *Mol. Phys.* **1970,** *19*, 553–566.
